# Genotypic and Epidemiological Trends of Acute Gastroenteritis Associated with Noroviruses in China from 2006 to 2016

**DOI:** 10.3390/ijerph14111341

**Published:** 2017-11-03

**Authors:** Shu-Wen Qin, Ta-Chien Chan, Jian Cai, Na Zhao, Zi-Ping Miao, Yi-Juan Chen, She-Lan Liu

**Affiliations:** 1 Department of Infectious Diseases, Zhejiang Provincial Center for Disease Control and Prevention, Hangzhou 310051, China; swqin@cdc.zj.cn (S.-W.Q.); jcai@cdc.zj.cn (J.C.); zpmiao@cdc.zj.cn (Z.-P.M.); yjchen@cdc.zj.cn (Y.-J.C.); 2 Center for Geographic Information Science, Research Center for Humanities and Social Sciences, Academia Sinica, Taipei 115, Taiwan; dachianpig@gmail.com; 3 CAS Key Laboratory of Pathogenic Microbiology and Immunology, Institute of Microbiology, Chinese Academy of Sciences, Beijing 100101, China; nazhao2007@163.com

**Keywords:** norovirus, epidemiology, genotyping, acute gastroenteritis

## Abstract

There are periodical norovirus-associated acute gastroenteritis outbreaks around the world. This study aimed to analyze the molecular and epidemiological features of norovirus infections in China during 2006–2016. We extracted epidemiological data from 132 norovirus outbreaks and the norovirus genotyping for 1291 sequences in China over the past ten years. A total of 132 norovirus outbreaks (8133 cases) were reported in China, where the east and south regions were most affected [47.7% (63/132)]. The highest number of outbreaks occurred in 2015. A seasonal pattern has been observed, with a peak from November to the following March. Most of the outbreaks occurred in middle and primary schools, accounting for 28.8% (38/132), and 28.0% (37/132) of outbreaks, respectively. The dominant age group was 10 to 19 years old, responsible for 75.7% (933/1232) of cases. Generally, the dominant genotypes was GII, for 81.9% (1058/1291) of sequences. G II.4 was the predominant genotype in China from 2004 to 2014. However, the GII.17 became more prevalent starting in 2014. Norovirus-associated acute gastroenteritis increased sharply in recent years caused by the emergence of GII.17, but epidemiological features have not changed during 2006–2016. Vigilant surveillance should be strengthened to promptly detect any variation.

## 1. Introduction

Noroviruses (NoVs) are a group of non-enveloped, single-stranded positive ribonucleic acid (RNA) viruses [1]. The genome, except for murine norovirus, contains three open reading frames (ORF1, ORF2, and ORF3). ORF1 encodes a polyprotein that is post-translationally cleaved into seven nonstructural mature proteins (NS1 to NS7). ORF2 encodes the major structural protein (VP1), and ORF3 encodes a minor structural protein (VP2) [2]. Based on amino acid identity in the VP1 protein, NoVs can genetically be classified into seven different genogroups (GI, GII, GIII, GIV, GV, GVI and GVII) [2]. Those NoVs that infect humans belong to GI, GII and GIV, whereas GIII and GV infect bovine and mice species, respectively, and GVI and GVII infect canine species [2,3,4]. GII.4 account for the majority of adult outbreaks of gastroenteritis and often sweep across the globe [5,6]. Periodic increases in NoV outbreaks tend to occur in association with the emergence of new GII.4 strains that evade population immunity [7].

NoVs are the most common cause of epidemic gastroenteritis, responsible for at least 50% of all acute gastroenteritis outbreaks worldwide [8]. In 1972, norovirus was first identified from a rectal swab specimen from an acute nonbacterial gastroenteritis outbreak in Norwalk (OH, USA) in 1968 [9,10]. Norovirus outbreaks have occurred in both adult and pediatric populations across a wide range of geographic regions [9,11,12]. The pooled prevalence of NoVs in patients with acute gastroenteritis has been estimated at 20% and 19% in developed and developing countries, respectively [13]. It causes the second greatest burden of all infectious diseases worldwide, especially in low-income countries, which bear the brunt of severe outcomes of acute gastroenteritis [14]. In addition, NoVs are extremely contagious, with an estimated infectious dose as low as 10 viral particles [1], and are mostly transmitted by food, water, and contaminated environmental surfaces as well as directly from person to person, without lasting immunity to NoVs [15,16]. Outbreaks can occur in a variety of institutional settings (e.g., nursing homes, hospitals, and schools) and cruise ships [9]. Furthermore, 30% of NoV infections are asymptomatic, and asymptomatic persons can shed virus and become potential sources of transmission [17]. NoVs can affect people of all ages, especially children, the old, and the immunosuppressed [18]. NoV-associated deaths have been reported among elderly persons and in the context of outbreaks in long-term care facilities [19].

NoV infections are also common problems in China [20,21]. China’s government launched the National Notifiable Disease Reporting System (NNDRS) for notification of infectious diseases in 2003 and enhanced it in 2006. For this study, notifiable norovirus outbreaks from 2006 to 2016 in China were used to analyze the epidemiological features in the largest sample size, with the aim of identifying the specificities of high-risk areas, populations and seasons, as well as to identify the norovirus genotypes. This research will assist in planning and developing resources to decrease the norovirus outbreaks and disease burden through accurate prevention and control in the future.

## 2. Materials and Methods

### 2.1. Ethical Statement

This study was conducted according to the principles and guidelines of the Declaration of Helsinki, and was approved by the Research Ethics Committee of the Zhejiang Provincial Center for Disease Control and Prevention (T-043-R). We confirm that informed consent was obtained from all subjects before conducting this survey, and all data were supplied and analyzed in an anonymous format, with no personal identifying information provided to researchers.

### 2.2. Case Definition and Outbreak Definition

In the present study, laboratory-confirmed cases and clinically diagnosed cases were included. The following case definitions were used according to Guidelines on Outbreak Investigation, Prevention and Control of NoV Infection (2015 edition) (http://www.chinacdc.cn/jkzt/crb/qt/nrbdjxwcy/jszl_2273/201511/t20151120_122120.html):

(1) A laboratory-confirmed case of NoV infection is defined as a patient with three episodes of loose stools and/or two episodes of vomiting within 24 h, with detection of NoV in stool, rectal swabs or vomitus specimens by Reverse Transcription-Polymerase Chain Reaction (RT-PCR) nucleic acid or enzyme-linked immunosorbent assay (ELISA) antigen testing. RT-PCR and ELISA protocols showed in http://www.chinacdc.cn/jkzt/crb/qt/nrbdjxwcy/jszl_2273/201511/t2015 1120_122120.html.
(2) A clinical diagnosed NoV case is defined as a patient with three episodes of loose stools and/or two episodes of vomiting within 24 h, and with an epidemiologic link to laboratory-confirmed cases in one outbreak.
(3) An outbreak of NoV infection should satisfy the following criteria: first, the event reports 20 or more NoVs cases in 7 days; second, the event occurs in one setting, for example: a school, kindergarten, hospital, nursing home, etc.; third, the reported cases have the same exposure from a common source of infection or by person-to-person transmission; and fourth, two or more cases are laboratory-confirmed cases.

### 2.3. Data Source

#### 2.3.1. Outbreak Information

In China, acute diarrheal diseases associated with NoVs belong to Class C notifiable infectious diseases reported to the National Notifiable Disease Reporting System (NNDRS) according to the Law of the People’s Republic of China on Prevention and Treatment of Infectious Diseases issued by the Chinese Government in 2004 (http://www.npc.gov.cn/englishnpc/Law/2007-12/12/content_1383919.htm). And an outbreak should be reported to NNDRS as an emergency event by the local CDC within 2 h after confirmed norovirus is identified in 20 or more cases with the same symptom, such as diarrhea or vomiting, within 7 days in one setting, for example: a school, military facility, baby care center, etc. The epidemiological information of NoVs outbreaks in this study were obtained from the NNDRS, including province, setting, month, and case of NoV infection from 2006 to 2016.

#### 2.3.2. Sequences Information

A total of 1291 VP1 sequences were retrieved from GenBank (https://www.ncbi.nlm.nih.gov/genbank/). No sequences generated from specimens collected and analyzed in this study. The sequences were selected based on the following criteria: first, the pathogen was norovirus; second, the host was Homo sapiens; third, sequences were from China; fourth, sequences were collected during 2004 to 2016; and fifth, sequences were VP1 or complete genome sequences. The accession numbers of all sequences in this study were presented in Supplementary Table S1.

### 2.4. Data Analyses

The word “province” in this study means provincial-level administrative divisions including traditional provinces, municipalities, and autonomous regions. We used attack rate and proportion to describe the geographic distribution, temporal distribution and population distribution.

1291 VP1 sequences were used for genotyping of the present study. All sequences were prepared and aligned by BioEdit (7.0.5 version, Ibis Therapeutics, Carlsbad, CA, USA) with the Clustal W program (The Conway Institute of Biomolecular and Biomedical Research, University College Dublin, Belfield, Ireland). The reliability was calculated based on the maximum composite likelihood model. Genotypes were determined by phylogenetic analyses with the Norovirus Typing Tool (available at http://www.rivm.nl/mpf/norovirus/typingtool).

The locations of outbreaks were geocoded by the Google Map geocoding service (https:// google-developers.appspot.com/maps/documentation/javascript/examples/geocoding-simple). Then, ArcGIS (ArcMap, 10.2 version, ESRI Inc., Redlands, CA, USA) was used to generate a spatial distribution map for NoV outbreaks. In order to interactively display the various genotypes in different temporal periods and locations, we used Tableau 9.3 version (Tableau, Seattle, WA, USA) to filter out the temporal period and display the profiles of genotypes at each outbreak location.

All statistical analyses were performed using the Statistical Package for the Social Sciences (SPSS version 13.0, SPSS Inc., Chicago, IL, USA). The level of statistical significance was established as α = 0.05.

## 3. Results

### 3.1. Epidemiological Characteristics of NoVs Infection Outbreaks in China

**Figure 1:**
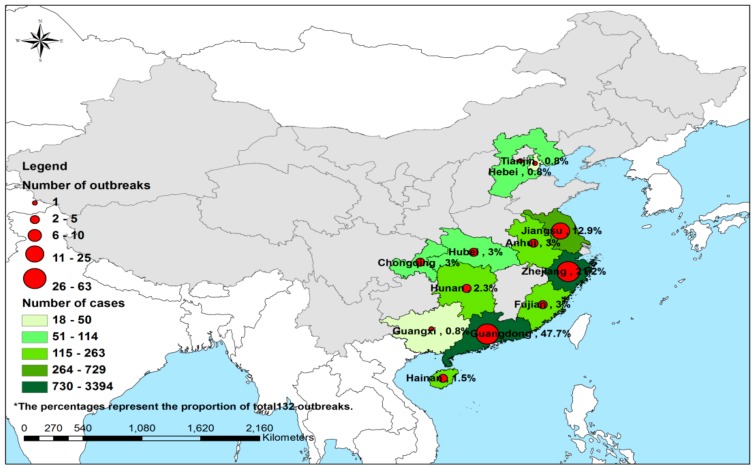
The geographical distribution of 132 outbreaks involved in 8133 cases in China during 2006 to 3 April of 2016. The shadow and the pie in the map were generated by the number of outbreaks and total cases, respectively.

As shown in Figure 1, 132 NoV outbreaks from 38.7% (12/31) of provinces were reported to the National Notifiable Disease Reporting System (NNDRS) from 2006 to 3 April 2016 in China, of which 8133 cases were identified, and 405,507 people were affected. The attack rate was 2.0% (ranging from 0.2% to 61.1%). It was found that 63.6% (84/132) of outbreaks had an attack rate <5.0%, while 27.3% (36/132) had a rate of 5–10%, and a rate >10% was found in only 6.8% (9/132) of outbreaks. The attack rate for 2.3% (3/132) of outbreaks was unknown. As for outbreak size, in 20 (15.2%), 71 (53.8%) and 41 (31.1%) of the 132 outbreaks, the case numbers were over 100, 31–100 and below 30 respectively.

#### 3.1.1. Geographic Distribution

The number of outbreaks that occurred in the various provinces from 2006 to 2016 are presented in Table 1. Three different outbreak levels were recognized, based on the total number of outbreaks during 10 years: high outbreak level (L1, >20 outbreaks), middle outbreak level (L2, 11–20 outbreaks), and low outbreak level (L3, 1–10 outbreaks). Among the 12 provinces reporting NoV outbreaks, Guangdong and Zhejiang provinces in southern and eastern China respectively were high outbreak level areas, accounting for 47.7% (63/132) and 21.2% (28/132) of the 132 reported NoV outbreaks; and Jiangsu province, in eastern China, was a middle outbreak level area, accounting for 12.9% (17/132). The other 9 provinces had a low outbreak level [Guangxi had only 0.8% of the 132 reported NoV outbreaks (1/132); Hebei 0.8% (1/132); Tianjin 0.8% (1/132); Hainan 1.5% (2/132); Hunan 2.3% (3/132); Anhui 3.0% (4/132); Fujian 3.0% (4/132); Hubei 3.0% (4/132); and Chongqing 3.0% (4/132)]. 

The number of outbreaks in 12 provinces, except Chongqing and Fujian Province, peaked in 2015. The attack rates of outbreaks in L1, L2 and L3 outbreak level areas were 1.8% (6232/339,548), 2.4% (729/30,846) and 3.3% (1172/35,113) respectively, and the difference among them was significant (*p* < 0.05).

**Figure 2:**
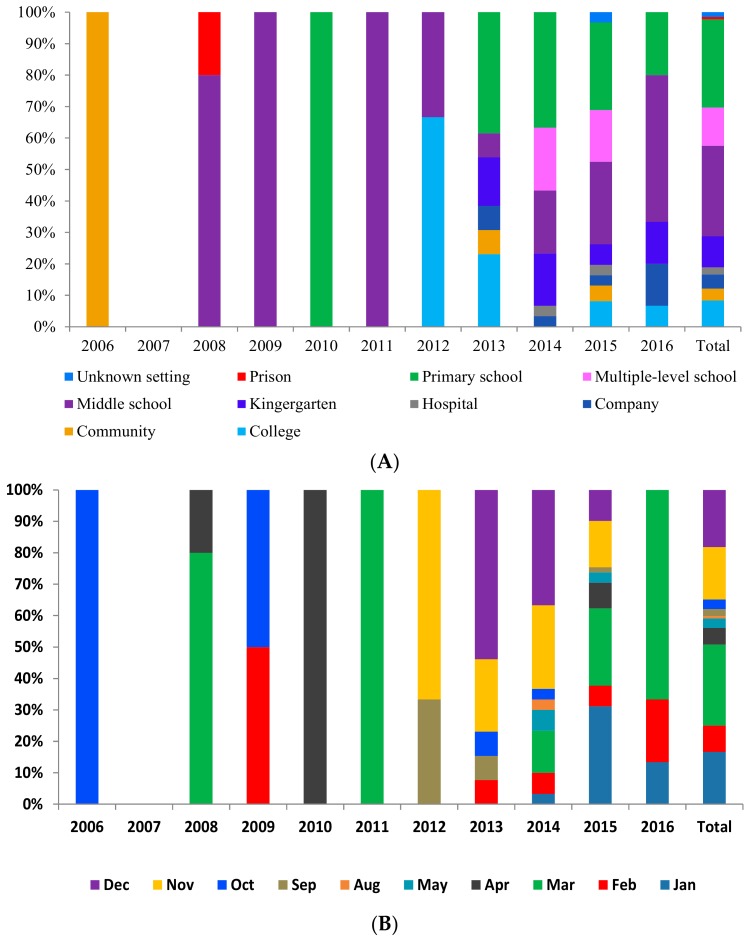

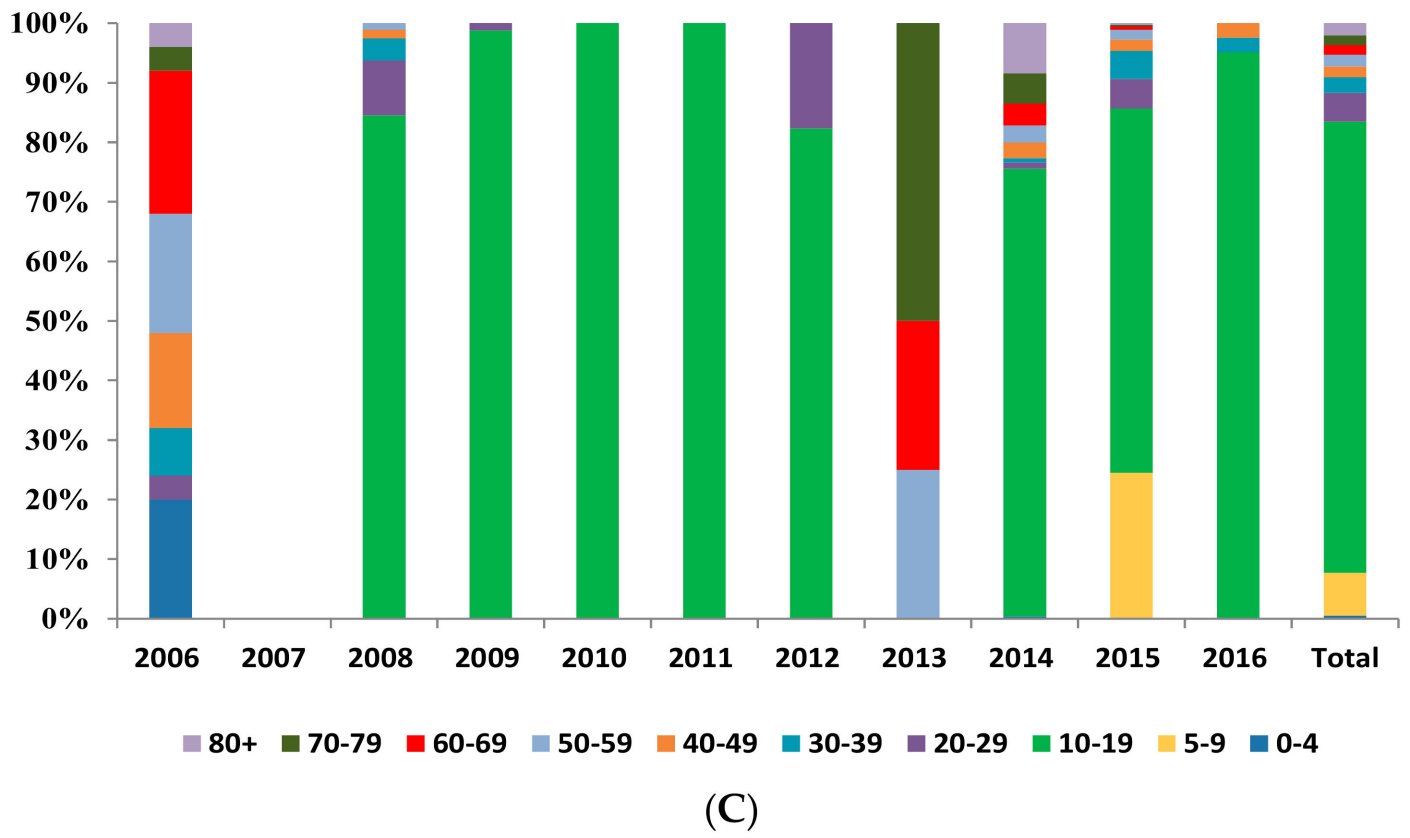
The distribution of 132 acute gastroenteritis outbreaks associated with noroviruses in China during 2006 to 3 April 2016. The bar in the figures were all generated by the number of outbreaks (*n* = 132). (**A**) Setting distribution. (**B**) Month distribution. (**C**) Age distribution.

As for outbreak settings, closed public facilities, especially schools, played a pivotal role in the NoV outbreaks. Middle schools, primary schools and multiple-level schools were the most frequent sources of NoV outbreaks, accounting for 28.8% (38/132), 28.0% (37/132) and 12.1% (16/132) of all outbreaks respectively. Kindergartens and colleges accounted for 9.8% (13/132) and 8.3% (11/132) of infections, respectively. Companies, hospitals, communities and prisons accounted for 4.5% (6/132), 2.3% (3/132), 3.8% (5/132) and 0.8% (1/132) respectively. The type of settings for 1.5% (2/132) of outbreaks was not clear. In 2006, the only one outbreak occurred in a community. After 2008, middle schools became the predominant settings, but the proportion of primary schools and multiple-level schools increased since 2013, as is shown in Figure 2A. The attack rate of outbreaks in prisons was the highest at 11.7% (147/1260), followed by kindergartens at 8.9% (384/4323), unknown settings 5.6% (237/4269), hospitals 4.5% (303/6705), primary schools 3.8% (1969/52,113), companies 3.5% (208/5923), middle schools 2.3% (2069/88,245), multiple-level schools 1.6% (1187/73,196), communities 1.3% (383/29,314) and colleges 0.9% (1246/140,159). The attack rates among the settings were significantly different (*p* < 0.05).

#### 3.1.2. Temporal Distribution

**Figure 3:**
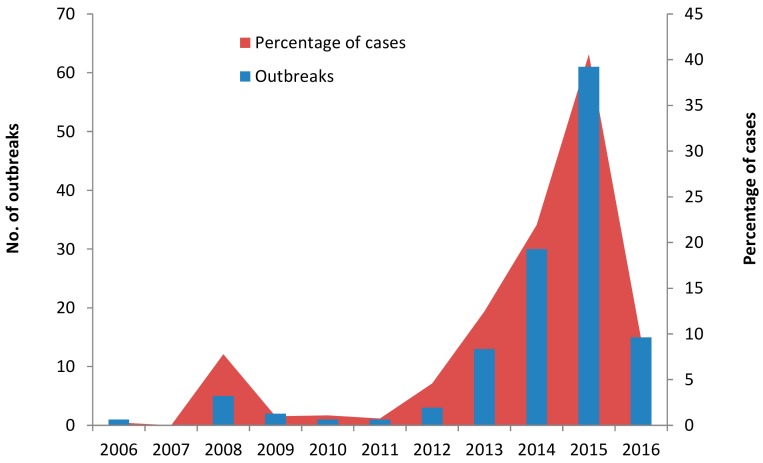
The yearly distributions of 8133 cases from 132 norovirus outbreaks in China from 2006 to 3 April 2016.

As to temporal distribution at the national scale, 0.8% (1/132) of identified outbreaks were reported in 2006, and 11.4% (15/132) were found in the first half of 2016. There was no outbreak reported in 2007. The outbreak cases showed a rising trend from 2006 to 2016, peaking in 2015, with that single year accounting for 46.2% (61/132) of the total reported outbreaks, as is shown in Figure 3.

As for seasonal distribution, NoV outbreaks were reported throughout the year during 2006–2016, with a single annual peak in winter (the combined number in November and December accounted for 34.8% (46/132) of total outbreaks) and early spring (the combined number from January to March accounted for 50.8% (67/132) of total outbreaks). In contrast, summer was the low season, in particular June and July, when no outbreaks were identified in China. In every year, the peaking months were consistent with the total trend, concentrated in winter and early spring, as is shown in Figure 2B.

#### 3.1.3. Population Distribution

Examining age distribution, cases ranged in age from nine months to 93 years, with a median age of 17 years. The age groups, ordered by the number of cases they accounted for, are: 10–19 years, 75.7% (933/1232); 5–9 years, 7.2% (89/1232); 20–29 years, 4.9% (60/1232); 30–39 years, 2.6% (32/1232); 80+ years, 2.0% (25/1232); 50–59 years, 1.9% (24/1232); 40–49 years, 1.9% (23/1232); 60–69 years and 70–79 years, each 1.6% (20/1232); and 0–5 years, 0.5% (6/1232). In 2006 and 2013, the dominant age groups were 60–69 years and 70–79 years, accounting for 24.0% (6/25) and 50.0% (4/8) of the outbreak cases. The age distribution in other years was similar during 2006 to 2016, as is shown in Figure 2C. The distribution of NoV outbreak cases from 2006 to 2016 in China was quite equal across sexes (male vs. female = 1.1:1.0). The age group difference between male and female was significant (*p* < 0.05).

### 3.2. Genotype Distribution of NoVs Infection in China

**Figure 4:**
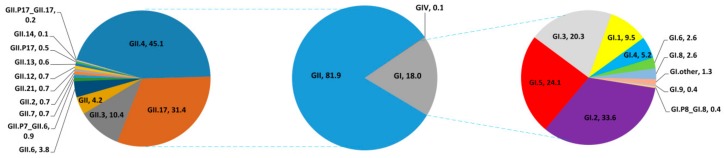
Genogroups and genotypes percentage of 1291 sequences of noroviruses isolated from 15 provinces and areas in China during 2004 to 2015. The pie in the figure was generated by the number of sequences (*n* = 1291).

Generally, the dominant NoV strain was the GII genogroup, accounting for 81.9% of sequences (1058/1291), followed by GI with 18.0% (232/1291) and GIV with 0.1% (1/1291). For the GII group, the predominant genotype was GII.4, with 45.1% (477/1058). Next was GII.17, accounting for 31.4% (332/1058), then GII.3 at 10.4% (110/1058), and GII.6 3.8% (40/1058), and the other GII genotypes accounted for 9.3% (99/1058) of total sequences. For the GI group, the major genotype was GI.2, accounting for 33.6% (78/232); GI.5 accounted for 24.1% (56/232), GI.3 20.3% (47/232), GI.1 9.5% (22/232), and other GI genotypes 12.5% (29/232) of total sequences respectively, as is shown in Figure 4.

**Figure 5:**
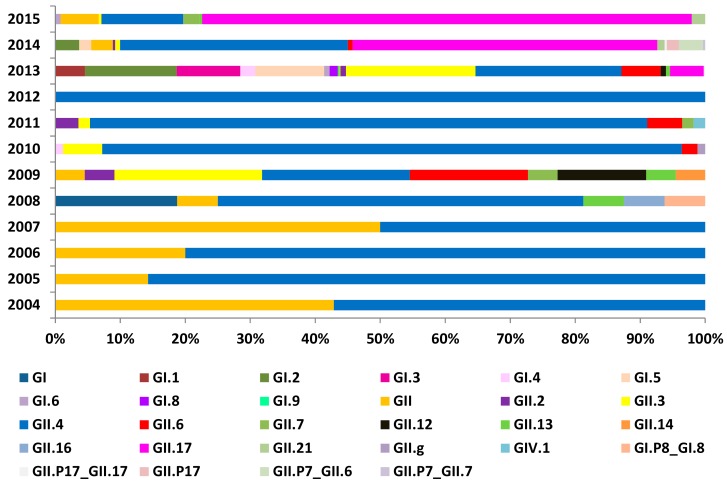
Percentage of total norovirus genotypes during 2004 to 2015. The bar in the figure was generated by the number of genotypes (*n* = 1291).

Temporal analysis indicated that the virus genotype went through two major phases of changes in China. The first phase was characterized by the predominance of genotype GII.4. GII NoV was found to exhibit genetic diversity and polymorphism, with some novel genotypes such as GII.2, GII.3, GII.6 and GII.7 isolated in China since 2008. The second phase, from 2014 till now, was when the virus genotype diverted to GII.17, which was identified as the predominant genotype, as is shown in Figure 5.

**Figure 6:**
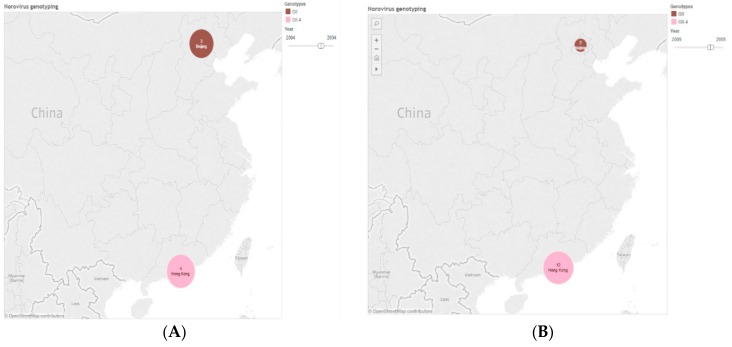

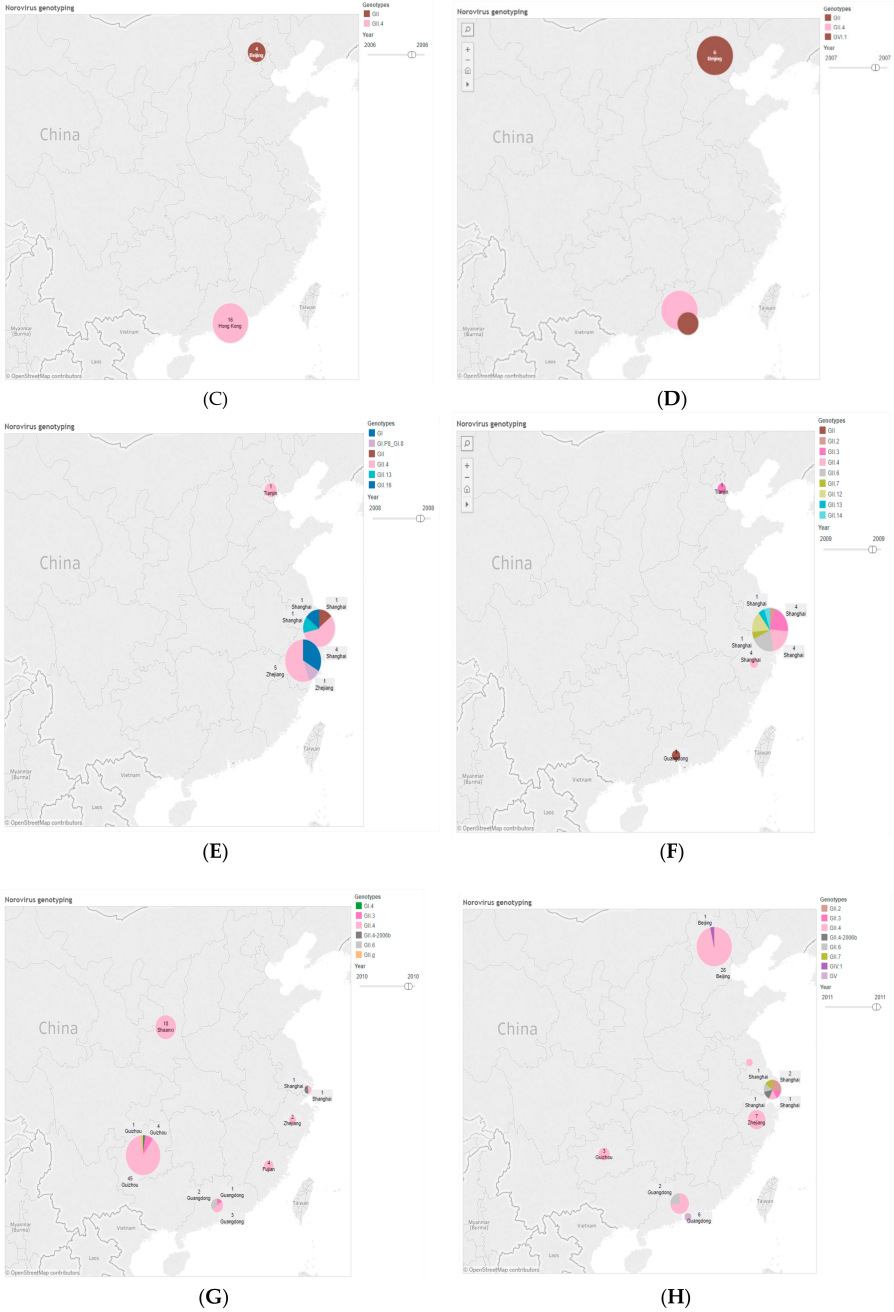

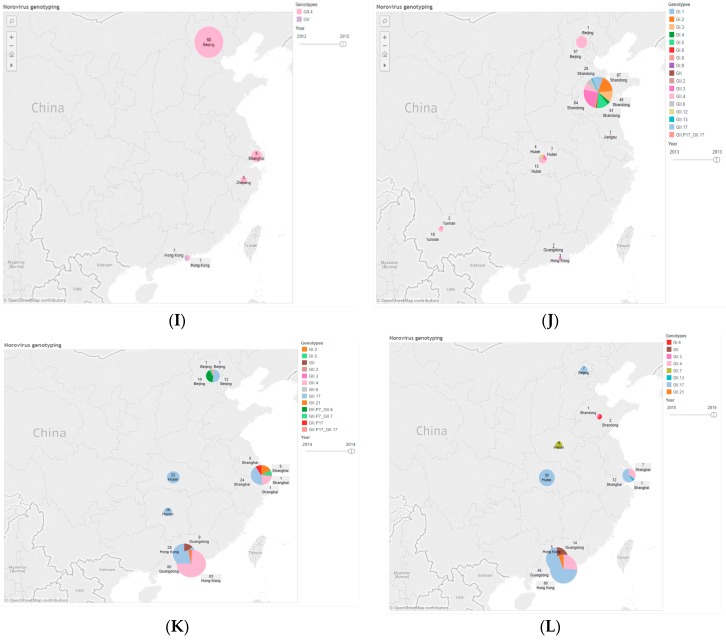
Genotyping distribution in each year according to 1291 VP1 sequences of norovirus in China during 2004 to 2015. The pie in the figures were generated by the number of genotypes (*n* = 1291). (**A**) Genotyping distribution in 2004. (**B**) Genotyping distribution in 2005. (**C**) Genotyping distribution in 2006. (**D**) Genotyping distribution in 2007. (**E**) Genotyping distribution in 2008. (**F**) Genotyping distribution in 2009. (**G**) Genotyping distribution in 2010. (**H**) Genotyping distribution in 2011. (**I**) Genotyping distribution in 2012. (**J**) Genotyping distribution in 2013. (**K**) Genotyping distribution in 2014. (**L**) Genotyping distribution in 2015.

The geographical and regional clusters were identified as varying by genotype. In the west of China, for example, in Yunnan and Guizhou Provinces, GII.4 was the main cause of NoV outbreaks, while GII.17 was the predominant strain in the center of China, including Hunan and Hubei. In contrast, GII.4 and GII.17 were co-circulating in southern and eastern China. As to temporal trend, GII.4 was the main cause of NoV outbreaks until diversity of genotype occurred in 2013, while GII.17 was the predominant strain after 2013, as is shown in Figure 6.

## 4. Discussion

NoV is a leading cause of outbreaks and sporadic cases of acute gastroenteritis in individuals of all ages worldwide [13]. In this research, we report 132 confirmed outbreaks of noroviral gastroenteritis occurring in China during the past ten years. NoV epidemic characteristics and cycles in China were remarkably similar before 2014, with a rising incidence starting from 2014. The periodic emergence of new strains of NoV may also interact with host immunity in the population to mediate annual patterns of disease [9,22]. However, the seasonality of every year observed corresponds roughly to a late winter and early spring peak, as reported in Europe and Canada [23,24]. On the one hand, studies have found associations between NoV seasonality and climatic/weather phenomena [24]. Specifically, rainfall, relative humidity and changes in temperature have been highlighted as important factors for NoV seasonality, probably due to waterborne transmission of the virus [20,25]. On the other hand, the seasonal behavior of NoV gastroenteritis is known to be influenced by host behavior. In particular, people crowding together and more time spent indoors are possible factors increasing human-to-human transmission of these viruses during winter [21,22,26]. In general, understanding the seasonal changes in NoV infection is important to be able to implement efficient surveillance and preventive measures for its control.

In terms of area distribution, a significantly higher number of NoV outbreaks happened in southern and eastern China, predominantly in population-dense Guangdong and Zhejiang provinces. The differing patterns of outbreaks may represent varying ecological situations, population density, climate, hygienic status, surveillance-control efforts, etc. [27,28]. Among these factors, the climate in southern and eastern China, was humid and cold in winter, which is helpful to the reproduction of NoVs [24,25]. A high economic development level, resulted in dense populations which are at highest risks of the spread of NoVs [21]. Also, surveillance were implemented more strictly and efficiently in southern and eastern China [29,30]. Thus, more NoV outbreaks were reported in southern and eastern China which were classified as high epidemic level areas. NoV outbreaks commonly occur in various institutional settings such as schools, daycare facilities, and nursing homes [7]. This study suggests that middle schools and primary schools are the most important settings in China. This finding is consistent with those reported in other Asian countries, including Japan and Korea [31,32], but inconsistent with reports from Europe and the US, where nursing homes and child care centers have been the predominant settings [7,33,34,35,36]. In China, middle schools and primary schools are enclosed dormitory buildings with incomplete boiled water supply, and students do not have a strong sense of personal hygiene [27]. The young students stay in crowded, enclosed classrooms where the viruses are highly transmissible by person-to-person contact [21]. One study in the schools has demonstrated that enhanced hand hygiene and thorough disinfection of surfaces can reduce the possibility of virus transmission [21].

One key observation identified in this study is that those aged between 10 to 19 years old accounted for a majority (>70%) of NoV outbreaks, with an equal sex distribution. The age and sex findings are similar in different areas, consistent with findings from previous studies in China [37,38], but inconsistent with other reports from Europe, where groups <5 years old and >65 years old were the major populations [36,39]. The reasons for the predominance of cases 10–19 years old are not understood, but have been attributed to opportunities for exposure, immunity, and susceptibility to the virus. NoV viral load has been found to be high in children [40], which clearly indicates that NoV are replication competent in children. This suggests that children are a population susceptible to this virus [41,42]. On the other hand, it has long been recognized that there is a high degree of NoV genotype diversity in children, and the binding properties of GII.4 viruses have altered over time [42]. This will result in a larger susceptible host population and a short duration of cross-protection of NoV immunity [18,43].

This study shows that from 2006 to 2016, NoV was present in a diversity of genotypes in China. The temporal analysis of genotypes shows that NoV underwent two major epidemiologic phases: the early phase showed that GII.4 absolutely predominated in China before 2014. Furthermore, the GII.4 genotypes don’t appear to be restricted by region in China [44,45]. This results are also in agreement with findings from other areas and countries; since the mid-1990s GII.4 viruses have caused the majority (70–80%) of all NoV-associated gastroenteritis outbreaks worldwide [46,47]. Notably, the emergence of novel GII.4 variants, particularly the Sydney_2012 variant strain, appears to deserve more attention [48]; it has evolved rapidly every few years, and is responsible for the increased outbreaks around the globe [49,50]. Secondly, in the last phase, other, novel GII.P17-GII.17 viruses have replaced the previously dominant GII.4 in China since 2014 [51]. The novel GII.17 is also the predominant genotype in other countries [52,53], while in Japan, a sharp increase in the number of cases caused by this novel virus has been observed during 2014–2015 [54]. However, in 2015, its genetic diversity, adaptation and fitness were also found in China, especially in high incidence areas. This situation allows us to induce two facts: one is that there is no long-lasting immunity to norovirus [19], and the other is that norovirus survived in the highly populous areas of both southern and eastern China, despite the differences in the factors of sunshine, humidity and temperature [20,29]. However, multiple genotypes are co-circulating in these areas, resulting in an increased future possibility of variant strains, which will raise the number of acute NoV outbreaks, as observed in the southern and eastern areas of China [51].

## 5. Conclusions

In conclusion, this study shows that: (i) the number of outbreaks cases rose from 2006 to 2016, peaking in 2015; (ii) middle school and primary schools were the predominant settings; (iii) the major age group was 10–19 years old; and (iv) GII.4 was the main genotype, while GII.17 was the predominant strain since 2014. In the future, several measures are needed in response. First, continuous, adequate and functioning surveillance systems are required in China for early discovery of NoV outbreaks. Second, strengthening infection-preventing hygienic measures and health education, in schools, health care facilities and the community, will cut the transmission chain. Third, improving clinical case management services, particularly for asymptomatic and mild manifestations, will decrease NoV outbreaks. Overall, our study serves as a pre-vaccination baseline against which future interventions can be compared. More broadly, further studies are warranted to elucidate the dynamics and immunity patterns of norovirus outbreaks, a rising public health problem in Asia. 

This study has several limitations. Firstly of all, the major limitation of our analysis is reporting bias, which occurred for two reasons: one is that only outbreaks of over 20 cases must be reported, while outbreaks below 20 cases were not included in this NNDRS system of China; the other reason is that the sensitivity of NNDRS systems in developed eastern areas was much higher than those in the western areas, and an increasing number of outbreaks were identified in the east areas compared to the western provinces. Secondly, we did not collect asymptotic cases because most of them did not seek medical care in hospitals. Thirdly, we cannot be sure of the actual means of transmission, whether directly from person to person or indirectly via contaminated water or food. In summary, our data are only inclusive of what has been reported and in some outbreaks publicly available, and this might lead to over- or underestimates of the findings mentioned above.

## Figures and Tables

**Table 1:** The number of acute gastroenteritis outbreaks associated with noroviruses reported in 12/31 provinces of China from 2006 to 2016.

Province	2006	2008	2009	2010	2011	2012	2013	2014	2015	2016	Total (%)
Anhui	0	0	0	0	0	0	0	1	2	1	4 (3.0)
Chongqing	0	0	0	0	0	0	0	2	0	2	4 (3.0)
Fujian	0	0	0	0	0	0	2	1	0	1	4 (3.0)
Guangdong	0	0	0	0	0	0	9	15	33	6	63 (47.7)
Guangxi	0	0	0	0	0	0	0	0	1	0	1 (0.8)
Hainan	0	0	0	0	0	0	0	0	1	1	2 (1.5)
Hebei	0	0	0	0	0	0	0	0	1	0	1 (0.8)
Hubei	0	0	0	0	0	0	0	0	4	0	4 (3.0)
Hunan	0	0	0	0	0	0	1	0	2	0	3 (2.3)
Jiangsu	0	0	0	0	0	0	0	6	9	2	17 (12.9)
Tianjin	0	0	0	0	0	0	0	0	1	0	1 (0.8)
Zhejiang	1	5	2	1	1	3	1	5	7	2	28 (21.2)
Total (%)	1 (0.8)	5 (3.8)	2 (1.5)	1 (0.8)	1 (0.8)	3 (2.3)	13 (9.8)	30 (22.7)	61 (46.2)	15 (11.3)	132 (100.0)

## Supplementary Materials

The following are available online at http://www.mdpi.com/1660-4601/14/11/1341/s1, Table S1: Accession numbers of human norovirus sequences used in this study isolated in China during 2004 to 2015 (*n* = 1291).

## Abbreviations

The following abbreviations are used in this manuscript:

NoVs	Noroviruses
RNA	Ribonucleic acid
ORF	Open reading frames
NS	Nonstructural mature proteins
NNDRS	National Notifiable Disease Reporting System
CDC	Center for Disease Control and Prevention
