# Supplementary material for: Genotypic and Epidemiological Trends of Acute Gastroenteritis Associated with Noroviruses in China from 2006 to 2016
Source: Int J Environ Res Public Health. 2017 Nov 3;14(11):1341. doi: 10.3390/ijerph14111341 (PMC5707980; doi:10.3390/ijerph14111341)
Supplement: Supplementary file 1 [file ijerph-14-01341-s001.pdf]

Supplementary Materials: Genotypic and Epidemiological Trends of Acute Gastroenteritis Associated with Noroviruses in China from 2006 to 2016

Supplementary Table 1. Accession numbers of human norovirus sequences used in this study isolated in China during 2004 to 2015 (*n*=1291).

Accession Number	Strain Name	Country	Area	Collect Year	Genotype
KP718701.1	Norovirus GII.17 isolate Hu/15F93/ZH/GD/CHN/2015	China	Guangdong	2015	GII.17
KP718700.1	Norovirus GII.17 isolate Hu/15F91/ZH/GD/CHN/2015	China	Guangdong	2015	GII.17
KP718699.1	Norovirus GII.17 isolate Hu/15F89/ZH/GD/CHN/2015	China	Guangdong	2015	GII.17
KP718698.1	Norovirus GII.17 isolate Hu/15F82/ZH/GD/CHN/2015	China	Guangdong	2015	GII.17
KP718697.1	Norovirus GII.17 isolate Hu/15F81/ZH/GD/CHN/2015	China	Guangdong	2015	GII.17
KP718696.1	Norovirus GII.17 isolate Hu/15F80/ZH/GD/CHN/2015	China	Guangdong	2015	GII.17
KP718695.1	Norovirus GII.17 isolate Hu/15F79/ZH/GD/CHN/2015	China	Guangdong	2015	GII.17
KP718694.1	Norovirus GII.17 isolate Hu/15F77/ZH/GD/CHN/2015	China	Guangdong	2015	GII.17
KP718693.1	Norovirus GII.17 isolate Hu/15F74/ZH/GD/CHN/2015	China	Guangdong	2015	GII.17
KP718692.1	Norovirus GII.17 isolate Hu/15F73/ZH/GD/CHN/2015	China	Guangdong	2015	GII.17
KP718691.1	Norovirus GII.17 isolate Hu/15F72/ZH/GD/CHN/2015	China	Guangdong	2015	GII.17
KP718690.1	Norovirus GII.17 isolate Hu/15F67/ZH/GD/CHN/2015	China	Guangdong	2015	GII.17
KP718689.1	Norovirus GII.17 isolate Hu/15F62/ZH/GD/CHN/2015	China	Guangdong	2015	GII.17
KP718688.1	Norovirus GII.17 isolate Hu/15F58/ZH/GD/CHN/2015	China	Guangdong	2015	GII.17
KP718687.1	Norovirus GII.17 isolate Hu/15F57/ZH/GD/CHN/2015	China	Guangdong	2015	GII.17
KP718686.1	Norovirus GII.17 isolate Hu/15F56/ZH/GD/CHN/2015	China	Guangdong	2015	GII.17
KP718685.1	Norovirus GII.17 isolate Hu/15F55/ZH/GD/CHN/2015	China	Guangdong	2015	GII.17

Table S1. *Cont.*

KP718684.1	Norovirus GII.17 isolate Hu/15F54/ZH/GD/CHN/2015	China	Guangdong	2015	GII.17
KP718683.1	Norovirus GII.17 isolate Hu/15F48/ZH/GD/CHN/2015	China	Guangdong	2015	GII.17
KP718682.1	Norovirus GII.17 isolate Hu/15F42/ZH/GD/CHN/2015	China	Guangdong	2015	GII.17
KP718681.1	Norovirus GII.17 isolate Hu/15F33/ZH/GD/CHN/2015	China	Guangdong	2015	GII.17
KP718680.1	Norovirus GII.17 isolate Hu/15F32/ZH/GD/CHN/2015	China	Guangdong	2015	GII.17
KP718679.1	Norovirus GII.17 isolate Hu/15F23/HZ/GD/CHN/2015	China	Guangdong	2015	GII.17
KP718678.1	Norovirus GII.17 isolate Hu/15F22/HZ/GD/CHN/2015	China	Guangdong	2015	GII.17
KP718677.1	Norovirus GII.17 isolate Hu/15F19/HZ/GD/CHN/2015	China	Guangdong	2015	GII.17
KP718676.1	Norovirus GII.17 isolate Hu/15F18/HZ/GD/CHN/2015	China	Guangdong	2015	GII.17
KP718669.1	Norovirus GII.17 isolate Hu/14F1665/DG/GD/CHN/2014	China	Guangdong	2014	GII.17
KP718668.1	Norovirus GII.17 isolate Hu/14F1651/QY/GD/CHN/2014	China	Guangdong	2014	GII.17
KP718667.1	Norovirus GII.17 isolate Hu/14F1650/QY/GD/CHN/2014	China	Guangdong	2014	GII.17
KP718666.1	Norovirus GII.17 isolate Hu/14F1649/QY/GD/CHN/2014	China	Guangdong	2014	GII.17
KP718665.1	Norovirus GII.17 isolate Hu/14F1648/QY/GD/CHN/2014	China	Guangdong	2014	GII.17
KP718664.1	Norovirus GII.17 isolate Hu/14F1646/QY/GD/CHN/2014	China	Guangdong	2014	GII.17
KP718663.1	Norovirus GII.17 isolate Hu/14F1645/QY/GD/CHN/2014	China	Guangdong	2014	GII.17
KP718662.1	Norovirus GII.17 isolate Hu/14F1539/SZ/GD/CHN/2014	China	Guangdong	2014	GII.17
KP718661.1	Norovirus GII.17 isolate Hu/14F1538/SZ/GD/CHN/2014	China	Guangdong	2014	GII.17
KP718660.1	Norovirus GII.17 isolate Hu/14F1537/SZ/GD/CHN/2014	China	Guangdong	2014	GII.17
KP718659.1	Norovirus GII.17 isolate Hu/14F1536/SZ/GD/CHN/2014	China	Guangdong	2014	GII.17
KP718658.1	Norovirus GII.17 isolate Hu/14F1535/SZ/GD/CHN/2014	China	Guangdong	2014	GII.17
KP718657.1	Norovirus GII.17 isolate Hu/14F1534/SZ/GD/CHN/2014	China	Guangdong	2014	GII.17
KP718656.1	Norovirus GII.17 isolate Hu/14F1533/SZ/GD/CHN/2014	China	Guangdong	2014	GII.17

Table S1. *Cont.*

KP718655.1	Norovirus GII.17 isolate Hu/14F1532/SZ/GD/CHN/2014	China	Guangdong	2014	GII.17
KP718654.1	Norovirus GII.17 isolate Hu/14F1531/SZ/GD/CHN/2014	China	Guangdong	2014	GII.17
KP718653.1	Norovirus GII.17 isolate Hu/14F1530/SZ/GD/CHN/2014	China	Guangdong	2014	GII.17
KP718652.1	Norovirus GII.17 isolate Hu/14F1529/SZ/GD/CHN/2014	China	Guangdong	2014	GII.17
KP718651.1	Norovirus GII.17 isolate Hu/14F1528/SZ/GD/CHN/2014	China	Guangdong	2014	GII.17
KP718650.1	Norovirus GII.17 isolate Hu/14F1527/SZ/GD/CHN/2014	China	Guangdong	2014	GII.17
KP718649.1	Norovirus GII.17 isolate Hu/14F1526/SZ/GD/CHN/2014	China	Guangdong	2014	GII.17
KP718648.1	Norovirus GII.17 isolate Hu/14F1523/SZ/GD/CHN/2014	China	Guangdong	2014	GII.17
KP718647.1	Norovirus GII.17 isolate Hu/14F1521/SZ/GD/CHN/2014	China	Guangdong	2014	GII.17
KP718646.1	Norovirus GII.17 isolate Hu/14F1520/SZ/GD/CHN/2014	China	Guangdong	2014	GII.17
KP718645.1	Norovirus GII.17 isolate Hu/14F1516/JM/GD/CHN/2014	China	Guangdong	2014	GII.17
KP718644.1	Norovirus GII.17 isolate Hu/14F1515/JM/GD/CHN/2014	China	Guangdong	2014	GII.17
KP718643.1	Norovirus GII.17 isolate Hu/14F1514/JM/GD/CHN/2014	China	Guangdong	2014	GII.17
KP718642.1	Norovirus GII.17 isolate Hu/14F1513/JM/GD/CHN/2014	China	Guangdong	2014	GII.17
KP718641.1	Norovirus GII.17 isolate Hu/14F1512/JM/GD/CHN/2014	China	Guangdong	2014	GII.17
KP718640.1	Norovirus GII.17 isolate Hu/14F1505/GZ/GD/CHN/2014	China	Guangdong	2014	GII.17
KP718639.1	Norovirus GII.17 isolate Hu/14F1503/GZ/GD/CHN/2014	China	Guangdong	2014	GII.17
KP718638.1	Norovirus GII.17 isolate Hu/14F1502/GZ/GD/CHN/2014	China	Guangdong	2014	GII.17
KJ938997.1	Norovirus Hu/GI.3/Xiangyang43/Hubei/2013/CHN isolate Xiangyang43	China	Hubei	2013	GI.3
KJ938996.1	Norovirus Hu/GII.4/Xiangyang53/Hubei/2013/CHN isolate Xiangyang53	China	Hubei	2013	GII.4
KJ938995.1	Norovirus Hu/GII.12/Xiangyang27/Hubei/2013/CHN isolate Xiangyang27	China	Hubei	2013	GII.12

Table S1. *Cont.*

KJ938994.1	Norovirus Hu/GII.12/Xiangyang21/Hubei/2013/CHN isolate Xiangyang21	China	Hubei	2013	GII.12
KJ938993.1	Norovirus Hu/GII.6/Xiangyang09/Hubei/2013/CHN isolate Xiangyang09	China	Hubei	2013	GII.6
KJ938992.1	Norovirus Hu/GII.3/Xiangyang08/Hubei/2013/CHN isolate Xiangyang08	China	Hubei	2013	GII.3
KJ938991.1	Norovirus Hu/GII.3/Xiangyang11/Hubei/2013/CHN isolate Xiangyang11	China	Hubei	2013	GII.3
KJ938990.1	Norovirus Hu/GII.3/Xiangyang07/Hubei/2013/CHN isolate Xiangyang07	China	Hubei	2013	GII.3
KJ938989.1	Norovirus GII.4/Xiangyang05/Hubei/2013/CHN isolate Xiangyang05	China	Hubei	2013	GII.4
KJ938988.1	Norovirus Hu/GII.3/Xiangyang04/Hubei/2013/CHN isolate Xiangyang04	China	Hubei	2013	GII.3
KJ938987.1	Norovirus Hu/GII.4/Xiangyang02/Hubei/2013/CHN isolate Xiangyang02	China	Hubei	2013	GII.4
KJ938986.1	Norovirus Hu/GII.12/Xiangyang01/Hubei/2013/CHN isolate Xiangyang01	China	Hubei	2013	GII.12
KJ938985.1	Norovirus Hu/GII.3/Xiangyang108/Hubei/2013/CHN isolate Xiangyang108	China	Hubei	2013	GII.3
KJ938984.1	Hu/GII.4/Xiangyang97/Hubei/2013/CHN isolate Xiangyang97	China	Hubei	2013	GII.4
KJ938983.1	Hu/GII.4/Xiangyang93/Hubei/2013/CHN isolate Xiangyang93	China	Hubei	2013	GII.4
KJ938982.1	Hu/GII.6/Xiangyang88/Hubei/2013/CHN isolate Xiangyang88	China	Hubei	2013	GII.6
KJ938981.1	Hu/GII.4/Xiangyang86/Hubei/2013/CHN isolate Xiangyang86	China	Hubei	2013	GII.4
KJ938980.1	Hu/GII.3/Xiangyang84/Hubei/2013/CHN isolate Xiangyang84	China	Hubei	2013	GII.3
KJ938979.1	Hu/GII.4/Xiangyang76/Hubei/2013/CHN isolate Xiangyang76	China	Hubei	2013	GII.4
KJ938978.1	Hu/GII.4/Xiangyang74/Hubei/2013/CHN isolate Xiangyang74	China	Hubei	2013	GII.4

Table S1. *Cont.*

KJ938977.1	Norovirus Hu/GII.12/Xiangyang73/Hubei/2013/CHN isolate Xiangyang73	China	Hubei	2013	GII.12
KJ938976.1	Hu/GII.4/Xiangyang71/Hubei/2013/CHN	China	Hubei	2013	GII.4
KJ938975.1	Hu/GII.4/Xiangyang60/Hubei/2013/CHN	China	Hubei	2013	GII.4
KJ938974.1	Hu/GII.6/Xiangyang32/Hubei/2013/CHN	China	Hubei	2013	GII.6
KJ938973.1	Hu/GII.4/Xiangyang13/Hubei/2013/CHN	China	Hubei	2013	GII.4
KJ938972.1	Hu/GII.4/Xiangyang12/Hubei/2013/CHN	China	Hubei	2013	GII.4
KC456073.1	Norovirus Hu/GII.4/VP1451/Shanghai/2012/CHN	China	Shanghai	2012	GII.4
KC456072.1	Norovirus Hu/GII.4/VP1281/Shanghai/2012/CHN	China	Shanghai	2012	GII.4
KC456071.1	Norovirus Hu/GII.4/VP1214/Shanghai/2012/CHN	China	Shanghai	2012	GII.4
KC456070.1	Norovirus Hu/GII.4/VP1172/Shanghai/2012/CHN	China	Shanghai	2012	GII.4
KP407450.1	Norovirus GI strain Hu/CHN/2008/GI.P8_GI.8/Huzhou/N10	China	Zhejiang	2008	GI.P8_GI.8
KP407451.1	Norovirus GI strain GI/Hu/CHN/2008/Huzhou/N11	China	Zhejiang	2008	GI
KP998539.1	Norovirus GII.17 isolate GII/Hu/HKG/2014/GII.17/CUHK-NS-463	China	Hong Kong	2014	GII.17
KR020503.1	Norovirus Hu/GII.17/41621/Guangzhou/2014/CHN	China	Guangdong	2014	GII.17
KP994318.1	Norovirus GII.17 isolate Hu/NoV/GD1697/2014/Guangdong/China	China	Guangdong	2014	GII.17
KJ716369.1	Norovirus Hu/GII.4/Beijing isolate PKUPH-63/Outpatient	China	Beijing	2012	GII.4
KJ716368.1	Norovirus Hu/GII.4/Beijing isolate PKUPH-160/Outpatient	China	Beijing	2013	GII.4
KJ716367.1	Norovirus Hu/GII.4/Beijing isolate PKUPH-159/Outpatient	China	Beijing	2013	GII.4

Table S1. *Cont*.

KJ716366.1	Norovirus Hu/GII.4/Beijing isolate PKUPH-140/Outpatient	China	Beijing	2013	GII.4
KJ716365.1	Norovirus Hu/GII.4/Beijing isolate PKUPH-129/Outpatient	China	Beijing	2013	GII.4
KJ716364.1	Norovirus Hu/GII.4/Beijing isolate PKUPH-124/Outpatient	China	Beijing	2013	GII.4
KJ716363.1	Norovirus Hu/GII.4/Beijing isolate PKUPH-118/Outpatient	China	Beijing	2013	GII.4
KJ716362.1	Norovirus Hu/GII.4/Beijing isolate PKUPH-117/Outpatient	China	Beijing	2013	GII.4
KJ716361.1	Norovirus Hu/GII.4/Beijing isolate PKUPH-97/Outpatient	China	Beijing	2012	GII.4
KJ716360.1	Norovirus Hu/GII.4/Beijing isolate PKUPH-78/Outpatient	China	Beijing	2012	GII.4
KJ716359.1	Norovirus Hu/GII.4/Beijing isolate PKUPH-64/Outpatient	China	Beijing	2012	GII.4
KJ716358.1	Norovirus Hu/GII.4/Beijing isolate PKUPH-40/Outpatient	China	Beijing	2012	GII.4
KJ716357.1	Norovirus Hu/GII.4/Beijing isolate PKUPH-39/Outpatient	China	Beijing	2012	GII.4
KJ716356.1	Norovirus Hu/GII.4/Beijing isolate PKUPH-37/Outpatient	China	Beijing	2012	GII.4
KJ716355.1	Norovirus Hu/GII.4/Beijing isolate PKUPH-20/Outpatient	China	Beijing	2012	GII.4
KJ716354.1	Norovirus Hu/GII.4/Beijing isolate PKUPH-19/Outpatient	China	Beijing	2012	GII.4
KJ678154.1	Norovirus Hu/GII.4/Beijing/PKUPH-07-05/inpatient/2013/CHN	China	Beijing	2013	GII.4
KJ678153.1	Norovirus Hu/GII.4/Beijing/PKUPH-07-04/inpatient/2013/CHN	China	Beijing	2013	GII.4
KJ678152.1	Norovirus Hu/GII.4/Beijing/PKUPH-07-03/inpatient/2013/CHN	China	Beijing	2013	GII.4
KJ678151.1	Norovirus Hu/GII.4/Beijing/PKUPH-07-02/inpatient/2013/CHN	China	Beijing	2013	GII.4
KJ678150.1	Norovirus Hu/GII.4/Beijing/PKUPH-07-01/inpatient/2013/CHN	China	Beijing	2013	GII.4
KJ678149.1	Norovirus Hu/GII.4/Beijing/PKUPH-05-07/inpatient/2013/CHN	China	Beijing	2013	GII.4

Table S1. *Cont.*

KJ678148.1	Norovirus Hu/GII.4/Beijing/PKUPH-05-06/inpatient/2013/CHN	China	Beijing	2013	GII.4
KJ678147.1	Norovirus Hu/GII.4/Beijing/PKUPH-05-05/inpatient/2013/CHN	China	Beijing	2013	GII.4
KJ678146.1	Norovirus Hu/GII.4/Beijing/PKUPH-05-04/inpatient/2013/CHN	China	Beijing	2013	GII.4
KJ678145.1	Norovirus Hu/GII.4/Beijing/PKUPH-05-03/inpatient/2013/CHN	China	Beijing	2013	GII.4
KJ678144.1	Norovirus Hu/GII.4/Beijing/PKUPH-05-02/inpatient/2013/CHN	China	Beijing	2013	GII.4
KJ678143.1	Norovirus Hu/GII.4/Beijing/PKUPH-05-01/inpatient/2013/CHN	China	Beijing	2013	GII.4
KJ678142.1	Norovirus Hu/GII.4/Beijing/PKUPH-02-09/inpatient/2013/CHN	China	Beijing	2013	GII.4
KJ678141.1	Norovirus Hu/GII.4/Beijing/PKUPH-02-08/inpatient/2013/CHN	China	Beijing	2013	GII.4
KJ678140.1	Norovirus Hu/GII.4/Beijing/PKUPH-02-07/inpatient/2013/CHN	China	Beijing	2013	GII.4
KJ678139.1	Norovirus Hu/GII.4/Beijing/PKUPH-02-06/inpatient/2013/CHN	China	Beijing	2013	GII.4
KJ678138.1	Norovirus Hu/GII.4/Beijing/PKUPH-02-05/inpatient/2013/CHN	China	Beijing	2013	GII.4
KJ678137.1	Norovirus Hu/GII.4/Beijing/PKUPH-02-04/inpatient/2013/CHN	China	Beijing	2013	GII.4
KJ678136.1	Norovirus Hu/GII.4/Beijing/PKUPH-02-03/inpatient/2012/CHN	China	Beijing	2012	GII.4
KJ678135.1	Norovirus Hu/GII.4/Beijing/PKUPH-02-02/inpatient/2012/CHN	China	Beijing	2012	GII.4
KJ678134.1	Norovirus Hu/GII.4/Beijing/PKUPH-02-01/inpatient/2012/CHN	China	Beijing	2012	GII.4

Table S1. *Cont*.

KJ465092.1	Norovirus Hu/GII.4/13R21/2013/CHN	China	Beijing	2013	GII.4
KJ465091.1	Norovirus Hu/GII.4/XW-YJH/2011/CHN	China	Beijing	2011	GII.4
KJ465090.1	Norovirus Hu/GII.4/XW-SYT/2011/CHN	China	Beijing	2011	GII.4
KJ465089.1	Norovirus Hu/GII.4/XW-MLX/2011/CHN	China	Beijing	2011	GII.4
KJ465088.1	Norovirus Hu/GII.4/S6/2012/CHN	China	Beijing	2012	GII.4
KJ465087.1	Norovirus Hu/GII.4/R3/2013/CHN	China	Beijing	2013	GII.4
KJ465086.1	Norovirus Hu/GII.4/PGZT/2011/CHN	China	Beijing	2011	GII.4
KJ465085.1	Norovirus Hu/GII.4/PGWFZ/2011/CHN	China	Beijing	2011	GII.4
KJ465084.1	Norovirus Hu/GII.4/HR99/2012/CHN	China	Beijing	2012	GII.4
KJ465083.1	Norovirus Hu/GII.4/HR96/2012/CHN	China	Beijing	2012	GII.4
KJ465082.1	Norovirus Hu/GII.4/HD-ZLY/2011/CHN	China	Beijing	2011	GII.4
KJ465081.1	Norovirus Hu/GII.4/HD-TYM/2011/CHN	China	Beijing	2011	GII.4
KJ465080.1	Norovirus Hu/GII.4/HD-LWX/2011/CHN	China	Beijing	2011	GII.4
KJ465079.1	Norovirus Hu/GII.4/FT-SCS/2011/CHN	China	Beijing	2011	GII.4
KJ465078.1	Norovirus Hu/GII.4/DX1301-14/2013/CHN	China	Beijing	2013	GII.4
KJ465077.1	Norovirus Hu/GII.4/DX12-4/2013/CHN	China	Beijing	2013	GII.4
KJ465076.1	Norovirus Hu/GII.4/DX10-9/2012/CHN	China	Beijing	2012	GII.4
KJ465075.1	Norovirus Hu/GII.4/DX10-10/2012/CHN	China	Beijing	2012	GII.4
KJ465074.1	Norovirus Hu/GII.4/CYWC/2012/CHN	China	Beijing	2012	GII.4
KJ465072.1	Norovirus Hu/GII.4/CWWBT/2012/CHN	China	Beijing	2012	GII.4
KJ465071.1	Norovirus Hu/GII.4/CWSDH/2012/CHN	China	Beijing	2012	GII.4
KJ465070.1	Norovirus Hu/GII.4/CWRS/2012/CHN	China	Beijing	2012	GII.4
KJ465069.1	Norovirus Hu/GII.4/CWLMH/2012/CHN	China	Beijing	2012	GII.4

Table S1. *Cont.*

KJ465068.1	Norovirus Hu/GII.4/CWLGC/2012/CHN	China	Beijing	2012	GII.4
KJ465067.1	Norovirus Hu/GII.4/cw7/2012/CHN	China	Beijing	2012	GII.4
KJ465066.1	Norovirus Hu/GII.4/cw5/2012/CHN	China	Beijing	2012	GII.4
KJ465065.1	Norovirus Hu/GII.4/CW-ZYX/2011/CHN	China	Beijing	2011	GII.4
KJ465064.1	Norovirus Hu/GII.4/CW-WYL/2011/CHN	China	Beijing	2011	GII.4
KJ465063.1	Norovirus Hu/GII.4/CW-WH/2011/CHN	China	Beijing	2011	GII.4
KJ465062.1	Norovirus Hu/GII.4/CLX/2012/CHN	China	Beijing	2012	GII.4
KJ465061.1	Norovirus Hu/GII.4/13LGX/2013/CHN	China	Beijing	2013	GII.4
KJ465060.1	Norovirus Hu/GII.4/13291009/2013/CHN	China	Beijing	2013	GII.4
KJ465059.1	Norovirus Hu/GII.4/13281011/2013/CHN	China	Beijing	2013	GII.4
KJ465058.1	Norovirus Hu/GII.4/13281007/2013/CHN	China	Beijing	2013	GII.4
KJ465057.1	Norovirus Hu/GII.4/13141022/2013/CHN	China	Beijing	2013	GII.4
KJ465056.1	Norovirus Hu/GII.4/13141015/2013/CHN	China	Beijing	2013	GII.4
KJ465055.1	Norovirus Hu/GII.4/13141013/2013/CHN	China	Beijing	2013	GII.4
KJ465054.1	Norovirus Hu/GII.4/13112018/2013/CHN	China	Beijing	2013	GII.4
KJ465053.1	Norovirus Hu/GII.4/13091002/2013/CHN	China	Beijing	2013	GII.4
KJ465052.1	Norovirus Hu/GII.4/13071236/2013/CHN	China	Beijing	2013	GII.4
KJ465051.1	Norovirus Hu/GII.4/13061031/2013/CHN	China	Beijing	2013	GII.4
KJ465050.1	Norovirus Hu/GII.4/13061022/2013/CHN	China	Beijing	2013	GII.4
KJ465049.1	Norovirus Hu/GII.4/13041020/2013/CHN	China	Beijing	2013	GII.4
KJ465048.1	Norovirus Hu/GII.4/13011024/2013/CHN	China	Beijing	2013	GII.4
KJ465047.1	Norovirus Hu/GII.4/13011017/2013/CHN	China	Beijing	2013	GII.4
KJ465046.1	Norovirus Hu/GII.4/13011004/2013/CHN	China	Beijing	2013	GII.4

Table S1. *Cont*.

KJ465045.1	Norovirus Hu/GII.4/12R8/2012/CHN	China	Beijing	2012	GII.4
KJ465044.1	Norovirus Hu/GII.4/12R6/2012/CHN	China	Beijing	2012	GII.4
KJ465043.1	Norovirus Hu/GII.4/12281114/2012/CHN	China	Beijing	2012	GII.4
KJ465042.1	Norovirus Hu/GII.4/12281109/2012/CHN	China	Beijing	2012	GII.4
KJ465041.1	Norovirus Hu/GII.4/12281094/2012/CHN	China	Beijing	2012	GII.4
KJ465040.1	Norovirus Hu/GII.4/12281003/2012/CHN	China	Beijing	2012	GII.4
KJ465039.1	Norovirus Hu/GII.4/12142035/2012/CHN	China	Beijing	2012	GII.4
KJ465038.1	Norovirus Hu/GII.4/12142032/2012/CHN	China	Beijing	2012	GII.4
KJ465037.1	Norovirus Hu/GII.4/12141070/2012/CHN	China	Beijing	2012	GII.4
KJ465036.1	Norovirus Hu/GII.4/12131112/2012/CHN	China	Beijing	2012	GII.4
KJ465035.1	Norovirus Hu/GII.4/12131110/2012/CHN	China	Beijing	2012	GII.4
KJ465034.1	Norovirus Hu/GII.4/12112113/2012/CHN	China	Beijing	2012	GII.4
KJ465033.1	Norovirus Hu/GII.4/12112093/2012/CHN	China	Beijing	2012	GII.4
KJ465032.1	Norovirus Hu/GII.4/12092053/2012/CHN	China	Beijing	2012	GII.4
KJ465031.1	Norovirus Hu/GII.4/12091051/2012/CHN	China	Beijing	2012	GII.4
KJ465030.1	Norovirus Hu/GII.4/12081098/2012/CHN	China	Beijing	2012	GII.4
KJ465029.1	Norovirus Hu/GII.4/12081093/2012/CHN	China	Beijing	2012	GII.4
KJ465028.1	Norovirus Hu/GII.4/12078FY-1/2012/CHN	China	Beijing	2012	GII.4
KJ465027.1	Norovirus Hu/GII.4/12071234/2012/CHN	China	Beijing	2012	GII.4
KJ465026.1	Norovirus Hu/GII.4/12061119/2012/CHN	China	Beijing	2012	GII.4
KJ465025.1	Norovirus Hu/GII.4/12041121/2012/CHN	China	Beijing	2012	GII.4
KJ465024.1	Norovirus Hu/GII.4/12041104/2012/CHN	China	Beijing	2012	GII.4
KJ465023.1	Norovirus Hu/GII.4/12041095/2012/CHN	China	Beijing	2012	GII.4

Table S1. *Cont.*

KJ465022.1	Norovirus Hu/GII.4/12041058/2012/CHN	China	Beijing	2012	GII.4
KJ465021.1	Norovirus Hu/GII.4/12012091/2012/CHN	China	Beijing	2012	GII.4
KJ465020.1	Norovirus Hu/GII.4/12012059/2012/CHN	China	Beijing	2012	GII.4
KJ465019.1	Norovirus Hu/GII.4/12011114/2012/CHN	China	Beijing	2012	GII.4
KJ465018.1	Norovirus Hu/GII.4/12011106/2012/CHN	China	Beijing	2012	GII.4
KJ465017.1	Norovirus Hu/GII.4/11281119/2011/CHN	China	Beijing	2011	GII.4
KJ465016.1	Norovirus Hu/GII.4/11281117/2011/CHN	China	Beijing	2011	GII.4
KJ465015.1	Norovirus Hu/GII.4/11142034/2011/CHN	China	Beijing	2011	GII.4
KJ465014.1	Norovirus Hu/GII.4/11142019/2011/CHN	China	Beijing	2011	GII.4
KJ465013.1	Norovirus Hu/GII.4/11141035/2011/CHN	China	Beijing	2011	GII.4
KJ465012.1	Norovirus Hu/GII.4/11092055/2011/CHN	China	Beijing	2011	GII.4
KJ465011.1	Norovirus Hu/GII.4/11092052/2011/CHN	China	Beijing	2011	GII.4
KJ465010.1	Norovirus Hu/GII.4/11092045/2011/CHN	China	Beijing	2011	GII.4
KJ465009.1	Norovirus Hu/GII.4/11092042/2011/CHN	China	Beijing	2011	GII.4
KJ465008.1	Norovirus Hu/GII.4/11091037/2011/CHN	China	Beijing	2011	GII.4
KJ465007.1	Norovirus Hu/GII.4/11091034/2011/CHN	China	Beijing	2011	GII.4
KJ465006.1	Norovirus Hu/GII.4/11091017/2011/CHN	China	Beijing	2011	GII.4
KJ465005.1	Norovirus Hu/GII.4/11071223/2012/CHN	China	Beijing	2012	GII.4
KJ465004.1	Norovirus Hu/GII.4/11071219/2012/CHN	China	Beijing	2012	GII.4
KJ465003.1	Norovirus Hu/GII.4/11071217/2012/CHN	China	Beijing	2012	GII.4
KJ465002.1	Norovirus Hu/GII.4/11071215/2012/CHN	China	Beijing	2012	GII.4
KJ465001.1	Norovirus Hu/GII.4/11011095/2011/CHN	China	Beijing	2011	GII.4
KJ465000.1	Norovirus Hu/GII.4/11011090/2011/CHN	China	Beijing	2011	GII.4

Table S1. *Cont.*

KJ464999.1	Norovirus Hu/GII.4/11011082/2011/CHN	China	Beijing	2011	GII.4
KJ464998.1	Norovirus Hu/GII.4/11011081/2011/CHN	China	Beijing	2011	GII.4
KJ464997.1	Norovirus Hu/GII.4/11011075/2011/CHN	China	Beijing	2011	GII.4
KR107934.1	Norovirus GI.2 isolate sewage/SD7506/2013/CHN	China	Shandong	2013	GI.2
KR107933.1	Norovirus GI.2 isolate sewage/SD7503/2013/CHN	China	Shandong	2013	GI.2
KR107932.1	Norovirus GI.2 isolate sewage/SD7107/2013/CHN	China	Shandong	2013	GI.2
KR107931.1	Norovirus GI.2 isolate sewage/SD6901/2013/CHN	China	Shandong	2013	GI.2
KR107930.1	Norovirus GI.2 isolate sewage/SD6807/2013/CHN	China	Shandong	2013	GI.2
KR107929.1	Norovirus GI.9 isolate sewage/SD7201/2013/CHN	China	Shandong	2013	GI.9
KR107928.1	Norovirus GI.8 isolate sewage/SD1408/2013/CHN	China	Shandong	2013	GI.8
KR107927.1	Norovirus GI.8 isolate sewage/SD1406/2013/CHN	China	Shandong	2013	GI.8
KR107926.1	Norovirus GI.8 isolate sewage/SD1309/2013/CHN	China	Shandong	2013	GI.8
KR107925.1	Norovirus GI.8 isolate sewage/SD7208/2013/CHN	China	Shandong	2013	GI.8
KR107924.1	Norovirus GI.8 isolate sewage/SD6704/2013/CHN	China	Shandong	2013	GI.8
KR107923.1	Norovirus GI.8 isolate sewage/SD6603/2013/CHN	China	Shandong	2013	GI.8
KR107922.1	Norovirus GI.6 isolate sewage/SD1508/2013/CHN	China	Shandong	2013	GI.6
KR107921.1	Norovirus GI.6 isolate sewage/SD0210/2013/CHN	China	Shandong	2013	GI.6
KR107920.1	Norovirus GI.6 isolate sewage/SD6710/2013/CHN	China	Shandong	2013	GI.6
KR107919.1	Norovirus GI.6 isolate sewage/SD7507/2013/CHN	China	Shandong	2013	GI.6
KR107918.1	Norovirus GI.5 isolate sewage/SD0108/2013/CHN	China	Shandong	2013	GI.5
KR107917.1	Norovirus GI.5 isolate sewage/SD0103/2013/CHN	China	Shandong	2013	GI.5
KR107916.1	Norovirus GI.5 isolate sewage/SD6808/2013/CHN	China	Shandong	2013	GI.5
KR107915.1	Norovirus GI.5 isolate sewage/SD7608/2013/CHN	China	Shandong	2013	GI.5

Table S1. *Cont.*

KR107914.1	Norovirus GI.5 isolate sewage/SD7604/2013/CHN	China	Shandong	2013	GI.5
KR107913.1	Norovirus GI.5 isolate sewage/SD7510/2013/CHN	China	Shandong	2013	GI.5
KR107912.1	Norovirus GI.5 isolate sewage/SD7505/2013/CHN	China	Shandong	2013	GI.5
KR107911.1	Norovirus GI.5 isolate sewage/SD7504/2013/CHN	China	Shandong	2013	GI.5
KR107910.1	Norovirus GI.5 isolate sewage/SD7406/2013/CHN	China	Shandong	2013	GI.5
KR107909.1	Norovirus GI.5 isolate sewage/SD7302/2013/CHN	China	Shandong	2013	GI.5
KR107908.1	Norovirus GI.5 isolate sewage/SD7205/2013/CHN	China	Shandong	2013	GI.5
KR107907.1	Norovirus GI.5 isolate sewage/SD7106/2013/CHN	China	Shandong	2013	GI.5
KR107906.1	Norovirus GI.5 isolate sewage/SD7104/2013/CHN	China	Shandong	2013	GI.5
KR107905.1	Norovirus GI.5 isolate sewage/SD7102/2013/CHN	China	Shandong	2013	GI.5
KR107904.1	Norovirus GI.5 isolate sewage/SD7009/2013/CHN	China	Shandong	2013	GI.5
KR107903.1	Norovirus GI.5 isolate sewage/SD7006/2013/CHN	China	Shandong	2013	GI.5
KR107902.1	Norovirus GI.5 isolate sewage/SD6910/2013/CHN	China	Shandong	2013	GI.5
KR107901.1	Norovirus GI.5 isolate sewage/SD6909/2013/CHN	China	Shandong	2013	GI.5
KR107900.1	Norovirus GI.5 isolate sewage/SD6907/2013/CHN	China	Shandong	2013	GI.5
KR107899.1	Norovirus GI.5 isolate sewage/SD6905/2013/CHN	China	Shandong	2013	GI.5
KR107898.1	Norovirus GI.5 isolate sewage/SD6903/2013/CHN	China	Shandong	2013	GI.5
KR107897.1	Norovirus GI.5 isolate sewage/SD6703/2013/CHN	China	Shandong	2013	GI.5
KR107896.1	Norovirus GI.5 isolate sewage/SD6605/2013/CHN	China	Shandong	2013	GI.5
KR107895.1	Norovirus GI.5 isolate sewage/SD1506/2013/CHN	China	Shandong	2013	GI.5
KR107894.1	Norovirus GI.5 isolate sewage/SD1306/2013/CHN	China	Shandong	2013	GI.5
KR107893.1	Norovirus GI.5 isolate sewage/SD1305/2013/CHN	China	Shandong	2013	GI.5
KR107892.1	Norovirus GI.5 isolate sewage/SD1304/2013/CHN	China	Shandong	2013	GI.5

Table S1. *Cont.*

KR107891.1	Norovirus GI.5 isolate sewage/SD1303/2013/CHN	China	Shandong	2013	GI.5
KR107890.1	Norovirus GI.5 isolate sewage/SD1302/2013/CHN	China	Shandong	2013	GI.5
KR107889.1	Norovirus GI.5 isolate sewage/SD1301/2013/CHN	China	Shandong	2013	GI.5
KR107888.1	Norovirus GI.5 isolate sewage/SD1208/2013/CHN	China	Shandong	2013	GI.5
KR107887.1	Norovirus GI.5 isolate sewage/SD1206/2013/CHN	China	Shandong	2013	GI.5
KR107886.1	Norovirus GI.5 isolate sewage/SD1205/2013/CHN	China	Shandong	2013	GI.5
KR107885.1	Norovirus GI.5 isolate sewage/SD1204/2013/CHN	China	Shandong	2013	GI.5
KR107884.1	Norovirus GI.5 isolate sewage/SD1202/2013/CHN	China	Shandong	2013	GI.5
KR107883.1	Norovirus GI.5 isolate sewage/SD1201/2013/CHN	China	Shandong	2013	GI.5
KR107882.1	Norovirus GI.5 isolate sewage/SD1106/2013/CHN	China	Shandong	2013	GI.5
KR107881.1	Norovirus GI.5 isolate sewage/SD1105/2013/CHN	China	Shandong	2013	GI.5
KR107880.1	Norovirus GI.5 isolate sewage/SD1102/2013/CHN	China	Shandong	2013	GI.5
KR107879.1	Norovirus GI.5 isolate sewage/SD0909/2013/CHN	China	Shandong	2013	GI.5
KR107878.1	Norovirus GI.5 isolate sewage/SD0907/2013/CHN	China	Shandong	2013	GI.5
KR107877.1	Norovirus GI.5 isolate sewage/SD0906/2013/CHN	China	Shandong	2013	GI.5
KR107876.1	Norovirus GI.5 isolate sewage/SD0905/2013/CHN	China	Shandong	2013	GI.5
KR107875.1	Norovirus GI.5 isolate sewage/SD0901/2013/CHN	China	Shandong	2013	GI.5
KR107874.1	Norovirus GI.5 isolate sewage/SD0409/2013/CHN	China	Shandong	2013	GI.5
KR107873.1	Norovirus GI.5 isolate sewage/SD0302/2013/CHN	China	Shandong	2013	GI.5
KR107872.1	Norovirus GI.5 isolate sewage/SD0209/2013/CHN	China	Shandong	2013	GI.5
KR107871.1	Norovirus GI.5 isolate sewage/SD0107/2013/CHN	China	Shandong	2013	GI.5
KR107870.1	Norovirus GI.5 isolate sewage/SD0106/2013/CHN	China	Shandong	2013	GI.5
KR107869.1	Norovirus GI.5 isolate sewage/SD0105/2013/CHN	China	Shandong	2013	GI.5

Table S1. *Cont.*

KR107868.1	Norovirus GI.5 isolate sewage/SD0104/2013/CHN	China	Shandong	2013	GI.5
KR107867.1	Norovirus GI.4 isolate sewage/SD7103/2013/CHN	China	Shandong	2013	GI.4
KR107866.1	Norovirus GI.4 isolate sewage/SD1403/2013/CHN	China	Shandong	2013	GI.4
KR107865.1	Norovirus GI.4 isolate sewage/SD7401/2013/CHN	China	Shandong	2013	GI.4
KR107864.1	Norovirus GI.4 isolate sewage/SD7108/2013/CHN	China	Shandong	2013	GI.4
KR107863.1	Norovirus GI.4 isolate sewage/SD7008/2013/CHN	China	Shandong	2013	GI.4
KR107862.1	Norovirus GI.4 isolate sewage/SD6709/2013/CHN	China	Shandong	2013	GI.4
KR107861.1	Norovirus GI.4 isolate sewage/SD6708/2013/CHN	China	Shandong	2013	GI.4
KR107860.1	Norovirus GI.4 isolate sewage/SD6607/2013/CHN	China	Shandong	2013	GI.4
KR107859.1	Norovirus GI.4 isolate sewage/SD0609/2013/CHN	China	Shandong	2013	GI.4
KR107858.1	Norovirus GI.4 isolate sewage/SD0602/2013/CHN	China	Shandong	2013	GI.4
KR107857.1	Norovirus GI.4 isolate sewage/SD0310/2013/CHN	China	Shandong	2013	GI.4
KR107856.1	Norovirus GI.3 isolate sewage/SD1405/2013/CHN	China	Shandong	2013	GI.3
KR107855.1	Norovirus GI.3 isolate sewage/SD1404/2013/CHN	China	Shandong	2013	GI.3
KR107854.1	Norovirus GI.3 isolate sewage/SD1401/2013/CHN	China	Shandong	2013	GI.3
KR107853.1	Norovirus GI.3 isolate sewage/SD1104/2013/CHN	China	Shandong	2013	GI.3
KR107852.1	Norovirus GI.3 isolate sewage/SD0509/2013/CHN	China	Shandong	2013	GI.3
KR107851.1	Norovirus GI.3 isolate sewage/SD0508/2013/CHN	China	Shandong	2013	GI.3
KR107850.1	Norovirus GI.3 isolate sewage/SD0504/2013/CHN	China	Shandong	2013	GI.3
KR107849.1	Norovirus GI.3 isolate sewage/SD0207/2013/CHN	China	Shandong	2013	GI.3
KR107848.1	Norovirus GI.3 isolate sewage/SD7605/2013/CHN	China	Shandong	2013	GI.3
KR107847.1	Norovirus GI.3 isolate sewage/SD7610/2013/CHN	China	Shandong	2013	GI.3
KR107846.1	Norovirus GI.3 isolate sewage/SD7602/2013/CHN	China	Shandong	2013	GI.3

Table S1. *Cont.*

KR107845.1	Norovirus GI.3 isolate sewage/SD7601/2013/CHN	China	Shandong	2013	GI.3
KR107844.1	Norovirus GI.3 isolate sewage/SD7509/2013/CHN	China	Shandong	2013	GI.3
KR107843.1	Norovirus GI.3 isolate sewage/SD7408/2013/CHN	China	Shandong	2013	GI.3
KR107842.1	Norovirus GI.3 isolate sewage/SD7403/2013/CHN	China	Shandong	2013	GI.3
KR107841.1	Norovirus GI.3 isolate sewage/SD7306/2013/CHN	China	Shandong	2013	GI.3
KR107840.1	Norovirus GI.3 isolate sewage/SD7303/2013/CHN	China	Shandong	2013	GI.3
KR107839.1	Norovirus GI.3 isolate sewage/SD7301/2013/CHN	China	Shandong	2013	GI.3
KR107838.1	Norovirus GI.3 isolate sewage/SD7207/2013/CHN	China	Shandong	2013	GI.3
KR107837.1	Norovirus GI.3 isolate sewage/SD7203/2013/CHN	China	Shandong	2013	GI.3
KR107836.1	Norovirus GI.3 isolate sewage/SD7110/2013/CHN	China	Shandong	2013	GI.3
KR107835.1	Norovirus GI.3 isolate sewage/SD7109/2013/CHN	China	Shandong	2013	GI.3
KR107834.1	Norovirus GI.3 isolate sewage/SD7105/2013/CHN	China	Shandong	2013	GI.3
KR107833.1	Norovirus GI.3 isolate sewage/SD7101/2013/CHN	China	Shandong	2013	GI.3
KR107832.1	Norovirus GI.3 isolate sewage/SD7007/2013/CHN	China	Shandong	2013	GI.3
KR107831.1	Norovirus GI.3 isolate sewage/SD7003/2013/CHN	China	Shandong	2013	GI.3
KR107830.1	Norovirus GI.3 isolate sewage/SD6902/2013/CHN	China	Shandong	2013	GI.3
KR107829.1	Norovirus GI.3 isolate sewage/SD6707/2013/CHN	China	Shandong	2013	GI.3
KR107828.1	Norovirus GI.3 isolate sewage/SD6706/2013/CHN	China	Shandong	2013	GI.3
KR107827.1	Norovirus GI.3 isolate sewage/SD6705/2013/CHN	China	Shandong	2013	GI.3
KR107826.1	Norovirus GI.3 isolate sewage/SD6608/2013/CHN	China	Shandong	2013	GI.3
KR107825.1	Norovirus GI.3 isolate sewage/SD6606/2013/CHN	China	Shandong	2013	GI.3
KR107824.1	Norovirus GI.3 isolate sewage/SD1510/2013/CHN	China	Shandong	2013	GI.3
KR107823.1	Norovirus GI.3 isolate sewage/SD1509/2013/CHN	China	Shandong	2013	GI.3

Table S1. *Cont.*

KR107822.1	Norovirus GI.3 isolate sewage/SD1507/2013/CHN	China	Shandong	2013	GI.3
KR107821.1	Norovirus GI.3 isolate sewage/SD1504/2013/CHN	China	Shandong	2013	GI.3
KR107820.1	Norovirus GI.3 isolate sewage/SD1503/2013/CHN	China	Shandong	2013	GI.3
KR107819.1	Norovirus GI.3 isolate sewage/SD1501/2013/CHN	China	Shandong	2013	GI.3
KR107818.1	Norovirus GI.3 isolate sewage/SD1308/2013/CHN	China	Shandong	2013	GI.3
KR107817.1	Norovirus GI.3 isolate sewage/SD1307/2013/CHN	China	Shandong	2013	GI.3
KR107816.1	Norovirus GI.3 isolate sewage/SD1210/2013/CHN	China	Shandong	2013	GI.3
KR107815.1	Norovirus GI.3 isolate sewage/SD1108/2013/CHN	China	Shandong	2013	GI.3
KR107814.1	Norovirus GI.3 isolate sewage/SD0910/2013/CHN	China	Shandong	2013	GI.3
KR107813.1	Norovirus GI.3 isolate sewage/SD0208/2013/CHN	China	Shandong	2013	GI.3
KR107812.1	Norovirus GI.3 isolate sewage/SD0203/2013/CHN	China	Shandong	2013	GI.3
KR107811.1	Norovirus GI.2 isolate sewage/SD7609/2013/CHN	China	Shandong	2013	GI.2
KR107810.1	Norovirus GI.2 isolate sewage/SD7606/2013/CHN	China	Shandong	2013	GI.2
KR107809.1	Norovirus GI.2 isolate sewage/SD7603/2013/CHN	China	Shandong	2013	GI.2
KR107808.1	Norovirus GI.2 isolate sewage/SD7508/2013/CHN	China	Shandong	2013	GI.2
KR107807.1	Norovirus GI.2 isolate sewage/SD7502/2013/CHN	China	Shandong	2013	GI.2
KR107806.1	Norovirus GI.2 isolate sewage/SD7409/2013/CHN	China	Shandong	2013	GI.2
KR107805.1	Norovirus GI.2 isolate sewage/SD7407/2013/CHN	China	Shandong	2013	GI.2
KR107804.1	Norovirus GI.2 isolate sewage/SD7404/2013/CHN	China	Shandong	2013	GI.2
KR107803.1	Norovirus GI.2 isolate sewage/SD7402/2013/CHN	China	Shandong	2013	GI.2
KR107802.1	Norovirus GI.2 isolate sewage/SD7304/2013/CHN	China	Shandong	2013	GI.2
KR107801.1	Norovirus GI.2 isolate sewage/SD7204/2013/CHN	China	Shandong	2013	GI.2
KR107800.1	Norovirus GI.2 isolate sewage/SD7010/2013/CHN	China	Shandong	2013	GI.2

Table S1. *Cont*.

KR107799.1	Norovirus GI.2 isolate sewage/SD7005/2013/CHN	China	Shandong	2013	GI.2
KR107798.1	Norovirus GI.2 isolate sewage/SD7004/2013/CHN	China	Shandong	2013	GI.2
KR107797.1	Norovirus GI.2 isolate sewage/SD7002/2013/CHN	China	Shandong	2013	GI.2
KR107796.1	Norovirus GI.2 isolate sewage/SD7001/2013/CHN	China	Shandong	2013	GI.2
KR107795.1	Norovirus GI.2 isolate sewage/SD6908/2013/CHN	China	Shandong	2013	GI.2
KR107794.1	Norovirus GI.2 isolate sewage/SD6906/2013/CHN	China	Shandong	2013	GI.2
KR107793.1	Norovirus GI.2 isolate sewage/SD6904/2013/CHN	China	Shandong	2013	GI.2
KR107792.1	Norovirus GI.2 isolate sewage/SD6810/2013/CHN	China	Shandong	2013	GI.2
KR107791.1	Norovirus GI.2 isolate sewage/SD6809/2013/CHN	China	Shandong	2013	GI.2
KR107790.1	Norovirus GI.2 isolate sewage/SD6806/2013/CHN	China	Shandong	2013	GI.2
KR107789.1	Norovirus GI.2 isolate sewage/SD6805/2013/CHN	China	Shandong	2013	GI.2
KR107788.1	Norovirus GI.2 isolate sewage/SD6804/2013/CHN	China	Shandong	2013	GI.2
KR107787.1	Norovirus GI.2 isolate sewage/SD6803/2013/CHN	China	Shandong	2013	GI.2
KR107786.1	Norovirus GI.2 isolate sewage/SD6802/2013/CHN	China	Shandong	2013	GI.2
KR107785.1	Norovirus GI.2 isolate sewage/SD6702/2013/CHN	China	Shandong	2013	GI.2
KR107784.1	Norovirus GI.2 isolate sewage/SD1505/2013/CHN	China	Shandong	2013	GI.2
KR107783.1	Norovirus GI.2 isolate sewage/SD1410/2013/CHN	China	Shandong	2013	GI.2
KR107782.1	Norovirus GI.2 isolate sewage/SD6602/2013/CHN	China	Shandong	2013	GI.2
KR107781.1	Norovirus GI.2 isolate sewage/SD1409/2013/CHN	China	Shandong	2013	GI.2
KR107780.1	Norovirus GI.2 isolate sewage/SD1407/2013/CHN	China	Shandong	2013	GI.2
KR107779.1	Norovirus GI.2 isolate sewage/SD1209/2013/CHN	China	Shandong	2013	GI.2
KR107778.1	Norovirus GI.2 isolate sewage/SD1207/2013/CHN	China	Shandong	2013	GI.2
KR107777.1	Norovirus GI.2 isolate sewage/SD1110/2013/CHN	China	Shandong	2013	GI.2

Table S1. *Cont.*

KR107776.1	Norovirus GI.2 isolate sewage/SD1109/2013/CHN	China	Shandong	2013	GI.2
KR107775.1	Norovirus GI.2 isolate sewage/SD1103/2013/CHN	China	Shandong	2013	GI.2
KR107774.1	Norovirus GI.2 isolate sewage/SD1101/2013/CHN	China	Shandong	2013	GI.2
KR107773.1	Norovirus GI.2 isolate sewage/SD0908/2013/CHN	China	Shandong	2013	GI.2
KR107772.1	Norovirus GI.2 isolate sewage/SD0903/2013/CHN	China	Shandong	2013	GI.2
KR107771.1	Norovirus GI.2 isolate sewage/SD0902/2013/CHN	China	Shandong	2013	GI.2
KR107770.1	Norovirus GI.2 isolate sewage/SD0604/2013/CHN	China	Shandong	2013	GI.2
KR107769.1	Norovirus GI.2 isolate sewage/SD0510/2013/CHN	China	Shandong	2013	GI.2
KR107768.1	Norovirus GI.2 isolate sewage/SD0503/2013/CHN	China	Shandong	2013	GI.2
KR107767.1	Norovirus GI.2 isolate sewage/SD0502/2013/CHN	China	Shandong	2013	GI.2
KR107766.1	Norovirus GI.2 isolate sewage/SD0501/2013/CHN	China	Shandong	2013	GI.2
KR107765.1	Norovirus GI.2 isolate sewage/SD0410/2013/CHN	China	Shandong	2013	GI.2
KR107764.1	Norovirus GI.2 isolate sewage/SD0408/2013/CHN	China	Shandong	2013	GI.2
KR107763.1	Norovirus GI.2 isolate sewage/SD0405/2013/CHN	China	Shandong	2013	GI.2
KR107762.1	Norovirus GI.2 isolate sewage/SD0403/2013/CHN	China	Shandong	2013	GI.2
KR107761.1	Norovirus GI.2 isolate sewage/SD0402/2013/CHN	China	Shandong	2013	GI.2
KR107760.1	Norovirus GI.2 isolate sewage/SD0102/2013/CHN	China	Shandong	2013	GI.2
KR107759.1	Norovirus GI.2 isolate sewage/SD0202/2013/CHN	China	Shandong	2013	GI.2
KR107758.1	Norovirus GI.2 isolate sewage/SD0309/2013/CHN	China	Shandong	2013	GI.2
KR107757.1	Norovirus GI.2 isolate sewage/SD0308/2013/CHN	China	Shandong	2013	GI.2
KR107756.1	Norovirus GI.2 isolate sewage/SD0307/2013/CHN	China	Shandong	2013	GI.2
KR107755.1	Norovirus GI.2 isolate sewage/SD0306/2013/CHN	China	Shandong	2013	GI.2
KR107754.1	Norovirus GI.2 isolate sewage/SD0305/2013/CHN	China	Shandong	2013	GI.2

Table S1. *Cont*.

KR107753.1	Norovirus GI.2 isolate sewage/SD0304/2013/CHN	China	Shandong	2013	GI.2
KR107752.1	Norovirus GI.2 isolate sewage/SD0303/2013/CHN	China	Shandong	2013	GI.2
KR107751.1	Norovirus GI.2 isolate sewage/SD0205/2013/CHN	China	Shandong	2013	GI.2
KR107750.1	Norovirus GI.2 isolate sewage/SD0101/2013/CHN	China	Shandong	2013	GI.2
KR107749.1	Norovirus GI.1 isolate sewage/SD6701/2013/CHN	China	Shandong	2013	GI.1
KR107748.1	Norovirus GI.1 isolate sewage/SD7305/2013/CHN	China	Shandong	2013	GI.1
KR107747.1	Norovirus GI.1 isolate sewage/SD6801/2013/CHN	China	Shandong	2013	GI.1
KR107746.1	Norovirus GI.1 isolate sewage/SD1502/2013/CHN	China	Shandong	2013	GI.1
KR107745.1	Norovirus GI.1 isolate sewage/SD1402/2013/CHN	China	Shandong	2013	GI.1
KR107744.1	Norovirus GI.1 isolate sewage/SD1310/2013/CHN	China	Shandong	2013	GI.1
KR107743.1	Norovirus GI.1 isolate sewage/SD7405/2013/CHN	China	Shandong	2013	GI.1
KR107742.1	Norovirus GI.1 isolate sewage/SD1203/2013/CHN	China	Shandong	2013	GI.1
KR107741.1	Norovirus GI.1 isolate sewage/SD0904/2013/CHN	China	Shandong	2013	GI.1
KR107740.1	Norovirus GI.1 isolate sewage/SD0608/2013/CHN	China	Shandong	2013	GI.1
KR107739.1	Norovirus GI.1 isolate sewage/SD0610/2013/CHN	China	Shandong	2013	GI.1
KR107738.1	Norovirus GI.1 isolate sewage/SD0607/2013/CHN	China	Shandong	2013	GI.1
KR107737.1	Norovirus GI.1 isolate sewage/SD0606/2013/CHN	China	Shandong	2013	GI.1
KR107736.1	Norovirus GI.1 isolate sewage/SD0605/2013/CHN	China	Shandong	2013	GI.1
KR107735.1	Norovirus GI.1 isolate sewage/SD0603/2013/CHN	China	Shandong	2013	GI.1
KR107734.1	Norovirus GI.1 isolate sewage/SD0507/2013/CHN	China	Shandong	2013	GI.1
KR107733.1	Norovirus GI.1 isolate sewage/SD0506/2013/CHN	China	Shandong	2013	GI.1
KR107732.1	Norovirus GI.1 isolate sewage/SD0505/2013/CHN	China	Shandong	2013	GI.1
KR107731.1	Norovirus GI.1 isolate sewage/SD0407/2013/CHN	China	Shandong	2013	GI.1

Table S1. *Cont*.

KR107730.1	Norovirus GI.1 isolate sewage/SD0406/2013/CHN	China	Shandong	2013	GI.1
KR107729.1	Norovirus GI.1 isolate sewage/SD0401/2013/CHN	China	Shandong	2013	GI.1
KR107728.1	Norovirus GI.1 isolate sewage/SD0201/2013/CHN	China	Shandong	2013	GI.1
KR107727.1	Norovirus GII.2 isolate sewage/GII.2/SD8106/2013/CHN	China	Shandong	2013	GII.2
KR107726.1	Norovirus GII.2 isolate sewage/GII.2/SD8610/2013/CHN	China	Shandong	2013	GII.2
KR107725.1	Norovirus GII.2 isolate sewage/GII.2/SD3010/2013/CHN	China	Shandong	2013	GII.2
KR107724.1	Norovirus GII.2 isolate sewage/GII.2/SD2601/2013/CHN	China	Shandong	2013	GII.2
KR107723.1	Norovirus GII.13 isolate sewage/GII.13/SD8903/2013/CHN	China	Shandong	2013	GII.13
KR107722.1	Norovirus GII.13 isolate sewage/GII.13/SD2705/2013/CHN	China	Shandong	2013	GII.13
KR107721.1	Norovirus GII.13 isolate sewage/GII.13/SD1906/2013/CHN	China	Shandong	2013	GII.13
KR107720.1	Norovirus GII.6 isolate sewage/GII.6/SD9007/2013/CHN	China	Shandong	2013	GII.6
KR107719.1	Norovirus GII.6 isolate sewage/GII.6/SD8810/2013/CHN	China	Shandong	2013	GII.6
KR107718.1	Norovirus GII.6 isolate sewage/GII.6/SD8708/2013/CHN	China	Shandong	2013	GII.6
KR107717.1	Norovirus GII.6 isolate sewage/GII.6/SD8707/2013/CHN	China	Shandong	2013	GII.6
KR107716.1	Norovirus GII.6 isolate sewage/GII.6/SD8705/2013/CHN	China	Shandong	2013	GII.6
KR107715.1	Norovirus GII.6 isolate sewage/GII.6/SD8601/2013/CHN	China	Shandong	2013	GII.6
KR107714.1	Norovirus GII.6 isolate sewage/GII.6/SD8310/2013/CHN	China	Shandong	2013	GII.6
KR107713.1	Norovirus GII.6 isolate sewage/GII.6/SD8301/2013/CHN	China	Shandong	2013	GII.6
KR107712.1	Norovirus GII.6 isolate sewage/GII.6/SD8006/2013/CHN	China	Shandong	2013	GII.6
KR107711.1	Norovirus GII.6 isolate sewage/GII.6/SD2810/2013/CHN	China	Shandong	2013	GII.6
KR107710.1	Norovirus GII.6 isolate sewage/GII.6/SD2803/2013/CHN	China	Shandong	2013	GII.6
KR107709.1	Norovirus GII.6 isolate sewage/GII.6/SD2701/2013/CHN	China	Shandong	2013	GII.6
KR107708.1	Norovirus GII.6 isolate sewage/GII.6/SD1706/2013/CHN	China	Shandong	2013	GII.6

Table S1. *Cont*.

KR107707.1	Norovirus GII.6 isolate sewage/GII.6/SD2802/2013/CHN	China	Shandong	2013	GII.6
KR107706.1	Norovirus GII.6 isolate sewage/GII.6/SD2706/2013/CHN	China	Shandong	2013	GII.6
KR107705.1	Norovirus GII.6 isolate sewage/GII.6/SD2704/2013/CHN	China	Shandong	2013	GII.6
KR107704.1	Norovirus GII.6 isolate sewage/GII.6/SD2610/2013/CHN	China	Shandong	2013	GII.6
KR107703.1	Norovirus GII.6 isolate sewage/GII.6/SD2607/2013/CHN	China	Shandong	2013	GII.6
KR107702.1	Norovirus GII.6 isolate sewage/GII.6/SD2606/2013/CHN	China	Shandong	2013	GII.6
KR107701.1	Norovirus GII.6 isolate sewage/GII.6/SD2605/2013/CHN	China	Shandong	2013	GII.6
KR107700.1	Norovirus GII.6 isolate sewage/GII.6/SD2602/2013/CHN	China	Shandong	2013	GII.6
KR107699.1	Norovirus GII.6 isolate sewage/GII.6/SD2404/2013/CHN	China	Shandong	2013	GII.6
KR107698.1	Norovirus GII.6 isolate sewage/GII.6/SD2103/2013/CHN	China	Shandong	2013	GII.6
KR107697.1	Norovirus GII.6 isolate sewage/GII.6/SD1710/2013/CHN	China	Shandong	2013	GII.6
KR107696.1	Norovirus GII.6 isolate sewage/GII.6/SD1607/2013/CHN	China	Shandong	2013	GII.6
KR107695.1	Norovirus GII.6 isolate sewage/GII.6/SD1608/2013/CHN	China	Shandong	2013	GII.6
KR107694.1	Norovirus GII.3 isolate sewage/GII.3/SD1605/2013/CHN	China	Shandong	2013	GII.3
KR107693.1	Norovirus GII.3 isolate sewage/GII.3/SD1602/2013/CHN	China	Shandong	2013	GII.3
KR107692.1	Norovirus GII.3 isolate sewage/GII.3/SD9006/2013/CHN	China	Shandong	2013	GII.3
KR107691.1	Norovirus GII.3 isolate sewage/GII.3/SD9004/2013/CHN	China	Shandong	2013	GII.3
KR107690.1	Norovirus GII.3 isolate sewage/GII.3/SD9003/2013/CHN	China	Shandong	2013	GII.3
KR107689.1	Norovirus GII.3 isolate sewage/GII.3/SD9002/2013/CHN	China	Shandong	2013	GII.3
KR107688.1	Norovirus GII.3 isolate sewage/GII.3/SD8803/2013/CHN	China	Shandong	2013	GII.3
KR107687.1	Norovirus GII.3 isolate sewage/GII.3/SD8703/2013/CHN	China	Shandong	2013	GII.3
KR107686.1	Norovirus GII.3 isolate sewage/GII.3/SD8606/2013/CHN	China	Shandong	2013	GII.3
KR107685.1	Norovirus GII.3 isolate sewage/GII.3/SD8503/2013/CHN	China	Shandong	2013	GII.3

Table S1. *Cont*.

KR107684.1	Norovirus GII.3 isolate sewage/GII.3/SD8406/2013/CHN	China	Shandong	2013	GII.3
KR107683.1	Norovirus GII.3 isolate sewage/GII.3/SD8405/2013/CHN	China	Shandong	2013	GII.3
KR107682.1	Norovirus GII.3 isolate sewage/GII.3/SD8404/2013/CHN	China	Shandong	2013	GII.3
KR107681.1	Norovirus GII.3 isolate sewage/GII.3/SD8403/2013/CHN	China	Shandong	2013	GII.3
KR107680.1	Norovirus GII.3 isolate sewage/GII.3/SD8309/2013/CHN	China	Shandong	2013	GII.3
KR107679.1	Norovirus GII.3 isolate sewage/GII.3/SD8308/2013/CHN	China	Shandong	2013	GII.3
KR107678.1	Norovirus GII.3 isolate sewage/GII.3/SD8306/2013/CHN	China	Shandong	2013	GII.3
KR107677.1	Norovirus GII.3 isolate sewage/GII.3/SD8206/2013/CHN	China	Shandong	2013	GII.3
KR107676.1	Norovirus GII.3 isolate sewage/GII.3/SD8203/2013/CHN	China	Shandong	2013	GII.3
KR107675.1	Norovirus GII.3 isolate sewage/GII.3/SD8109/2013/CHN	China	Shandong	2013	GII.3
KR107674.1	Norovirus GII.3 isolate sewage/GII.3/SD8108/2013/CHN	China	Shandong	2013	GII.3
KR107673.1	Norovirus GII.3 isolate sewage/GII.3/SD8107/2013/CHN	China	Shandong	2013	GII.3
KR107672.1	Norovirus GII.3 isolate sewage/GII.3/SD8105/2013/CHN	China	Shandong	2013	GII.3
KR107671.1	Norovirus GII.3 isolate sewage/GII.3/SD8101/2013/CHN	China	Shandong	2013	GII.3
KR107670.1	Norovirus GII.3 isolate sewage/GII.3/SD8004/2013/CHN	China	Shandong	2013	GII.3
KR107669.1	Norovirus GII.3 isolate sewage/GII.3/SD3008/2013/CHN	China	Shandong	2013	GII.3
KR107668.1	Norovirus GII.3 isolate sewage/GII.3/SD3007/2013/CHN	China	Shandong	2013	GII.3
KR107667.1	Norovirus GII.3 isolate sewage/GII.3/SD3006/2013/CHN	China	Shandong	2013	GII.3
KR107666.1	Norovirus GII.3 isolate sewage/GII.3/SD3005/2013/CHN	China	Shandong	2013	GII.3
KR107665.1	Norovirus GII.3 isolate sewage/GII.3/SD3002/2013/CHN	China	Shandong	2013	GII.3
KR107664.1	Norovirus GII.3 isolate sewage/GII.3/SD3001/2013/CHN	China	Shandong	2013	GII.3
KR107663.1	Norovirus GII.3 isolate sewage/GII.3/SD2910/2013/CHN	China	Shandong	2013	GII.3
KR107662.1	Norovirus GII.3 isolate sewage/GII.3/SD2909/2013/CHN	China	Shandong	2013	GII.3

Table S1. *Cont.*

KR107661.1	Norovirus GII.3 isolate sewage/GII.3/SD2908/2013/CHN	China	Shandong	2013	GII.3
KR107660.1	Norovirus GII.3 isolate sewage/GII.3/SD2905/2013/CHN	China	Shandong	2013	GII.3
KR107659.1	Norovirus GII.3 isolate sewage/GII.3/SD2903/2013/CHN	China	Shandong	2013	GII.3
KR107658.1	Norovirus GII.3 isolate sewage/GII.3/SD2902/2013/CHN	China	Shandong	2013	GII.3
KR107657.1	Norovirus GII.3 isolate sewage/GII.3/SD2809/2013/CHN	China	Shandong	2013	GII.3
KR107656.1	Norovirus GII.3 isolate sewage/GII.3/SD2808/2013/CHN	China	Shandong	2013	GII.3
KR107655.1	Norovirus GII.3 isolate sewage/GII.3/SD2709/2013/CHN	China	Shandong	2013	GII.3
KR107654.1	Norovirus GII.3 isolate sewage/GII.3/SD2708/2013/CHN	China	Shandong	2013	GII.3
KR107653.1	Norovirus GII.3 isolate sewage/GII.3/SD2707/2013/CHN	China	Shandong	2013	GII.3
KR107652.1	Norovirus GII.3 isolate sewage/GII.3/SD2702/2013/CHN	China	Shandong	2013	GII.3
KR107651.1	Norovirus GII.3 isolate sewage/GII.3/SD2603/2013/CHN	China	Shandong	2013	GII.3
KR107650.1	Norovirus GII.3 isolate sewage/GII.3/SD2509/2013/CHN	China	Shandong	2013	GII.3
KR107649.1	Norovirus GII.3 isolate sewage/GII.3/SD2407/2013/CHN	China	Shandong	2013	GII.3
KR107648.1	Norovirus GII.3 isolate sewage/GII.3/SD2406/2013/CHN	China	Shandong	2013	GII.3
KR107647.1	Norovirus GII.3 isolate sewage/GII.3/SD2110/2013/CHN	China	Shandong	2013	GII.3
KR107646.1	Norovirus GII.3 isolate sewage/GII.3/SD2109/2013/CHN	China	Shandong	2013	GII.3
KR107645.1	Norovirus GII.3 isolate sewage/GII.3/SD2108/2013/CHN	China	Shandong	2013	GII.3
KR107644.1	Norovirus GII.3 isolate sewage/GII.3/SD2107/2013/CHN	China	Shandong	2013	GII.3
KR107643.1	Norovirus GII.3 isolate sewage/GII.3/SD2105/2013/CHN	China	Shandong	2013	GII.3
KR107642.1	Norovirus GII.3 isolate sewage/GII.3/SD2104/2013/CHN	China	Shandong	2013	GII.3
KR107641.1	Norovirus GII.3 isolate sewage/GII.3/SD2102/2013/CHN	China	Shandong	2013	GII.3
KR107640.1	Norovirus GII.3 isolate sewage/GII.3/SD2101/2013/CHN	China	Shandong	2013	GII.3
KR107639.1	Norovirus GII.3 isolate sewage/GII.3/SD2010/2013/CHN	China	Shandong	2013	GII.3

Table S1. *Cont.*

KR107638.1	Norovirus GII.3 isolate sewage/GII.3/SD2009/2013/CHN	China	Shandong	2013	GII.3
KR107637.1	Norovirus GII.3 isolate sewage/GII.3/SD2008/2013/CHN	China	Shandong	2013	GII.3
KR107636.1	Norovirus GII.3 isolate sewage/GII.3/SD2006/2013/CHN	China	Shandong	2013	GII.3
KR107635.1	Norovirus GII.3 isolate sewage/GII.3/SD2005/2013/CHN	China	Shandong	2013	GII.3
KR107634.1	Norovirus GII.3 isolate sewage/GII.3/SD2004/2013/CHN	China	Shandong	2013	GII.3
KR107633.1	Norovirus GII.3 isolate sewage/GII.3/SD2003/2013/CHN	China	Shandong	2013	GII.3
KR107632.1	Norovirus GII.3 isolate sewage/GII.3/SD2002/2013/CHN	China	Shandong	2013	GII.3
KR107631.1	Norovirus GII.3 isolate sewage/GII.3/SD2001/2013/CHN	China	Shandong	2013	GII.3
KR107630.1	Norovirus GII.3 isolate sewage/GII.3/SD1910/2013/CHN	China	Shandong	2013	GII.3
KR107629.1	Norovirus GII.3 isolate sewage/GII.3/SD1908/2013/CHN	China	Shandong	2013	GII.3
KR107628.1	Norovirus GII.3 isolate sewage/GII.3/SD1907/2013/CHN	China	Shandong	2013	GII.3
KR107627.1	Norovirus GII.3 isolate sewage/GII.3/SD1905/2013/CHN	China	Shandong	2013	GII.3
KR107626.1	Norovirus GII.3 isolate sewage/GII.3/SD1904/2013/CHN	China	Shandong	2013	GII.3
KR107625.1	Norovirus GII.3 isolate sewage/GII.3/SD1902/2013/CHN	China	Shandong	2013	GII.3
KR107624.1	Norovirus GII.3 isolate sewage/GII.3/SD1901/2013/CHN	China	Shandong	2013	GII.3
KR107623.1	Norovirus GII.3 isolate sewage/GII.3/SD1810/2013/CHN	China	Shandong	2013	GII.3
KR107622.1	Norovirus GII.3 isolate sewage/GII.3/SD1808/2013/CHN	China	Shandong	2013	GII.3
KR107621.1	Norovirus GII.3 isolate sewage/GII.3/SD1807/2013/CHN	China	Shandong	2013	GII.3
KR107620.1	Norovirus GII.3 isolate sewage/GII.3/SD1805/2013/CHN	China	Shandong	2013	GII.3
KR107619.1	Norovirus GII.3 isolate sewage/GII.3/SD1804/2013/CHN	China	Shandong	2013	GII.3
KR107618.1	Norovirus GII.3 isolate sewage/GII.3/SD1801/2013/CHN	China	Shandong	2013	GII.3
KR107617.1	Norovirus GII.3 isolate sewage/GII.3/SD1709/2013/CHN	China	Shandong	2013	GII.3
KR107616.1	Norovirus GII.3 isolate sewage/GII.3/SD1708/2013/CHN	China	Shandong	2013	GII.3

Table S1. *Cont*.

KR107615.1	Norovirus GII.3 isolate sewage/GII.3/SD1704/2013/CHN	China	Shandong	2013	GII.3
KR107614.1	Norovirus GII.3 isolate sewage/GII.3/SD1703/2013/CHN	China	Shandong	2013	GII.3
KR107613.1	Norovirus GII.3 isolate sewage/GII.3/SD1702/2013/CHN	China	Shandong	2013	GII.3
KR107612.1	Norovirus GII.3 isolate sewage/GII.3/SD1701/2013/CHN	China	Shandong	2013	GII.3
KR107611.1	Norovirus GII.3 isolate sewage/GII.3/SD8508/2013/CHN	China	Shandong	2013	GII.3
KR107610.1	Norovirus GII.17 isolate sewage/GII.17/SD8507/2013/CHN	China	Shandong	2013	GII.17
KR107609.1	Norovirus GII.17 isolate sewage/GII.17/SD9001/2013/CHN	China	Shandong	2013	GII.17
KR107608.1	Norovirus GII.17 isolate sewage/GII.17/SD8905/2013/CHN	China	Shandong	2013	GII.17
KR107607.1	Norovirus GII.17 isolate sewage/GII.17/SD8807/2013/CHN	China	Shandong	2013	GII.17
KR107606.1	Norovirus GII.17 isolate sewage/GII.17/SD8802/2013/CHN	China	Shandong	2013	GII.17
KR107605.1	Norovirus GII.17 isolate sewage/GII.17/SD8801/2013/CHN	China	Shandong	2013	GII.17
KR107604.1	Norovirus GII.17 isolate sewage/GII.17/SD8704/2013/CHN	China	Shandong	2013	GII.17
KR107603.1	Norovirus GII.17 isolate sewage/GII.17/SD8609/2013/CHN	China	Shandong	2013	GII.17
KR107602.1	Norovirus GII.17 isolate sewage/GII.17/SD8607/2013/CHN	China	Shandong	2013	GII.17
KR107601.1	Norovirus GII.17 isolate sewage/GII.17/SD8603/2013/CHN	China	Shandong	2013	GII.17
KR107600.1	Norovirus GII.17 isolate sewage/GII.17/SD8501/2013/CHN	China	Shandong	2013	GII.17
KR107599.1	Norovirus GII.17 isolate sewage/GII.17/SD8303/2013/CHN	China	Shandong	2013	GII.17
KR107598.1	Norovirus GII.17 isolate sewage/GII.17/SD8005/2013/CHN	China	Shandong	2013	GII.17
KR107597.1	Norovirus GII.17 isolate sewage/GII.17/SD2807/2013/CHN	China	Shandong	2013	GII.17
KR107596.1	Norovirus GII.17 isolate sewage/GII.17/SD2806/2013/CHN	China	Shandong	2013	GII.17
KR107595.1	Norovirus GII.17 isolate sewage/GII.17/SD2805/2013/CHN	China	Shandong	2013	GII.17
KR107594.1	Norovirus GII.17 isolate sewage/GII.17/SD2801/2013/CHN	China	Shandong	2013	GII.17
KR107593.1	Norovirus GII.17 isolate sewage/GII.17/SD2609/2013/CHN	China	Shandong	2013	GII.17

Table S1. *Cont.*

KR107592.1	Norovirus GII.17 isolate sewage/GII.17/SD2504/2013/CHN	China	Shandong	2013	GII.17
KR107591.1	Norovirus GII.17 isolate sewage/GII.17/SD2408/2013/CHN	China	Shandong	2013	GII.17
KR107590.1	Norovirus GII.17 isolate sewage/GII.17/SD2403/2013/CHN	China	Shandong	2013	GII.17
KR107589.1	Norovirus GII.17 isolate sewage/GII.17/SD2401/2013/CHN	China	Shandong	2013	GII.17
KR107588.1	Norovirus GII.17 isolate sewage/GII.17/SD1809/2013/CHN	China	Shandong	2013	GII.17
KR107587.1	Norovirus GII.17 isolate sewage/GII.17/SD1802/2013/CHN	China	Shandong	2013	GII.17
KR107586.1	Norovirus GII.17 isolate sewage/GII.17/SD1606/2013/CHN	China	Shandong	2013	GII.17
KR107585.1	Norovirus GII.4 isolate sewage/GII.4/SD2904/2013/CHN	China	Shandong	2013	GII.4
KR107584.1	Norovirus GII.4 isolate sewage/GII.4/SD8906/2013/CHN	China	Shandong	2013	GII.4
KR107583.1	Norovirus GII.4 isolate sewage/GII.4/SD8702/2013/CHN	China	Shandong	2013	GII.4
KR107582.1	Norovirus GII.4 isolate sewage/GII.4/SD8410/2013/CHN	China	Shandong	2013	GII.4
KR107581.1	Norovirus GII.4 isolate sewage/GII.4/SD8409/2013/CHN	China	Shandong	2013	GII.4
KR107580.1	Norovirus GII.4 isolate sewage/GII.4/SD8408/2013/CHN	China	Shandong	2013	GII.4
KR107579.1	Norovirus GII.4 isolate sewage/GII.4/SD8407/2013/CHN	China	Shandong	2013	GII.4
KR107578.1	Norovirus GII.4 isolate sewage/GII.4/SD8401/2013/CHN	China	Shandong	2013	GII.4
KR107577.1	Norovirus GII.4 isolate sewage/GII.4/SD8307/2013/CHN	China	Shandong	2013	GII.4
KR107576.1	Norovirus GII.4 isolate sewage/GII.4/SD8305/2013/CHN	China	Shandong	2013	GII.4
KR107575.1	Norovirus GII.4 isolate sewage/GII.4/SD8304/2013/CHN	China	Shandong	2013	GII.4
KR107574.1	Norovirus GII.4 isolate sewage/GII.4/SD8205/2013/CHN	China	Shandong	2013	GII.4
KR107573.1	Norovirus GII.4 isolate sewage/GII.4/SD8204/2013/CHN	China	Shandong	2013	GII.4
KR107572.1	Norovirus GII.4 isolate sewage/GII.4/SD8110/2013/CHN	China	Shandong	2013	GII.4
KR107571.1	Norovirus GII.4 isolate sewage/GII.4/SD8104/2013/CHN	China	Shandong	2013	GII.4
KR107570.1	Norovirus GII.4 isolate sewage/GII.4/SD8008/2013/CHN	China	Shandong	2013	GII.4

Table S1. *Cont.*

KR107569.1	Norovirus GII.4 isolate sewage/GII.4/SD8001/2013/CHN	China	Shandong	2013	GII.4
KR107568.1	Norovirus GII.4 isolate sewage/GII.4/SD3009/2013/CHN	China	Shandong	2013	GII.4
KR107567.1	Norovirus GII.4 isolate sewage/GII.4/SD2906/2013/CHN	China	Shandong	2013	GII.4
KR107566.1	Norovirus GII.4 isolate sewage/GII.4/SD2804/2013/CHN	China	Shandong	2013	GII.4
KR107565.1	Norovirus GII.4 isolate sewage/GII.4/SD2703/2013/CHN	China	Shandong	2013	GII.4
KR107564.1	Norovirus GII.4 isolate sewage/GII.4/SD1603/2013/CHN	China	Shandong	2013	GII.4
KR107563.1	Norovirus GII.4 isolate sewage/GII.4/SD2106/2013/CHN	China	Shandong	2013	GII.4
KR107562.1	Norovirus GII.4 isolate sewage/GII.4/SD2007/2013/CHN	China	Shandong	2013	GII.4
KR107561.1	Norovirus GII.4 isolate sewage/GII.4/SD1903/2013/CHN	China	Shandong	2013	GII.4
KR107560.1	Norovirus GII.4 isolate sewage/GII.4/SD1705/2013/CHN	China	Shandong	2013	GII.4
KR107559.1	Norovirus GII.4 isolate sewage/GII.4/SD1609/2013/CHN	China	Shandong	2013	GII.4
KR107558.1	Norovirus GII.4 isolate sewage/GII.4/SD1601/2013/CHN	China	Shandong	2013	GII.4
KM114291.1	Norovirus Hu/GII.4/SJTUH1/CHN/2014	China	Shanghai	2014	GII.4
KP241915.1	Norovirus GII.4 isolate GII/Hu/HKG/2014/GII.4/CUHK-NS-454	China	Hong Kong	2014	GII.4
KP241914.1	Norovirus GII.4 isolate GII/Hu/HKG/2014/GII.4/CUHK-NS-453	China	Hong Kong	2014	GII.4
KP241913.1	Norovirus GII.4 isolate GII/Hu/HKG/2014/GII.4/CUHK-NS-451	China	Hong Kong	2014	GII.4
KP241912.1	Norovirus GII.4 isolate GII/Hu/HKG/2014/GII.4/CUHK-NS-448	China	Hong Kong	2014	GII.4
KP241911.1	Norovirus GII.4 isolate GII/Hu/HKG/2014/GII.4/CUHK-NS-445	China	Hong Kong	2014	GII.4
KP241910.1	Norovirus GII.4 isolate GII/Hu/HKG/2014/GII.4/CUHK-NS-444	China	Hong Kong	2014	GII.4

Table S1. *Cont*.

KP241909.1	Norovirus GII.4 isolate GII/Hu/HKG/2014/GII.4/CUHK-NS-443	China	Hong Kong	2014	GII.4
KP241908.1	Norovirus GII.4 isolate GII/Hu/HKG/2014/GII.4/CUHK-NS-440	China	Hong Kong	2014	GII.4
KP241907.1	Norovirus GII.4 isolate GII/Hu/HKG/2014/GII.4/CUHK-NS-439	China	Hong Kong	2014	GII.4
KP241906.1	Norovirus GII.4 isolate GII/Hu/HKG/2014/GII.4/CUHK-NS-437	China	Hong Kong	2014	GII.4
KP241905.1	Norovirus GII.4 isolate GII/Hu/HKG/2014/GII.4/CUHK-NS-436	China	Hong Kong	2014	GII.4
KP176412.1	Norovirus Hu/GII/HKG/2014/GII.4/CUHK-NS-435	China	Hong Kong	2014	GII.4
KP176411.1	Norovirus Hu/GII/HKG/2014/GII.4/CUHK-NS-433	China	Hong Kong	2014	GII.4
KP176410.1	Norovirus Hu/GII/HKG/2014/GII.4/CUHK-NS-431	China	Hong Kong	2014	GII.4
KP176409.1	Norovirus Hu/GII/HKG/2014/GII.4/CUHK-NS-430	China	Hong Kong	2014	GII.4
KP176408.1	Norovirus Hu/GII/HKG/2014/GII.4/CUHK-NS-429	China	Hong Kong	2014	GII.4
KP176407.1	Norovirus Hu/GII/HKG/2014/GII.4/CUHK-NS-421	China	Hong Kong	2014	GII.4
KP176406.1	Norovirus Hu/GII/HKG/2014/GII.4/CUHK-NS-420	China	Hong Kong	2014	GII.4
KP176405.1	Norovirus Hu/GII/HKG/2014/GII.4/CUHK-NS-418	China	Hong Kong	2014	GII.4
KP176404.1	Norovirus Hu/GII/HKG/2014/GII.4/CUHK-NS-417	China	Hong Kong	2014	GII.4
KP176403.1	Norovirus Hu/GII/HKG/2014/GII.4/CUHK-NS-416	China	Hong Kong	2014	GII.4

Table S1. *Cont*.

KP176402.1	Norovirus Hu/GII/HKG/2014/GII.4/CUHK-NS-415	China	Hong Kong	2014	GII.4
KP176401.1	Norovirus Hu/GII/HKG/2014/GII.4/CUHK-NS-412	China	Hong Kong	2014	GII.4
KP176400.1	Norovirus Hu/GII/HKG/2014/GII.4/CUHK-NS-407	China	Hong Kong	2014	GII.4
KP176399.1	Norovirus Hu/GII/HKG/2014/GII.4/CUHK-NS-379	China	Hong Kong	2014	GII.4
KP176398.1	Norovirus Hu/GII/HKG/2014/GII.4/CUHK-NS-376	China	Hong Kong	2014	GII.4
KP176397.1	Norovirus Hu/GII/HKG/2014/GII.4/CUHK-NS-371	China	Hong Kong	2014	GII.4
KP176396.1	Norovirus Hu/GII/HKG/2014/GII.4/CUHK-NS-356	China	Hong Kong	2014	GII.4
KP176395.1	Norovirus Hu/GII/HKG/2014/GII.4/CUHK-NS-352	China	Hong Kong	2014	GII.4
KP176394.1	Norovirus Hu/GII/HKG/2014/GII.4/CUHK-NS-346	China	Hong Kong	2014	GII.4
KP176393.1	Norovirus Hu/GII/HKG/2014/GII.4/CUHK-NS-341	China	Hong Kong	2014	GII.4
KP123606.1	Norovirus Hu/GII/HKG/2014/GII.4/CUHK-NS-426	China	Hong Kong	2014	GII.4
KP096348.1	Norovirus Hu/GII/HKG/2014/GII.4/CUHK-NS-406	China	Hong Kong	2014	GII.4
KP096347.1	Norovirus Hu/GII/HKG/2014/GII.4/CUHK-NS-404	China	Hong Kong	2014	GII.4
KP096346.1	Norovirus Hu/GII/HKG/2014/GII.4/CUHK-NS-402	China	Hong Kong	2014	GII.4
KP096345.1	Norovirus Hu/GII/HKG/2014/GII.4/CUHK-NS-399	China	Hong Kong	2014	GII.4

Table S1. *Cont.*

KP096344.1	Norovirus Hu/GII/HKG/2014/GII.4/CUHK-NS-398	China	Hong Kong	2014	GII.4
KP096343.1	Norovirus Hu/GII/HKG/2014/GII.4/CUHK-NS-397	China	Hong Kong	2014	GII.4
KP096342.1	Norovirus Hu/GII/HKG/2014/GII.4/CUHK-NS-396	China	Hong Kong	2014	GII.4
KP096341.1	Norovirus Hu/GII/HKG/2014/GII.4/CUHK-NS-395	China	Hong Kong	2014	GII.4
KP096340.1	Norovirus Hu/GII/HKG/2014/GII.4/CUHK-NS-394	China	Hong Kong	2014	GII.4
KP096339.1	Norovirus Hu/GII/HKG/2014/GII.4/CUHK-NS-393	China	Hong Kong	2014	GII.4
KP096338.1	Norovirus Hu/GII/HKG/2014/GII.4/CUHK-NS-389	China	Hong Kong	2014	GII.4
KP096337.1	Norovirus Hu/GII/HKG/2014/GII.4/CUHK-NS-387	China	Hong Kong	2014	GII.4
KP096336.1	Norovirus Hu/GII/HKG/2014/GII.4/CUHK-NS-386	China	Hong Kong	2014	GII.4
KP096335.1	Norovirus Hu/GII/HKG/2014/GII.4/CUHK-NS-385	China	Hong Kong	2014	GII.4
KP096334.1	Norovirus Hu/GII/HKG/2014/GII.4/CUHK-NS-384	China	Hong Kong	2014	GII.4
KP096333.1	Norovirus Hu/GII/HKG/2014/GII.4/CUHK-NS-383	China	Hong Kong	2014	GII.4
KP096332.1	Norovirus Hu/GII/HKG/2014/GII.4/CUHK-NS-380	China	Hong Kong	2014	GII.4
KP096331.1	Norovirus Hu/GII/HKG/2014/GII.4/CUHK-NS-378	China	Hong Kong	2014	GII.4
KP096330.1	Norovirus Hu/GII/HKG/2014/GII.4/CUHK-NS-377	China	Hong Kong	2014	GII.4

Table S1. *Cont.*

KP096329.1	Norovirus Hu/GII/HKG/2014/GII.4/CUHK-NS-374	China	Hong Kong	2014	GII.4
KP096328.1	Norovirus Hu/GII/HKG/2014/GII.4/CUHK-NS-372	China	Hong Kong	2014	GII.4
KP096327.1	Norovirus Hu/GII/HKG/2014/GII.4/CUHK-NS-368	China	Hong Kong	2014	GII.4
HM802555.1	Norovirus Hu/GII.4/Hong Kong/CU050130/2005/CHN	China	Hong Kong	2005	GII.4
HM802554.1	Norovirus Hu/GII.4/Hong Kong/CU051120/2005/CHN	China	Hong Kong	2005	GII.4
HM802553.1	Norovirus Hu/GII.4/Hong Kong/CU051013/2005/CHN	China	Hong Kong	2005	GII.4
HM802552.1	Norovirus Hu/GII.4/Hong Kong/CU050852/2005/CHN	China	Hong Kong	2005	GII.4
HM802551.1	Norovirus Hu/GII.4/Hong Kong/CU041222/2004/CHN	China	Hong Kong	2004	GII.4
HM802550.1	Norovirus Hu/GII.4/Hong Kong/CU050152/2005/CHN	China	Hong Kong	2005	GII.4
HM802549.1	Norovirus Hu/GII.4/Hong Kong/CU050136/2005/CHN	China	Hong Kong	2005	GII.4
HM802548.1	Norovirus Hu/GII.4/Hong Kong/CU050128/2005/CHN	China	Hong Kong	2005	GII.4
HM802547.1	Norovirus Hu/GII.4/Hong Kong/CU041225/2004/CHN	China	Hong Kong	2004	GII.4
HM802546.1	Norovirus Hu/GII.4/Hong Kong/CU041206/2004/CHN	China	Hong Kong	2004	GII.4
HM802545.1	Norovirus Hu/GII.4/Hong Kong/CU050431/2005/CHN	China	Hong Kong	2005	GII.4
HM802544.1	Norovirus Hu/GII.4/Hong Kong/CU051146/2005/CHN	China	Hong Kong	2005	GII.4
HM802543.1	Norovirus Hu/GII.4/Hong Kong/CU050106/2005/CHN	China	Hong Kong	2005	GII.4
HM802542.1	Norovirus Hu/GII.4/Hong Kong/CU050140/2005/CHN	China	Hong Kong	2005	GII.4
HM802541.1	Norovirus Hu/GII.4/Hong Kong/CU041213/2004/CHN	China	Hong Kong	2004	GII.4
HM802540.1	Norovirus Hu/GII.4/Hong Kong/CU050141/2005/CHN	China	Hong Kong	2005	GII.4
HM802539.1	Norovirus Hu/GII.4/Hong Kong/CU060025/2006/CHN	China	Hong Kong	2006	GII.4
HM802538.1	Norovirus Hu/GII.4/Hong Kong/CU060024/2006/CHN	China	Hong Kong	2006	GII.4

Table S1. *Cont.*

HM802537.1	Norovirus Hu/GII.4/Hong Kong/CU060014/2006/CHN	China	Hong Kong	2006	GII.4
HM802536.1	Norovirus Hu/GII.4/Hong Kong/CU060039/2006/CHN	China	Hong Kong	2006	GII.4
HM802535.1	Norovirus Hu/GII.4/Hong Kong/CU060001/2006/CHN	China	Hong Kong	2006	GII.4
HM802534.1	Norovirus Hu/GII.4/Hong Kong/CU060031/2006/CHN	China	Hong Kong	2006	GII.4
HM802533.1	Norovirus Hu/GII.4/Hong Kong/CU060037/2006/CHN	China	Hong Kong	2006	GII.4
HM802532.1	Norovirus Hu/GII.4/Hong Kong/CU060030/2006/CHN	China	Hong Kong	2006	GII.4
HM802531.1	Norovirus Hu/GII.4/Hong Kong/CU060026/2006/CHN	China	Hong Kong	2006	GII.4
HM802530.1	Norovirus Hu/GII.4/Hong Kong/CU060028/2006/CHN	China	Hong Kong	2006	GII.4
HM802529.1	Norovirus Hu/GII.4/Hong Kong/CU060009/2006/CHN	China	Hong Kong	2006	GII.4
HM802528.1	Norovirus Hu/GII.4/Hong Kong/CU060036/2006/CHN	China	Hong Kong	2006	GII.4
HM802527.1	Norovirus Hu/GII.4/Hong Kong/CU060012/2006/CHN	China	Hong Kong	2006	GII.4
HM802526.1	Norovirus Hu/GII.4/Hong Kong/CU060027/2006/CHN	China	Hong Kong	2006	GII.4
HM802525.1	Norovirus Hu/GII.4/Hong Kong/CU060011/2006/CHN	China	Hong Kong	2006	GII.4
GU991353.1	Norovirus Hu/GII/Shanghai/SH2/2008/CHN	China	Shanghai	2008	GII
KT380915.1	Norovirus GII.17 strain Hu/GII.17/142700/Shanghai/2014	China	Shanghai	2014	GII.17
KC175323.1	Norovirus Hu/GII.4/Hong Kong/CUHK3630/2012/CHN	China	Hong Kong	2012	GII.4
KJ145855.1	Norovirus Hu/GII.4-2006b/SH1242/2011	China	Shanghai	2011	GII.4-2006b
KJ145854.1	Norovirus Hu/GII.3/SH1235/2011/China	China	Shanghai	2011	GII.3
KJ145853.1	Norovirus Hu/GII.4-2010/SH0809/2011/China	China	Shanghai	2011	GII.4
KJ145852.1	Norovirus Hu/GII.6/SH0331/2011/China	China	Shanghai	2011	GII.6
KJ145851.1	Norovirus Hu/GII.7/SH0258/2011/China	China	Shanghai	2011	GII.7
KJ145850.1	Norovirus Hu/GII.2/SH0140/2011/China	China	Shanghai	2011	GII.2
KJ145849.1	Norovirus Hu/GII.2/SH0106/2011/China	China	Shanghai	2011	GII.2

Table S1. *Cont.*

KJ145848.1	Norovirus Hu/GII.4-2006b/SH0807/2010/China	China	Shanghai	2010	GII.4-2006b
KJ145847.1	Norovirus Hu/GII.4-2010/SH0632/2010/China	China	Shanghai	2010	GII.4
KT149176.1	Norovirus GII isolate Hu/Guangzhou/GZ2015-L362/CHN/2015	China	Guangdong	2015	GII
KT149175.1	Norovirus GII isolate Hu/Guangzhou/GZ2015-L343/CHN/2015	China	Guangdong	2015	GII
KT149174.1	Norovirus GII isolate Hu/Guangzhou/GZ2015-L340/CHN/2015	China	Guangdong	2015	GII
KT149173.1	Norovirus GII isolate Hu/Guangzhou/GZ2015-L339/CHN/2015	China	Guangdong	2015	GII
KT149172.1	Norovirus GII isolate Hu/Guangzhou/GZ2015-L337/CHN/2015	China	Guangdong	2015	GII
KT149171.1	Norovirus GII isolate Hu/Guangzhou/GZ2015-L325/CHN/2015	China	Guangdong	2015	GII
KT149170.1	Norovirus GII isolate Hu/Guangzhou/GZ2015-L324/CHN/2015	China	Guangdong	2015	GII
KT149169.1	Norovirus GII isolate Hu/Guangzhou/GZ2014-L313/CHN/2014	China	Guangdong	2014	GII
KT149168.1	Norovirus GII isolate Hu/Guangzhou/GZ2014-L311/CHN/2014	China	Guangdong	2014	GII
KP864144.1	Norovirus GII.17 strain GII.17/152606/Shanghai/2015/CHN	China	Shanghai	2015	GII.17
KP864143.1	Norovirus GII.17 strain GII.17/152705/Shanghai/2015/CHN	China	Shanghai	2015	GII.17
KP864142.1	Norovirus GII.17 strain GII.17/152701/Shanghai/2015/CHN	China	Shanghai	2015	GII.17
KP864141.1	Norovirus GII.17 strain GII.17/152625/Shanghai/2015/CHN	China	Shanghai	2015	GII.17
KP864140.1	Norovirus GII.17 strain GII.17/152623/Shanghai/2015/CHN	China	Shanghai	2015	GII.17
KP864139.1	Norovirus GII.17 strain GII.17/152620/Shanghai/2015/CHN	China	Shanghai	2015	GII.17
KP864138.1	Norovirus GII.17 strain GII.17/152618/Shanghai/2015/CHN	China	Shanghai	2015	GII.17

Table S1. *Cont*.

KP864137.1	Norovirus GII.17 strain GII.17/142668/Shanghai/2014/CHN	China	Shanghai	2014	GII.17
KP864136.1	Norovirus GII.17 strain GII.17/142687/Shanghai/2014/CHN	China	Shanghai	2014	GII.17
KP864135.1	Norovirus GII.17 strain GII.17/142685/Shanghai/2014/CHN	China	Shanghai	2014	GII.17
KP864134.1	Norovirus GII.17 strain GII.17/142684/Shanghai/2014/CHN	China	Shanghai	2014	GII.17
KP864133.1	Norovirus GII.17 strain GII.17/142589/Shanghai/2014/CHN	China	Shanghai	2014	GII.17
KP864132.1	Norovirus GII.17 strain GII.17/152639/Shanghai/2015/CHN	China	Shanghai	2015	GII.17
KP864131.1	Norovirus GII.17 strain GII.17/152632/Shanghai/2015/CHN	China	Shanghai	2015	GII.17
KP864130.1	Norovirus GII.17 strain GII.17/152652/Shanghai/2015/CHN	China	Shanghai	2015	GII.17
KP864129.1	Norovirus GII.17 strain GII.17/152645/Shanghai/2015/CHN	China	Shanghai	2015	GII.17
KP864128.1	Norovirus GII.17 strain GII.17/142663/Shanghai/2014/CHN	China	Shanghai	2014	GII.17
KP864127.1	Norovirus GII.17 strain GII.17/142662/Shanghai/2014/CHN	China	Shanghai	2014	GII.17
KP864126.1	Norovirus GII.17 strain GII.17/142666/Shanghai/2014/CHN	China	Shanghai	2014	GII.17
KP864125.1	Norovirus GII.4 strain GII.4/152707/Shanghai/2015/CHN	China	Shanghai	2015	GII.4
KP864124.1	Norovirus GII.4 strain GII.4/152702/Shanghai/2015/CHN	China	Shanghai	2015	GII.4
KP864123.1	Norovirus GII.4 strain GII.4/152631/Shanghai/2015/CHN	China	Shanghai	2015	GII.4
KP864122.1	Norovirus GII.4 strain GII.4/152635/Shanghai/2015/CHN	China	Shanghai	2015	GII.4
KP864121.1	Norovirus GII.4 strain GII.4/152664/Shanghai/2015/CHN	China	Shanghai	2015	GII.4
KP864120.1	Norovirus GII.4 strain GII.4/152643/Shanghai/2015/CHN	China	Shanghai	2015	GII.4
KP864119.1	Norovirus GII.4 strain GII.4/142669/Shanghai/2014/CHN	China	Shanghai	2014	GII.4
KP864118.1	Norovirus GII.4 strain GII.4/142651/Shanghai/2014/CHN	China	Shanghai	2014	GII.4
KP864117.1	Norovirus GII.4 strain GII.4/142690/Shanghai/2014/CHN	China	Shanghai	2014	GII.4
KP864116.1	Norovirus GII.4 strain GII.4/142674/Shanghai/2014/CHN	China	Shanghai	2014	GII.4
KP864115.1	Norovirus GII.4 strain GII.4/142683/Shanghai/2014/CHN	China	Shanghai	2014	GII.4

Table S1. *Cont.*

KP864114.1	Norovirus GII.4 strain GII.4/142584/Shanghai/2014/CHN	China	Shanghai	2014	GII.4
KP864113.1	Norovirus GII.4 strain GII.4/142597/Shanghai/2014/CHN	China	Shanghai	2014	GII.4
KP864112.1	Norovirus GII.4 strain GII.4/141967/Shanghai/2014/CHN	China	Shanghai	2014	GII.4
KP864111.1	Norovirus GII.4 strain GII.4/141784/Shanghai/2014/CHN	China	Shanghai	2014	GII.4
KP864110.1	Norovirus GII.13 strain GII.13/152709/Shanghai/2015/CHN	China	Shanghai	2015	GII.13
KP864109.1	Norovirus GII.6 strain GII.6/142665/Shanghai/2014/CHN	China	Shanghai	2014	GII.6
KP864108.1	Norovirus GII.2 strain GII.2/142558/Shanghai/2014/CHN	China	Shanghai	2014	GII.2
KP864107.1	Norovirus GII.4 strain GII.4/152624/Shanghai/2015/CHN	China	Shanghai	2015	GII.4
KP864106.1	Norovirus GII.4 strain GII.4/142681/Shanghai/2014/CHN	China	Shanghai	2014	GII.4
KP864105.1	Norovirus GII.4 strain GII.4/142696/Shanghai/2014/CHN	China	Shanghai	2014	GII.4
JX427601.1	Norovirus Hu/GII.4/NV396/China/2010	China	Guizhou	2010	GII.4
JX427600.1	Norovirus Hu/GII.4/NV384/China/2010	China	Guizhou	2010	GII.4
JX427599.1	Norovirus Hu/GII.4/NV387/China/2010	China	Guizhou	2010	GII.4
JX427598.1	Norovirus Hu/GII.4/NV382/China/2010	China	Guizhou	2010	GII.4
JX427597.1	Norovirus Hu/GII.4/NV378/China/2010	China	Guizhou	2010	GII.4
JX427596.1	Norovirus Hu/GII.4/NV360/China/2010	China	Guizhou	2010	GII.4
JX427595.1	Norovirus Hu/GII.4/NV327/China/2010	China	Guizhou	2010	GII.4
JX427594.1	Norovirus Hu/GII.4/NV354/China/2010	China	Guizhou	2010	GII.4
JX427593.1	Norovirus Hu/GII.4/NV350/China/2010	China	Guizhou	2010	GII.4
JX427592.1	Norovirus Hu/GII.4/NV320/China/2010	China	Guizhou	2010	GII.4
JX427591.1	Norovirus Hu/GII.4/NV311/China/2010	China	Guizhou	2010	GII.4
JX427590.1	Norovirus Hu/GII.4/NV307/China/2010	China	Guizhou	2010	GII.4
JX427589.1	Norovirus Hu/GII.4/NV295/China/2010	China	Guizhou	2010	GII.4

Table S1. *Cont*.

JX427588.1	Norovirus Hu/GII.4/NV281/China/2010	China	Guizhou	2010	GII.4
JX427587.1	Norovirus Hu/GII.4/NV277/China/2010	China	Guizhou	2010	GII.4
JX427586.1	Norovirus Hu/GII.4/NV216/China/2010	China	Guizhou	2010	GII.4
JX427585.1	Norovirus Hu/GII.4/NV211/China/2010	China	Guizhou	2010	GII.4
JX427584.1	Norovirus Hu/GII.4/NV207/China/2010	China	Guizhou	2010	GII.4
JX427583.1	Norovirus Hu/GII.4/NV204/China/2010	China	Guizhou	2010	GII.4
JX427582.1	Norovirus Hu/GII.4/N141/China/2010	China	Guizhou	2010	GII.4
JX427581.1	Norovirus Hu/GII.4/NV100/China/2010	China	Guizhou	2010	GII.4
JX427580.1	Norovirus Hu/GII.4/NV86/China/2010	China	Guizhou	2010	GII.4
JX427579.1	Norovirus Hu/GII.4/NV85/China/2011	China	Guizhou	2011	GII.4
JX427578.1	Norovirus Hu/GII.4/NV84/China/2011	China	Guizhou	2011	GII.4
JX427577.1	Norovirus Hu/GII.4/NV79/China/2011	China	Guizhou	2011	GII.4
JX427576.1	Norovirus Hu/GII.4/NV65/China/2010	China	Guizhou	2010	GII.4
JX427575.1	Norovirus Hu/GII.4/NV64/China/2010	China	Guizhou	2010	GII.4
JX427574.1	Norovirus Hu/GII.4/NV62/China/2010	China	Guizhou	2010	GII.4
JX427573.1	Norovirus Hu/GII.4/NV60/China/2010	China	Guizhou	2010	GII.4
JX427572.1	Norovirus Hu/GII.4/NV58/China/2010	China	Guizhou	2010	GII.4
JX427571.1	Norovirus Hu/GII.4/NV56/China/2010	China	Guizhou	2010	GII.4
JX427570.1	Norovirus Hu/GII.4/NV54/China/2010	China	Guizhou	2010	GII.4
JX427569.1	Norovirus Hu/GII.4/NV53/China/2010	China	Guizhou	2010	GII.4
JX427568.1	Norovirus Hu/GII.4/NV51/China/2010	China	Guizhou	2010	GII.4
JX427567.1	Norovirus Hu/GII.4/NV43/China/2010	China	Guizhou	2010	GII.4
JX427566.1	Norovirus Hu/GII.4/NV37/China/2010	China	Guizhou	2010	GII.4

Table S1. *Cont.*

JX427565.1	Norovirus Hu/GII.4/NV27/China/2010	China	Guizhou	2010	GII.4
JX427564.1	Norovirus Hu/GII.4/NV19/China/2010	China	Guizhou	2010	GII.4
JX427563.1	Norovirus Hu/GII.4/NV1/China/2010	China	Guizhou	2010	GII.4
KP864104.1	Norovirus GII.17 strain GII.17/142661/Shanghai/2014/CHN	China	Shanghai	2014	GII.17
KP864103.1	Norovirus GII.17 strain GII.17/142700/Shanghai/2014/CHN	China	Shanghai	2014	GII.17
KP864102.1	Norovirus GII.17 strain GII.17/152642/Shanghai/2015/CHN	China	Shanghai	2015	GII.17
KT033905.1	Norovirus Hu/GII.4/SPHC1183/2012/CHN	China	Shanghai	2012	GII.4
KT033904.1	Norovirus Hu/GII.4/SPHC1134/2012/CHN	China	Shanghai	2012	GII.4
KT033903.1	Norovirus Hu/GII.4/SPHC2715/2012/CHN	China	Shanghai	2012	GII.4
KT033902.1	Norovirus Hu/GII.4/SPHC2048/2012/CHN	China	Shanghai	2012	GII.4
JX984953.1	Norovirus Hu/GII.6/GZ2010-L96/Guangzhou/CHN/2011	China	Guangdong	2011	GII.6
JX984952.1	Norovirus Hu/GII.4/GZ2010-L91/Guangzhou/CHN/2011	China	Guangdong	2011	GII.4
JX984951.1	Norovirus Hu/GII.4/GZ2010-L88/Guangzhou/CHN/2011	China	Guangdong	2011	GII.4
JX984950.1	Norovirus Hu/GII.4/GZ2010-L87/Guangzhou/CHN/2011	China	Guangdong	2011	GII.4
JX984949.1	Norovirus Hu/GII.6/GZ2010-L72/Guangzhou/CHN/2010	China	Guangdong	2010	GII.6
JX984948.1	Norovirus Hu/GII.3/GZ2010-L63/Guangzhou/CHN/2010	China	Guangdong	2010	GII.3
JX984947.1	Norovirus Hu/GII.4/GZ2010-L32/Guangzhou/CHN/2010	China	Guangdong	2010	GII.4
JX984946.1	Norovirus Hu/GII.4/GZ2010-L26/Guangzhou/CHN/2010	China	Guangdong	2010	GII.4
JX984945.1	Norovirus Hu/GII.6/GZ2010-L1/Guangzhou/CHN/2010	China	Guangdong	2010	GII.6
AB924677.1	Norovirus Hu/GII.4/Yunnan/31-YN/2013/CHN	China	Yunnan	2013	GII.4
AB924676.1	Norovirus Hu/GII.4/Yunnan/29-YN/2013/CHN	China	Yunnan	2013	GII.4
AB924675.1	Norovirus Hu/GII.4/Yunnan/28-YN/2013/CHN	China	Yunnan	2013	GII.4
AB924674.1	Norovirus Hu/GII.4/Yunnan/26-YN/2013/CHN	China	Yunnan	2013	GII.4

Table S1. *Cont.*

AB924673.1	Norovirus Hu/GII.4/Yunnan/25-YN/2013/CHN	China	Yunnan	2013	GII.4
AB924672.1	Norovirus Hu/GII.4/Yunnan/23-YN/2013/CHN	China	Yunnan	2013	GII.4
AB924671.1	Norovirus Hu/GII.3/Yunnan/22-YN/2013/CHN	China	Yunnan	2013	GII.3
AB924670.1	Norovirus Hu/GII.4/Yunnan/21-YN/2013/CHN	China	Yunnan	2013	GII.4
AB924669.1	Norovirus Hu/GII.3/Yunnan/20-YN/2013/CHN	China	Yunnan	2013	GII.3
AB924668.1	Norovirus Hu/GII.4/Yunnan/19-YN/2013/CHN	China	Yunnan	2013	GII.4
AB924667.1	Norovirus Hu/GII.4/Yunnan/18-YN/2013/CHN	China	Yunnan	2013	GII.4
AB924666.1	Norovirus Hu/GII.4/Yunnan/17-YN/2013/CHN	China	Yunnan	2013	GII.4
KM514075.2	Norovirus GII/Hu/HKG/2014/GII.4/CUHK-NS-332	China	Hong Kong	2014	GII.4
KP902590.1	Norovirus Hu/GII.17/HKG/2015/CUHK-NS-575	China	Hong Kong	2015	GII.17
KP902589.1	Norovirus Hu/GII.17/HKG/2015/CUHK-NS-574	China	Hong Kong	2015	GII.17
KP902588.1	Norovirus Hu/GII.17/HKG/2015/CUHK-NS-565	China	Hong Kong	2015	GII.17
KP902587.1	Norovirus Hu/GII.17/HKG/2015/CUHK-NS-556	China	Hong Kong	2015	GII.17
KP902586.1	Norovirus Hu/GII.17/HKG/2015/CUHK-NS-549	China	Hong Kong	2015	GII.17
KP902585.1	Norovirus Hu/GII.17/HKG/2015/CUHK-NS-528	China	Hong Kong	2015	GII.17
KP902584.1	Norovirus Hu/GII.17/HKG/2015/CUHK-NS-521	China	Hong Kong	2015	GII.17
KP902583.1	Norovirus Hu/GII.17/HKG/2015/CUHK-NS-520	China	Hong Kong	2015	GII.17
KP902582.1	Norovirus Hu/GII.17/HKG/2015/CUHK-NS-517	China	Hong Kong	2015	GII.17

Table S1. *Cont.*

KP902581.1	Norovirus Hu/GII.17/HKG/2015/CUHK-NS-514	China	Hong Kong	2015	GII.17
KP902580.1	Norovirus Hu/GII.17/HKG/2015/CUHK-NS-512	China	Hong Kong	2015	GII.17
KP902579.1	Norovirus Hu/GII.17/HKG/2015/CUHK-NS-506	China	Hong Kong	2015	GII.17
KP902578.1	Norovirus Hu/GII.17/HKG/2014/CUHK-NS-503	China	Hong Kong	2014	GII.17
KP902577.1	Norovirus Hu/GII.17/HKG/2014/CUHK-NS-502	China	Hong Kong	2014	GII.17
KP902576.1	Norovirus Hu/GII.17/HKG/2014/CUHK-NS-500	China	Hong Kong	2014	GII.17
KP902575.1	Norovirus Hu/GII.17/HKG/2014/CUHK-NS-493	China	Hong Kong	2014	GII.17
KP902574.1	Norovirus Hu/GII.17/HKG/2014/CUHK-NS-492	China	Hong Kong	2014	GII.17
KP902573.1	Norovirus Hu/GII.17/HKG/2014/CUHK-NS-483	China	Hong Kong	2014	GII.17
KP902572.1	Norovirus Hu/GII.17/HKG/2014/CUHK-NS-482	China	Hong Kong	2014	GII.17
KP902571.1	Norovirus Hu/GII.17/HKG/2014/CUHK-NS-480	China	Hong Kong	2014	GII.17
KP902570.1	Norovirus Hu/GII.17/HKG/2014/CUHK-NS-463	China	Hong Kong	2014	GII.17
KP902569.1	Norovirus Hu/GII.17/HKG/2014/CUHK-NS-456	China	Hong Kong	2014	GII.17
KP902568.1	Norovirus Hu/GII.17/HKG/2014/CUHK-NS-455	China	Hong Kong	2014	GII.17
KP902567.1	Norovirus Hu/GII.17/HKG/2014/CUHK-NS-438	China	Hong Kong	2014	GII.17

Table S1. *Cont.*

KP902566.1	Norovirus Hu/GII.17/HKG/2014/CUHK-NS-405	China	Hong Kong	2014	GII.17
KP902565.1	Norovirus Hu/GII.17/HKG/2014/CUHK-NS-360	China	Hong Kong	2014	GII.17
KP902564.1	Norovirus Hu/GII.17/HKG/2014/CUHK-NS-276	China	Hong Kong	2014	GII.17
KP902563.1	Norovirus Hu/GII.17/HKG/2014/CUHK-NS-258	China	Hong Kong	2014	GII.17
KJ499445.1	Norovirus GII.3 strain GII/Hu/HKG/2014/GII.3/CUHK-NS-232	China	Hong Kong	2014	GII.3
KJ499444.1	Norovirus GII.3 strain GII/Hu/HKG/2014/GII.3/CUHK-NS-227	China	Hong Kong	2014	GII.3
KJ499443.1	Norovirus GII.3 strain GII/Hu/HKG/2013/GII.3/CUHK-NS-218	China	Hong Kong	2013	GII.3
KJ499442.1	Norovirus GII.3 strain GII/Hu/HKG/2013/GII.3/CUHK-NS-201	China	Hong Kong	2013	GII.3
KJ499441.1	Norovirus GII.3 strain GII/Hu/HKG/2013/GII.3/CUHK-NS-193	China	Hong Kong	2013	GII.3
JX074065.1	Norovirus Hu/GI.4/GuizhouCDC189/2010/CHN	China	Guizhou	2010	GI.4
KM514079.1	Norovirus GII/Hu/HKG/2014/GII.4/CUHK-NS-336	China	Hong Kong	2014	GII.4
KM514078.1	Norovirus GII/Hu/HKG/2014/GII.4/CUHK-NS-335	China	Hong Kong	2014	GII.4
KM514077.1	Norovirus GII/Hu/HKG/2014/GII.4/CUHK-NS-334	China	Hong Kong	2014	GII.4
KM514076.1	Norovirus GII/Hu/HKG/2014/GII.4/CUHK-NS-333	China	Hong Kong	2014	GII.4
KM514074.1	Norovirus GII/Hu/HKG/2014/GII.4/CUHK-NS-331	China	Hong Kong	2014	GII.4

Table S1. *Cont*.

KM514073.1	Norovirus GII/Hu/HKG/2014/GII.4/CUHK-NS-330	China	Hong Kong	2014	GII.4
KM514072.1	Norovirus GII/Hu/HKG/2014/GII.4/CUHK-NS-329	China	Hong Kong	2014	GII.4
KM514071.1	Norovirus GII/Hu/HKG/2014/GII.4/CUHK-NS-328	China	Hong Kong	2014	GII.4
KM514070.1	Norovirus GII/Hu/HKG/2014/GII.4/CUHK-NS-327	China	Hong Kong	2014	GII.4
KM514069.1	Norovirus GII/Hu/HKG/2014/GII.4/CUHK-NS-324	China	Hong Kong	2014	GII.4
KM514068.1	Norovirus GII/Hu/HKG/2014/GII.4/CUHK-NS-323	China	Hong Kong	2014	GII.4
KM514067.1	Norovirus GII/Hu/HKG/2014/GII.4/CUHK-NS-322	China	Hong Kong	2014	GII.4
KM514066.1	Norovirus GII/Hu/HKG/2014/GII.4/CUHK-NS-321	China	Hong Kong	2014	GII.4
KM514065.1	Norovirus GII/Hu/HKG/2014/GII.4/CUHK-NS-320	China	Hong Kong	2014	GII.4
KM514064.1	Norovirus GII/Hu/HKG/2014/GII.4/CUHK-NS-319	China	Hong Kong	2014	GII.4
KM514063.1	Norovirus GII/Hu/HKG/2014/GII.4/CUHK-NS-318	China	Hong Kong	2014	GII.4
KM514062.1	Norovirus GII/Hu/HKG/2014/GII.4/CUHK-NS-317	China	Hong Kong	2014	GII.4
KM514061.1	Norovirus GII/Hu/HKG/2014/GII.4/CUHK-NS-316	China	Hong Kong	2014	GII.4
KM514060.1	Norovirus GII/Hu/HKG/2014/GII.4/CUHK-NS-315	China	Hong Kong	2014	GII.4
KM514059.1	Norovirus GII/Hu/HKG/2014/GII.4/CUHK-NS-313	China	Hong Kong	2014	GII.4

Table S1. *Cont.*

KM514058.1	Norovirus GII/Hu/HKG/2014/GII.4/CUHK-NS-312	China	Hong Kong	2014	GII.4
KM514057.1	Norovirus GII/Hu/HKG/2014/GII.4/CUHK-NS-311	China	Hong Kong	2014	GII.4
KM396961.1	Norovirus GII/Hu/HKG/2014/GII.4/CUHK-NS-339	China	Hong Kong	2014	GII.4
GU228570.1	Norovirus Hu/GII.4/Hong Kong/CUIF053/2006/CHN	China	Hong Kong	2006	GII.4
KT780393.1	Norovirus Hu/GII.4/CUHK-NS-663/HKG/2015	China	Hong Kong	2015	GII.4
KT780392.1	Norovirus Hu/GII.4/CUHK-NS-661/HKG/2015	China	Hong Kong	2015	GII.4
KT780391.1	Norovirus Hu/GII.4/CUHK-NS-660/HKG/2015	China	Hong Kong	2015	GII.4
KT780390.1	Norovirus Hu/GII.4/CUHK-NS-652/HKG/2015	China	Hong Kong	2015	GII.4
KT780389.1	Norovirus Hu/GII.4/CUHK-NS-651/HKG/2015	China	Hong Kong	2015	GII.4
KT780388.1	Norovirus Hu/GII.4/CUHK-NS-618/HKG/2015	China	Hong Kong	2015	GII.4
KT780387.1	Norovirus Hu/GII.4/CUHK-NS-598/HKG/2015	China	Hong Kong	2015	GII.4
KT780386.1	Norovirus Hu/GII.4/CUHK-NS-584/HKG/2015	China	Hong Kong	2015	GII.4
KT780385.1	Norovirus Hu/GII.4/CUHK-NS-572/HKG/2015	China	Hong Kong	2015	GII.4
KT780384.1	Norovirus Hu/GII.4/CUHK-NS-566/HKG/2015	China	Hong Kong	2015	GII.4
KT780383.1	Norovirus Hu/GII.4/CUHK-NS-559/HKG/2015	China	Hong Kong	2015	GII.4
KT780382.1	Norovirus Hu/GII.4/CUHK-NS-555/HKG/2015	China	Hong Kong	2015	GII.4
KT780381.1	Norovirus Hu/GII.4/CUHK-NS-554/HKG/2015	China	Hong Kong	2015	GII.4
KT780380.1	Norovirus Hu/GII.4/CUHK-NS-544/HKG/2015	China	Hong Kong	2015	GII.4
KT780379.1	Norovirus Hu/GII.4/CUHK-NS-542/HKG/2015	China	Hong Kong	2015	GII.4
KT780378.1	Norovirus Hu/GII.4/CUHK-NS-530/HKG/2015	China	Hong Kong	2015	GII.4
KT780377.1	Norovirus Hu/GII.4/CUHK-NS-529/HKG/2015	China	Hong Kong	2015	GII.4

Table S1. *Cont.*

KT780376.1	Norovirus Hu/GII.4/CUHK-NS-526/HKG/2015	China	Hong Kong	2015	GII.4
KT780375.1	Norovirus Hu/GII.4/CUHK-NS-525/HKG/2015	China	Hong Kong	2015	GII.4
KT780374.1	Norovirus Hu/GII.4/CUHK-NS-524/HKG/2015	China	Hong Kong	2015	GII.4
KT780373.1	Norovirus Hu/GII.4/CUHK-NS-516/HKG/2015	China	Hong Kong	2015	GII.4
KT780372.1	Norovirus Hu/GII.4/CUHK-NS-515/HKG/2015	China	Hong Kong	2015	GII.4
KT780371.1	Norovirus Hu/GII.4/CUHK-NS-508/HKG/2015	China	Hong Kong	2015	GII.4
KT315719.1	Norovirus Hu/GII.17/CUHK-NS-671/HKG/2015	China	Hong Kong	2015	GII.17
KT315718.1	Norovirus Hu/GII.17/CUHK-NS-670/HKG/2015	China	Hong Kong	2015	GII.17
KT315717.1	Norovirus Hu/GII.17/CUHK-NS-667/HKG/2015	China	Hong Kong	2015	GII.17
KT315716.1	Norovirus Hu/GII.17/CUHK-NS-662/HKG/2015	China	Hong Kong	2015	GII.17
KT315715.1	Norovirus Hu/GII.17/CUHK-NS-659/HKG/2015	China	Hong Kong	2015	GII.17
KT315714.1	Norovirus Hu/GII.17/CUHK-NS-658/HKG/2015	China	Hong Kong	2015	GII.17
KT315713.1	Norovirus Hu/GII.17/CUHK-NS-657/HKG/2015	China	Hong Kong	2015	GII.17
KT315712.1	Norovirus Hu/GII.17/CUHK-NS-656/HKG/2015	China	Hong Kong	2015	GII.17
KT315711.1	Norovirus Hu/GII.17/CUHK-NS-655/HKG/2015	China	Hong Kong	2015	GII.17
KT315710.1	Norovirus Hu/GII.17/CUHK-NS-653/HKG/2015	China	Hong Kong	2015	GII.17
KT315709.1	Norovirus Hu/GII.17/CUHK-NS-650/HKG/2015	China	Hong Kong	2015	GII.17
KT315708.1	Norovirus Hu/GII.17/CUHK-NS-649/HKG/2015	China	Hong Kong	2015	GII.17
KT315707.1	Norovirus Hu/GII.17/CUHK-NS-648/HKG/2015	China	Hong Kong	2015	GII.17
KT315706.1	Norovirus Hu/GII.17/CUHK-NS-647/HKG/2015	China	Hong Kong	2015	GII.17
KT315705.1	Norovirus Hu/GII.17/CUHK-NS-643/HKG/2015	China	Hong Kong	2015	GII.17
KT315704.1	Norovirus Hu/GII.17/CUHK-NS-641/HKG/2015	China	Hong Kong	2015	GII.17
KT315703.1	Norovirus Hu/GII.17/CUHK-NS-639/HKG/2015	China	Hong Kong	2015	GII.17

Table S1. *Cont*.

KT315702.1	Norovirus Hu/GII.17/CUHK-NS-637/HKG/2015	China	Hong Kong	2015	GII.17
KT315701.1	Norovirus Hu/GII.17/CUHK-NS-636/HKG/2015	China	Hong Kong	2015	GII.17
KT315700.1	Norovirus Hu/GII.17/CUHK-NS-634/HKG/2015	China	Hong Kong	2015	GII.17
KT315699.1	Norovirus Hu/GII.17/CUHK-NS-629/HKG/2015	China	Hong Kong	2015	GII.17
KT315698.1	Norovirus Hu/GII.17/CUHK-NS-627/HKG/2015	China	Hong Kong	2015	GII.17
KT315697.1	Norovirus Hu/GII.17/CUHK-NS-619/HKG/2015	China	Hong Kong	2015	GII.17
KT315696.1	Norovirus Hu/GII.17/CUHK-NS-616/HKG/2015	China	Hong Kong	2015	GII.17
KT315695.1	Norovirus Hu/GII.17/CUHK-NS-612/HKG/2015	China	Hong Kong	2015	GII.17
KT315694.1	Norovirus Hu/GII.17/CUHK-NS-611/HKG/2015	China	Hong Kong	2015	GII.17
KT315693.1	Norovirus Hu/GII.17/CUHK-NS-606/HKG/2015	China	Hong Kong	2015	GII.17
KT315692.1	Norovirus Hu/GII.17/CUHK-NS-604/HKG/2015	China	Hong Kong	2015	GII.17
KT315691.1	Norovirus Hu/GII.17/CUHK-NS-603/HKG/2015	China	Hong Kong	2015	GII.17
KT315690.1	Norovirus Hu/GII.17/CUHK-NS-602/HKG/2015	China	Hong Kong	2015	GII.17
KT315689.1	Norovirus Hu/GII.17/CUHK-NS-600/HKG/2015	China	Hong Kong	2015	GII.17
KT315688.1	Norovirus Hu/GII.17/CUHK-NS-599/HKG/2015	China	Hong Kong	2015	GII.17
KT315687.1	Norovirus Hu/GII.17/CUHK-NS-593/HKG/2015	China	Hong Kong	2015	GII.17
KT315686.1	Norovirus Hu/GII.17/CUHK-NS-592/HKG/2015	China	Hong Kong	2015	GII.17
KT315685.1	Norovirus Hu/GII.17/CUHK-NS-589/HKG/2015	China	Hong Kong	2015	GII.17
KT315684.1	Norovirus Hu/GII.17/CUHK-NS-586/HKG/2015	China	Hong Kong	2015	GII.17
KT315683.1	Norovirus Hu/GII.17/CUHK-NS-582/HKG/2015	China	Hong Kong	2015	GII.17
KT315682.1	Norovirus Hu/GII.17/CUHK-NS-579/HKG/2015	China	Hong Kong	2015	GII.17
KT315681.1	Norovirus Hu/GII.17/CUHK-NS-570/HKG/2015	China	Hong Kong	2015	GII.17
KT315680.1	Norovirus Hu/GII.17/CUHK-NS-560/HKG/2015	China	Hong Kong	2015	GII.17

Table S1. *Cont.*

KT315679.1	Norovirus Hu/GII.17/CUHK-NS-539/HKG/2015	China	Hong Kong	2015	GII.17
KT315678.1	Norovirus Hu/GII.17/CUHK-NS-537/HKG/2015	China	Hong Kong	2015	GII.17
KT315677.1	Norovirus Hu/GII.17/CUHK-NS-534/HKG/2015	China	Hong Kong	2015	GII.17
KT315676.1	Norovirus Hu/GII.17/CUHK-NS-533/HKG/2015	China	Hong Kong	2015	GII.17
KT315675.1	Norovirus Hu/GII.17/CUHK-NS-523/HKG/2015	China	Hong Kong	2015	GII.17
KT315674.1	Norovirus Hu/GII.17/CUHK-NS-522/HKG/2015	China	Hong Kong	2015	GII.17
KT315673.1	Norovirus Hu/GII.17/CUHK-NS-486/HKG/2014	China	Hong Kong	2014	GII.17
KT315672.1	Norovirus Hu/GII.17/CUHK-NS-478/HKG/2014	China	Hong Kong	2014	GII.17
KT315671.1	Norovirus Hu/GII.17/CUHK-NS-476/HKG/2014	China	Hong Kong	2014	GII.17
KT315670.1	Norovirus Hu/GII.17/CUHK-NS-475/HKG/2014	China	Hong Kong	2014	GII.17
KT315669.1	Norovirus Hu/GII.17/CUHK-NS-472/HKG/2014	China	Hong Kong	2014	GII.17
KT315668.1	Norovirus Hu/GII.17/CUHK-NS-469/HKG/2014	China	Hong Kong	2014	GII.17
KP698931.1	Norovirus GII.17 isolate GII/Hu/HKG/2015	China	Hong Kong	2015	GII.17
KP698930.1	Norovirus GII.17 isolate GII/Hu/HKG/2015	China	Hong Kong	2015	GII.17
KP698929.1	Norovirus GII.17 isolate GII/Hu/HKG/2014	China	Hong Kong	2014	GII.17
KP698928.1	Norovirus GII.17 isolate GII/Hu/HKG/2014	China	Hong Kong	2014	GII.17
KP698927.1	Norovirus GII.4 isolate GII/Hu/HKG/2014/GII.4/CUHK-NS-505	China	Hong Kong	2014	GII.4
KP698926.1	Norovirus GII.4 isolate GII/Hu/HKG/2014/GII.4/CUHK-NS-490	China	Hong Kong	2014	GII.4
KP698925.1	Norovirus GII.4 isolate GII/Hu/HKG/2014/GII.4/CUHK-NS-489	China	Hong Kong	2014	GII.4
KP698924.1	Norovirus GII.4 isolate GII/Hu/HKG/2014/GII.4/CUHK-NS-485	China	Hong Kong	2014	GII.4

Table S1. *Cont.*

KP698923.1	Norovirus GII.4 isolate GII/Hu/HKG/2014/GII.4/CUHK-NS-474	China	Hong Kong	2014	GII.4
KR921942.1	Norovirus Hu/GII.21/CUHK-NS-626/HKG/2015	China	Hong Kong	2015	GII.21
KR921941.1	Norovirus Hu/GII.21/CUHK-NS-620/HKG/2015	China	Hong Kong	2015	GII.21
KR921940.1	Norovirus Hu/GII.21/CUHK-NS-609/HKG/2015	China	Hong Kong	2015	GII.21
KR921939.1	Norovirus Hu/GII.21/CUHK-NS-601/HKG/2015	China	Hong Kong	2015	GII.21
KR921938.1	Norovirus Hu/GII.21/CUHK-NS-591/HKG/2015	China	Hong Kong	2015	GII.21
KR921937.1	Norovirus Hu/GII.21/CUHK-NS-293/HKG/2014	China	Hong Kong	2014	GII.21
KR921936.1	Norovirus Hu/GII.21/CUHK-NS-291/HKG/2014	China	Hong Kong	2014	GII.21
KR921935.1	Norovirus Hu/GII.21/CUHK-NS-290/HKG/2014	China	Hong Kong	2014	GII.21
JX989075.1	Norovirus Hu/GII.6/GZ2010-L96/Guangzhou/CHN/2011	China	Guangdong	2011	GII.6
JX989074.1	Norovirus Hu/GII.4/GZ2010-L87/Guangzhou/CHN/2011	China	Guangdong	2011	GII.4
KT970377.1	Norovirus GII isolate Hu/Guangzhou/GZ2015-L362/CHN/2015	China	Guangdong	2015	GII
KT970376.1	Norovirus GII isolate Hu/Guangzhou/GZ2015-L343/CHN/2015	China	Guangdong	2015	GII
KT970375.1	Norovirus GII isolate Hu/Guangzhou/GZ2015-L340/CHN/2015	China	Guangdong	2015	GII
KT970374.1	Norovirus GII isolate Hu/Guangzhou/GZ2015-L339/CHN/2015	China	Guangdong	2015	GII
KT970373.1	Norovirus GII isolate Hu/Guangzhou/GZ2015-L337/CHN/2015	China	Guangdong	2015	GII
KT970372.1	Norovirus GII isolate Hu/Guangzhou/GZ2015-L325/CHN/2015	China	Guangdong	2015	GII
KT970371.1	Norovirus GII isolate Hu/Guangzhou/GZ2015-L324/CHN/2015	China	Guangdong	2015	GII

Table S1. *Cont.*

KT970370.1	Norovirus GII isolate Hu/Guangzhou/GZ2014-L313/CHN/2014	China	Guangdong	2014	GII
KT970369.1	Norovirus GII isolate Hu/Guangzhou/GZ2014-L311/CHN/2014	China	Guangdong	2014	GII
JX989073.1	Norovirus Hu/GII.4/GZ2010-L26/Guangzhou/CHN/2010	China	Guangdong	2010	GII.4
KT202798.1	Norovirus GII isolate Hu/Guangzhou/GZ2014-L307/CHN/2014	China	Guangdong	2014	GII
KT202797.1	Norovirus GII isolate Hu/Guangzhou/GZ2014-L295/CHN/2014	China	Guangdong	2014	GII
KT202796.1	Norovirus GII isolate Hu/Guangzhou/GZ2014-L132/CHN/2014	China	Guangdong	2014	GII
KT202795.1	Norovirus GII isolate Hu/Guangzhou/GZ2014-L122/CHN/2014	China	Guangdong	2014	GII
KT202794.1	Norovirus GII isolate Hu/Guangzhou/GZ2014-L106/CHN/2014	China	Guangdong	2014	GII
KT202793.1	Norovirus GII isolate Hu/Guangzhou/GZ2013-L10/CHN/2013	China	Guangdong	2013	GII
cKU724082.1	Norovirus Hu/GII.3/CJ08/2015/CHN	China	Shandong	2015	GII.3
KU724081.1	Norovirus Hu/GI.6/CJ02/2015/CHN	China	Shandong	2015	GI.6
KU724080.1	Norovirus Hu/GI.6/CJ05/2015/CHN	China	Shandong	2015	GI.6
KT634313.1	Norovirus GII.17 strain Hu/GII.17/14042025/CHN/2014	China	Beijing	2014	GII.17
KT633396.1	Norovirus GII.17 strain Hu/GII.17/14112061/CHN/2014	China	Beijing	2014	GII.17
KT633395.1	Norovirus GII.17 strain Hu/GII.17/15142012/CHN/2015	China	Beijing	2015	GII.17
KT633394.1	Norovirus GII.17 strain Hu/GII.17/1501Y007/CHN/2015	China	Beijing	2015	GII.17
KT633393.1	Norovirus GII.17 strain Hu/GII.17/1405Y098/CHN/2014	China	Beijing	2014	GII.17
KT633392.1	Norovirus GII.17 strain Hu/GII.17/1405Y117/CHN/2014	China	Beijing	2014	GII.17
KT633391.1	Norovirus GII.17 strain Hu/GII.17/1405Y139/CHN/2014	China	Beijing	2014	GII.17

Table S1. *Cont.*

KT633390.1	Norovirus GII.17 strain Hu/GII.17/1405Y155/CHN/2014	China	Beijing	2014	GII.17
KT633389.1	Norovirus GII.17 strain Hu/GII.17/1405Y160/CHN/2014	China	Beijing	2014	GII.17
KT633388.1	Norovirus GII.17 strain Hu/GII.17/1406Y348/CHN/2014	China	Beijing	2014	GII.17
KT633387.1	Norovirus GII.17 strain Hu/GII.17/14082073/CHN/2014	China	Beijing	2014	GII.17
KT633386.1	Norovirus GII.17 strain Hu/GII.17/14131084/CHN/2014	China	Beijing	2014	GII.17
KT633385.1	Norovirus GII.17 strain Hu/GII.17/14281084/CHN/2014	China	Beijing	2014	GII.17
KT633384.1	Norovirus GII.17 strain Hu/GII.17/14292035/CHN/2014	China	Beijing	2014	GII.17
KT633383.1	Norovirus GII.17 strain Hu/GII.17/15081009/CHN/2015	China	Beijing	2015	GII.17
KT633382.1	Norovirus GII.17 strain Hu/GII.17/15092003/CHN/2015	China	Beijing	2015	GII.17
KR095172.1	Norovirus Hu/GII.17/15151011/CHN	China	Beijing	2015	GII.17
KR095171.1	Norovirus Hu/GII.17/1501Y002/CHN	China	Beijing	2015	GII.17
KR338890.1	Norovirus GII.17 isolate HU/14F18/PD/SH/CHN/2014	China	Shanghai	2014	GII.17
KR338889.1	Norovirus GII.17 isolate HU/14F17/PD/SH/CHN/2014	China	Shanghai	2014	GII.17
KR338888.1	Norovirus GII.17 isolate HU/14F13/PD/SH/CHN/2014	China	Shanghai	2014	GII.17
KR338887.1	Norovirus GII.17 isolate HU/14F12/PD/SH/CHN/2014	China	Shanghai	2014	GII.17
KR338886.1	Norovirus GII.17 isolate HU/14F9/PD/SH/CHN/2014	China	Shanghai	2014	GII.17
KR338885.1	Norovirus GII.17 isolate HU/14F8/PD/SH/CHN/2014	China	Shanghai	2014	GII.17
KR338884.1	Norovirus GII.17 isolate HU/14F7/PD/SH/CHN/2014	China	Shanghai	2014	GII.17
KR338883.1	Norovirus GII.17 isolate HU/14F6/PD/SH/CHN/2014	China	Shanghai	2014	GII.17
KR338882.1	Norovirus GII.17 isolate HU/14F5/PD/SH/CHN/2014	China	Shanghai	2014	GII.17
KR338881.1	Norovirus GII.17 isolate HU/14F4/PD/SH/CHN/2014	China	Shanghai	2014	GII.17
KR338880.1	Norovirus GII.17 isolate HU/14F3/PD/SH/CHN/2014	China	Shanghai	2014	GII.17
KR338879.1	Norovirus GII.17 isolate HU/14F2/PD/SH/CHN/2014	China	Shanghai	2014	GII.17

Table S1. *Cont.*

KR338878.1	Norovirus GII.17 isolate HU/14F1/PD/SH/CHN/2014	China	Shanghai	2014	GII.17
KR858308.1	Norovirus GII.17 isolate Hu/Norovirus/GII.17/Shunyi-18/Beijing/CHN/2015	China	Beijing	2015	GII.17
KC970257.1	Norovirus Hu/GII.4/Beijing/PKUPH-101/Inpatient/2013/CHN	China	Beijing	2013	GII.4
KC970256.1	Norovirus Hu/GII.4/Beijing/PKUPH-87/Inpatient/2013/CHN	China	Beijing	2013	GII.4
KC970255.1	Norovirus Hu/GII.4/Beijing/PKUPH-54-8/Inpatient/2013/CHN	China	Beijing	2013	GII.4
KC970254.1	Norovirus Hu/GII.4/Beijing/PKUPH-54-7/Inpatient/2013/CHN	China	Beijing	2013	GII.4
KC970253.1	Norovirus Hu/GII.4/Beijing/PKUPH-167/Outpatient/2013/CHN	China	Beijing	2013	GII.4
KC970252.1	Norovirus Hu/GII.4/Beijing/PKUPH-166/Outpatient/2013/CHN	China	Beijing	2013	GII.4
KC970251.1	Norovirus Hu/GII.4/Beijing/PKUPH-160/Outpatient/2013/CHN	China	Beijing	2013	GII.4
KC970250.1	Norovirus Hu/GII.4/Beijing/PKUPH-159/Outpatient/2013/CHN	China	Beijing	2013	GII.4
KC970249.1	Norovirus Hu/GII.4/Beijing/PKUPH-147/Outpatient/2013/CHN	China	Beijing	2013	GII.4
KC970248.1	Norovirus Hu/GII.4/Beijing/PKUPH-140/Outpatient/2013/CHN	China	Beijing	2013	GII.4
KC970247.1	Norovirus Hu/GI.3/Beijing/PKUPH-137/Outpatient/2013/CHN	China	Beijing	2013	GI.3
KC970246.1	Norovirus Hu/GII.4/Beijing/PKUPH-134/Outpatient/2013/CHN	China	Beijing	2013	GII.4
KP335155.1	Norovirus Hu/GII.P17_GII.17/Jinshan15/Shanghai/2014/CHN	China	Beijing	2014	GII.P17_GII.17

Table S1. *Cont.*

KP335154.1	Norovirus Hu/GII.P17/Jinshan19/Shanghai/2014/CHN	China	Shanghai	2014	GII.P17
KP335153.1	Norovirus Hu/GII.P17/Jinshan13/Shanghai/2014/CHN	China	Shanghai	2014	GII.P17
KP335152.1	Norovirus Hu/GII.P17/Jinshan10/Shanghai/2014/CHN	China	Shanghai	2014	GII.P17
KP335151.1	Norovirus Hu/GII.P17/Jinshan09/Shanghai/2014/CHN	China	Shanghai	2014	GII.P17
KP335150.1	Norovirus Hu/GII.P17/Jinshan06/Shanghai/2014/CHN	China	Shanghai	2014	GII.P17
KR269703.1	Norovirus GII.17 isolate water/14Y04/CS/CHN/2014	China	Hunan	2014	GII.17
KR269702.1	Norovirus GII.17 isolate water/14Y03/CS/CHN/2014	China	Hunan	2014	GII.17
KR269701.1	Norovirus GII.17 isolate water/14Y02/CS/CHN/2014	China	Hunan	2014	GII.17
KR269700.1	Norovirus GII.17 isolate water/14Y01/CS/CHN/2014	China	Hunan	2014	GII.17
KR269699.1	Norovirus GII.17 isolate Hu/14Y06/CS/CHN/2014	China	Hunan	2014	GII.17
KR269698.1	Norovirus GII.17 isolate Hu/14Y05/CS/CHN/2014	China	Hunan	2014	GII.17
KR269697.1	Norovirus GII.17 isolate Hu/14Y04/CS/CHN/2014	China	Hunan	2014	GII.17
KR269696.1	Norovirus GII.17 isolate Hu/14Y03/CS/CHN/2014	China	Hunan	2014	GII.17
KR269695.1	Norovirus GII.17 isolate Hu/14Y02/CS/CHN/2014	China	Hunan	2014	GII.17
KR269694.1	Norovirus GII.17 isolate Hu/14Y01/CS/CHN/2014	China	Hunan	2014	GII.17
KM924012.1	Norovirus Hu/GII.P7_GII.6/1415Y002/CHN/2014	China	Beijing	2014	GII.P7_GII.6
KM924011.1	Norovirus Hu/GII.P7_GII.6/1406Y114/CHN/2014	China	Beijing	2014	GII.P7_GII.6
KM924010.1	Norovirus Hu/GII.P7_GII.6/1405Y035/CHN/2014	China	Beijing	2014	GII.P7_GII.6

Table S1. *Cont*.

KM924009.1	Norovirus Hu/GII.P7_GII.7/1406Y012/CHN/2014	China	Beijing	2014	GII.P7_GII.6
KM924008.1	Norovirus Hu/GII.P7_GII.6/1405Y024/CHN/2014	China	Beijing	2014	GII.P7_GII.6
KM924007.1	Norovirus Hu/GII.P7_GII.6/1405Y017/CHN/2014	China	Beijing	2014	GII.P7_GII.6
KM924006.1	Norovirus Hu/GII.P7_GII.6/1403Y001/CHN/2014	China	Beijing	2014	GII.P7_GII.6
KM924005.1	Norovirus Hu/GII.P7_GII.6/1401Y127/CHN/2014	China	Beijing	2014	GII.P7_GII.6
KM924004.1	Norovirus Hu/GII.P6_GII.6/1401Y107/CHN/2014	China	Beijing	2014	GII.P7_GII.6
KM924003.1	Norovirus Hu/GII.P7_GII.7/1401Y082/CHN/2014	China	Beijing	2014	GII.P7_GII.7
KM924002.1	Norovirus Hu/GII.P7_GII.6/1401Y075/CHN/2014	China	Beijing	2014	GII.P7_GII.6
KM924001.1	Norovirus Hu/GII.6/1416Y001/CHN/2014	China	Beijing	2014	GII.6
KC473548.1	Norovirus Hu/GI/HuzhouN10/2008/CHN	China	Zhejiang	2008	GI
KC473547.1	Norovirus Hu/GI/HuzhouN11/2008/CHN	China	Zhejiang	2008	GI
KC473546.1	Norovirus Hu/GII.4/Huzhou128/2012/CHN	China	Zhejiang	2012	GII.4
KC473545.1	Norovirus Hu/GII.4/Huzhou122/2012/CHN	China	Zhejiang	2012	GII.4
KC473544.1	Norovirus Hu/GII.4/Huzhou121/2012/CHN	China	Zhejiang	2012	GII.4
JX644039.1	Norovirus Hu/GII.4/N101/2011/HuZhou	China	Zhejiang	2011	GII.4
JX644038.1	Norovirus Hu/GII.4/N100/2011/HuZhou	China	Zhejiang	2011	GII.4
JX644037.1	Norovirus Hu/GII.4/N95/2011/HuZhou	China	Zhejiang	2011	GII.4
JX644036.1	Norovirus Hu/GII.4/N94/2011/HuZhou	China	Zhejiang	2011	GII.4
JX644035.1	Norovirus Hu/GII.4/N93/2011/HuZhou	China	Zhejiang	2011	GII.4

Table S1. *Cont.*

JX644034.1	Norovirus Hu/GII.4/N91/2011/HuZhou	China	Zhejiang	2011	GII.4
JX644033.1	Norovirus Hu/GII.4/N82/2011/HuZhou	China	Zhejiang	2011	GII.4
JX644032.1	Norovirus Hu/GII.4/N78/2010/HuZhou	China	Zhejiang	2010	GII.4
JX644031.1	Norovirus Hu/GII.4/N76/2010/HuZhou	China	Zhejiang	2010	GII.4
JX644030.1	Norovirus Hu/GII.4/N13/2009/HuZhou	China	Zhejiang	2009	GII.4
JX644029.1	Norovirus Hu/GII.4/N5/2008/HuZhou	China	Zhejiang	2008	GII.4
JX644028.1	Norovirus Hu/GII.4/N4/2008/HuZhou	China	Zhejiang	2008	GII.4
JX644027.1	Norovirus Hu/GII.4/N3/2008/HuZhou	China	Zhejiang	2008	GII.4
JX644026.1	Norovirus Hu/GII.4/N2/2008/HuZhou	China	Zhejiang	2008	GII.4
JX644025.1	Norovirus Hu/GII.4/N8/2008/HuZhou	China	Zhejiang	2008	GII.4
HM195195.1	Norovirus Hu/GII.4/Xiamen/54/2010/CHN	China	Fujian	2010	GII.4
HM195194.1	Norovirus Hu/GII.4/Xiamen/53/2010/CHN	China	Fujian	2010	GII.4
HM195193.1	Norovirus Hu/GII.4/Xiamen/52/2010/CHN	China	Fujian	2010	GII.4
HM195192.1	Norovirus Hu/GII.4/Xiamen/51/2010/CHN	China	Fujian	2010	GII.4
KC894731.1	Norovirus Hu/GIV.1/CCDC GR1113-59/CHN	China	Beijing	2011	GIV.1
JX282191.1	Norovirus Hu/GII/Shenzhen/JB030930009/2009/CHN	China	Guangdong	2009	GII
JN596814.1	Norovirus Hu/GII.3/HZ0708/2009/China	China	Shanghai	2009	GII.3
JN596813.1	Norovirus Hu/GII.3/CQ0378/2009/China	China	Shanghai	2009	GII.3
JN596812.1	Norovirus Hu/GII.3/TJ0738/2009/China	China	Shanghai	2009	GII.3
JN596811.1	Norovirus Hu/GII.3/SH0774/2009/China	China	Shanghai	2009	GII.3
JN596810.1	Norovirus Hu/GII.14/SH0326/2009/China	China	Shanghai	2009	GII.14
JN596809.1	Norovirus Hu/GII.6/TJ0478/2009/China	China	Shanghai	2009	GII.6

Table S1. *Cont.*

JN596808.1	Norovirus Hu/GII.6/HZ1058/2008/China	China	Shanghai	2009	GII.6
JN596807.1	Norovirus Hu/GII.6/CQ0159/2009/China	China	Shanghai	2009	GII.6
JN596806.1	Norovirus Hu/GII.6/SH1157/2008/China	China	Shanghai	2009	GII.6
JN596805.1	Norovirus Hu/GII.4/TJ0779/2009/China	China	Shanghai	2009	GII.4
JN596804.1	Norovirus Hu/GII.4/CQ1046/2008/China	China	Shanghai	2008	GII.4
JN596803.1	Norovirus Hu/GII.4/HZ0730/2009/China	China	Shanghai	2009	GII.4
JN596802.1	Norovirus Hu/GII.4/SH0646/2009/China	China	Shanghai	2009	GII.4
JN596801.1	Norovirus Hu/GII.4/TJ1008/2008/China	China	Shanghai	2008	GII.4
JN596800.1	Norovirus Hu/GII.4/SHO907/2008/China	China	Shanghai	2008	GII.4
JN596799.1	Norovirus Hu/GII.4/HZ1244/2008/China	China	Shanghai	2008	GII.4
JN596798.1	Norovirus Hu/GII.4/CQ0707/2009/China	China	Shanghai	2009	GII.4
JN596797.1	Norovirus Hu/GII.13/TJ1216/2008/China	China	Shanghai	2008	GII.13
JN596796.1	Norovirus Hu/GII.13/CQ0311/2009/China	China	Shanghai	2009	GII.13
JN596794.1	Norovirus Hu/GII.2/CQ0432/2009/China	China	Shanghai	2009	GII.2
JN596793.1	Norovirus Hu/GII.16/TJ1142/2008/China	China	Shanghai	2008	GII.16
JN596792.1	Norovirus Hu/GII.12/SH0450/2009/China	China	Shanghai	2009	GII.12
JN596791.1	Norovirus Hu/GII.12/CQ0470/2009/China	China	Shanghai	2009	GII.12
JN596790.1	Norovirus Hu/GII.12/TJ0128/2009/China	China	Shanghai	2009	GII.12
JN596789.1	Norovirus Hu/GII.7/CQ0248/2009/China	China	Shanghai	2009	GII.7
KT992790.1	Norovirus Hu/GII.17/HNkaohao/Nanyang/CHN/2015	China	Henan	2015	GII.7
KT992789.1	Norovirus Hu/GII.17/HN05/Nanyang/CHN	China	Henan	2015	GII.7
KT992788.1	Norovirus Hu/GII.17/HN04/Nanyang/CHN	China	Henan	2015	GII.7

Table S1. *Cont.*

KT992787.1	Norovirus Hu/GII.17/HN03/Nanyang/CHN	China	Henan	2015	GII.7
KT992786.1	Norovirus Hu/GII.17/HN02/Nanyang/CHN	China	Henan	2015	GII.7
KT992785.1	Norovirus Hu/GII.17/HN01/Nanyang/CHN	China	Henan	2015	GII.7
KF306214.1	Norovirus Hu/GII.4/Jingzhou/2013403/CHN	China	Hubei	2013	GII.4
KF306213.1	Norovirus Hu/GII.3/Jingzhou/2013402/CHN	China	Hubei	2013	GII.3
KF306212.1	Norovirus Hu/GI.2/Jingzhou/2013401/CHN	China	Hubei	2013	GI.2
KC577174.1	Norovirus Hu/GII.4/Jiangsu1/2011/CHN	China	Jiangsu	2011	GII.4
KF586512.1	Norovirus GII.4 isolate CAIQ13040396	China	Beijing	2013	GII.4
GQ223404.1	Norovirus Hu/GII.4/SHZH012/2007/CHN	China	Guangdong	2007	GII.4
GQ223403.1	Norovirus Hu/GII.4/SHZH009/2007/CHN	China	Guangdong	2007	GII.4
GQ223402.1	Norovirus Hu/GII.4/SHZH004/2007/CHN	China	Guangdong	2007	GII.4
GQ223401.1	Norovirus Hu/GII.4/SHZH168/2007/CHN	China	Guangdong	2007	GII.4
GQ223400.1	Norovirus Hu/GII.4/SHZH154/2007/CHN	China	Guangdong	2007	GII.4
GQ223399.1	Norovirus Hu/GII.4/SHZH166/2007/CHN	China	Guangdong	2007	GII.4
KP676383.1	Norovirus Hu/GII/CN/2013/GII.P17_GII.17/Nanjing010141	China	Jiangsu	2013	GII.P17_GII.17
EU839595.1	Norovirus Hu/GII/Beijing/362/2007/CHN	China	Beijing	2007	GII
EU839594.1	Norovirus Hu/GII/Beijing/361/2007/CHN	China	Beijing	2007	GII
EU839593.1	Norovirus Hu/GII/Beijing/321/2007/CHN	China	Beijing	2007	GII
EU839592.1	Norovirus Hu/GII/Beijing/127/2007/CHN	China	Beijing	2007	GII
EU839591.1	Norovirus Hu/GII/Beijing/125/2007/CHN	China	Beijing	2007	GII
EU839590.1	Norovirus Hu/GII/Beijing/116/2007/CHN	China	Beijing	2007	GII
EU839589.1	Norovirus Hu/GII/Beijing/34/2006/CHN	China	Beijing	2006	GII

Table S1. *Cont.*

EU839588.1	Norovirus Hu/GII/Beijing/07/2006/CHN	China	Beijing	2006	GII
EU839587.1	Norovirus Hu/GII/Beijing/493/2005/CHN	China	Beijing	2006	GII
EU839586.1	Norovirus Hu/GII/Beijing/484/2005/CHN	China	Beijing	2006	GII
EU839585.1	Norovirus Hu/GII/Beijing/274/2005/CHN	China	Beijing	2005	GII
EU839584.1	Norovirus Hu/GII/Beijing/221/2005/CHN	China	Beijing	2005	GII
EU839583.1	Norovirus Hu/GII/Beijing/79/2004/CHN	China	Beijing	2004	GII
EU839582.1	Norovirus Hu/GII/Beijing/39/2004/CHN	China	Beijing	2004	GII
EU839581.1	Norovirus Hu/GII/Beijing/30/2004/CHN	China	Beijing	2004	GII
JX155754.1	Norovirus Hu/GII.4/Xi'an/C45/2010/CHN	China	Sshanxi	2010	GII.4
JX155753.1	Norovirus Hu/GII.4/Xi'an/C41/2010/CHN	China	Sshanxi	2010	GII.4
JX155752.1	Norovirus Hu/GII.4/Xi'an/C31/2010/CHN	China	Sshanxi	2010	GII.4
JX155751.1	Norovirus Hu/GII.4/Xi'an/C30/2010/CHN	China	Sshanxi	2010	GII.4
JX155750.1	Norovirus Hu/GII.4/Xi'an/C21/2010/CHN	China	Sshanxi	2010	GII.4
JX155749.1	Norovirus Hu/GII.4/Xi'an/C15/2010/CHN	China	Sshanxi	2010	GII.4
JX155748.1	Norovirus Hu/GII.4/Xi'an/C12/2010/CHN	China	Sshanxi	2010	GII.4
JX155747.1	Norovirus Hu/GII.4/Xi'an/C6/2010/CHN	China	Sshanxi	2010	GII.4
JX155746.1	Norovirus Hu/GII.4/Xi'an/P185/2010/CHN	China	Sshanxi	2010	GII.4
JX155745.1	Norovirus Hu/GII.4/Xi'an/P158/2010/CHN	China	Sshanxi	2010	GII.4
JX155744.1	Norovirus Hu/GII.4/Xi'an/P154/2010/CHN	China	Sshanxi	2010	GII.4
JX155743.1	Norovirus Hu/GII.4/Xi'an/P152/2010/CHN	China	Sshanxi	2010	GII.4
JX155742.1	Norovirus Hu/GII.4/Xi'an/P143/2010/CHN	China	Sshanxi	2010	GII.4
JX155741.1	Norovirus Hu/GII.4/Xi'an/P131/2010/CHN	China	Sshanxi	2010	GII.4
JX155740.1	Norovirus Hu/GII.4/Xi'an/P128/2010/CHN	China	Sshanxi	2010	GII.4

Table S1. *Cont.*

JX155739.1	Norovirus Hu/GII.4/Xi'an/P49/2010/CHN	China	Sshanxi	2010	GII.4
JX155738.1	Norovirus Hu/GII.4/Xi'an/P46/2010/CHN	China	Sshanxi	2010	GII.4
JX155737.1	Norovirus Hu/GII.4/Xi'an/P19/2010/CHN	China	Sshanxi	2010	GII.4
KC894943.1	Norovirus Hu/GII.4/Guangzhou/GZ2010-L91/CHN/2011	China	Guangdong	2011	GII.4
KC894942.1	Norovirus Hu/GII.4/Guangzhou/GZ2010-L88/CHN/2011	China	Guangdong	2011	GII.4
JQ934820.1	Norovirus Hu/GII.g/GuizhouCDC397/Guizhou/2010/CNA	China	Guizhou	2010	GII.g
JQ934819.1	Norovirus Hu/GII.3/GuizhouCDC62/Guizhou/2010/CNA	China	Guizhou	2010	GII.3
JQ934818.1	Norovirus Hu/GII.3/GuizhouCDC44/Guizhou/2010/CNA	China	Guizhou	2010	GII.3
JQ934817.1	Norovirus Hu/GII.3/GuizhouCDC42/Guizhou/2010/CNA	China	Guizhou	2010	GII.3
JQ934816.1	Norovirus Hu/GII.3/GuizhouCDC33/Guizhou/2010/CNA	China	Guizhou	2010	GII.3
JQ934815.1	Norovirus Hu/GII.4/GuizhouCDC425/Guizhou/2010/CNA	China	Guizhou	2010	GII.4
JQ934814.1	Norovirus Hu/GII.4/GuizhouCDC415/Guizhou/2010/CNA	China	Guizhou	2010	GII.4
JQ934813.1	Norovirus Hu/GII.4/GuizhouCDC403/Guizhou/2010/CNA	China	Guizhou	2010	GII.4
JQ934812.1	Norovirus Hu/GII.4/GuizhouCDC390/Guizhou/2010/CNA	China	Guizhou	2010	GII.4
JQ934811.1	Norovirus Hu/GII.4/GuizhouCDC351/Guizhou/2010/CNA	China	Guizhou	2010	GII.4
JQ934810.1	Norovirus Hu/GII.4/GuizhouCDC312/Guizhou/2010/CNA	China	Guizhou	2010	GII.4

Table S1. *Cont.*

JQ934809.1	Norovirus Hu/GII.4/GuizhouCDC300/Guizhou/2010/CNA	China	Guizhou	2010	GII.4
JQ934808.1	Norovirus Hu/GII.4/GuizhouCDC292/Guizhou/2010/CNA	China	Guizhou	2010	GII.4
JQ934807.1	Norovirus Hu/GII.4/GuizhouCDC239/Guizhou/2010/CNA	China	Guizhou	2010	GII.4
KT716755.1	Norovirus Hu/GII.17/JB031520278/CHN/2015	China	Hubei	2015	GII.17
KT716754.1	Norovirus Hu/GII.17/JB031520258/CHN/2015	China	Hubei	2015	GII.17
KT716753.1	Norovirus Hu/GII.17/JB031520249/CHN/2015	China	Hubei	2015	GII.17
KT716752.1	Norovirus Hu/GII.17/JB031520198/CHN/2015	China	Hubei	2015	GII.17
KT716751.1	Norovirus Hu/GII.17/JB031520197/CHN/2015	China	Hubei	2015	GII.17
KT716750.1	Norovirus Hu/GII.17/JB031520193/CHN/2015	China	Hubei	2015	GII.17
KT716749.1	Norovirus Hu/GII.17/JB031520189/CHN/2015	China	Hubei	2015	GII.17
KT716748.1	Norovirus Hu/GII.17/JB031520150/CHN/2015	China	Hubei	2015	GII.17
KT716747.1	Norovirus Hu/GII.17/JB031520137/CHN/2015	China	Hubei	2015	GII.17
KT716746.1	Norovirus Hu/GII.17/JB031520120/CHN/2015	China	Hubei	2015	GII.17
KT716745.1	Norovirus Hu/GII.17/JB031520105/CHN/2015	China	Hubei	2015	GII.17
KT716744.1	Norovirus Hu/GII.17/JB031520104/CHN/2015	China	Hubei	2015	GII.17
KT716743.1	Norovirus Hu/GII.17/JB031520066/CHN/2015	China	Hubei	2015	GII.17
KT716742.1	Norovirus Hu/GII.17/JB031520060/CHN/2015	China	Hubei	2015	GII.17
KT716741.1	Norovirus Hu/GII.17/JB031520054/CHN/2015	China	Hubei	2015	GII.17
KT716740.1	Norovirus Hu/GII.17/JB031520046/CHN/2015	China	Hubei	2015	GII.17
KT716739.1	Norovirus Hu/GII.17/JB031520043/CHN/2015	China	Hubei	2015	GII.17
KT716738.1	Norovirus Hu/GII.17/JB031520041/CHN/2015	China	Hubei	2015	GII.17

Table S1. *Cont.*

KT716737.1	Norovirus Hu/GII.17/JB031520038/CHN/2015	China	Hubei	2015	GII.17
KT716736.1	Norovirus Hu/GII.17/JB031520026/CHN/2015	China	Hubei	2015	GII.17
KT716735.1	Norovirus Hu/GII.17/JB031421150/CHN/2014	China	Hubei	2014	GII.17
KT716734.1	Norovirus Hu/GII.17/JB031421102/CHN/2014	China	Hubei	2014	GII.17
KT716733.1	Norovirus Hu/GII.17/JB031421095/CHN/2014	China	Hubei	2014	GII.17
KT716732.1	Norovirus Hu/GII.17/JB031421094/CHN/2014	China	Hubei	2014	GII.17
KT716731.1	Norovirus Hu/GII.17/JB031421092/CHN/2014	China	Hubei	2014	GII.17
KT716730.1	Norovirus Hu/GII.17/JB031421004/CHN/2014	China	Hubei	2014	GII.17
KT716729.1	Norovirus Hu/GII.17/JB031421000/CHN/2014	China	Hubei	2014	GII.17
KT716728.1	Norovirus Hu/GII.17/JB031420995/CHN/2014	China	Hubei	2014	GII.17
KT716727.1	Norovirus Hu/GII.17/JB031420994/CHN/2014	China	Hubei	2014	GII.17
KT716726.1	Norovirus Hu/GII.17/JB031420990/CHN/2014	China	Hubei	2014	GII.17
KT716725.1	Norovirus Hu/GII.17/JB031420921/CHN/2014	China	Hubei	2014	GII.17
KT716724.1	Norovirus Hu/GII.17/JB031420383/CHN/2014	China	Hubei	2014	GII.17
KT716723.1	Norovirus Hu/GII.17/JB031530044/CHN/2015	China	Hubei	2015	GII.17
KT716722.1	Norovirus Hu/GII.17/JB031530043/CHN/2015	China	Hubei	2015	GII.17
KT716721.1	Norovirus Hu/GII.17/JB031530042/CHN/2015	China	Hubei	2015	GII.17
KT716720.1	Norovirus Hu/GII.17/JB031530041/CHN/2015	China	Hubei	2015	GII.17
KT716719.1	Norovirus Hu/GII.17/JB031530039/CHN/2015	China	Hubei	2015	GII.17
KT716718.1	Norovirus Hu/GII.17/JB031530037/CHN/2015	China	Hubei	2015	GII.17
KT716717.1	Norovirus Hu/GII.17/JB031530026/CHN/2015	China	Hubei	2015	GII.17
KT716716.1	Norovirus Hu/GII.17/JB031530021/CHN/2015	China	Hubei	2015	GII.17
KT716715.1	Norovirus Hu/GII.17/JB031530020/CHN/2015	China	Hubei	2015	GII.17

Table S1. *Cont.*

KT716714.1	Norovirus Hu/GII.17/JB031530017/CHN/2015	China	Hubei	2015	GII.17
KT716713.1	Norovirus Hu/GII.17/JB031530025/CHN/2014	China	Hubei	2014	GII.17
KT716712.1	Norovirus Hu/GII.17/JB031530024/CHN/2014	China	Hubei	2014	GII.17
KT716711.1	Norovirus Hu/GII.17/JB031530023/CHN/2014	China	Hubei	2014	GII.17
KT716710.1	Norovirus Hu/GII.17/JB031430111/CHN/2014	China	Hubei	2014	GII.17
KT716709.1	Norovirus Hu/GII.17/JB031430110/CHN/2014	China	Hubei	2014	GII.17
KT716708.1	Norovirus Hu/GII.17/JB031430109/CHN/2014	China	Hubei	2014	GII.17
KT716707.1	Norovirus Hu/GII.17/JB031430098/CHN/2014	China	Hubei	2014	GII.17
KT716706.1	Norovirus Hu/GII.17/JB031430006/CHN/2014	China	Hubei	2014	GII.17
KT716705.1	Norovirus Hu/GII.17/JB031430005/CHN/2014	China	Hubei	2014	GII.17
KT716704.1	Norovirus Hu/GII.17/JB031430004/CHN/2014	China	Hubei	2014	GII.17
GQ379155.1	Norovirus Hu/GII/Tianjin/510/2009/CHN	China	Tianjin	2009	GII.3
KP325656.1	Norovirus Hu/GI.2/SH20140420-G29/Shanghai/2014/CHN	China	Shanghai	2014	GI.2
KP325655.1	Norovirus Hu/GI.5/SH20140415-G25/Shanghai/2014/CHN	China	Shanghai	2014	GI.5
KP325654.1	Norovirus Hu/GI.2/SH20140414-G24/Shanghai/2014/CHN	China	Shanghai	2014	GI.2
KP325653.1	Norovirus Hu/GI.2/SH20140420-G23/Shanghai/2014/CHN	China	Shanghai	2014	GI.2
KP325652.1	Norovirus Hu/GI.2/SH20140415-G21/Shanghai/2014/CHN	China	Shanghai	2014	GI.2
KP325651.1	Norovirus Hu/GI.5/SH20140415-G20/Shanghai/2014/CHN	China	Shanghai	2014	GI.5
KP325650.1	Norovirus Hu/GI.5/SH20140414-G19/Shanghai/2014/CHN	China	Shanghai	2014	GI.5
KP325649.1	Norovirus Hu/GI.2/SH20140415-G17/Shanghai/2014/CHN	China	Shanghai	2014	GI.2
KP325648.1	Norovirus Hu/GI.2/SH20140414-G14/Shanghai/2014/CHN	China	Shanghai	2014	GI.2
KP325647.1	Norovirus Hu/GI.5/SH20140414-G13/Shanghai/2014/CHN	China	Shanghai	2014	GI.5
KP325646.1	Norovirus Hu/GI.2/SH20140414-G11/Shanghai/2014/CHN	China	Shanghai	2014	GI.2

Table S1. *Cont.*

KP325645.1	Norovirus Hu/GI.5/SH20140415-G10/Shanghai/2014/CHN	China	Shanghai	2014	GI.5
KP325644.1	Norovirus Hu/GI.2/SH20140415-G6/Shanghai/2014/CHN	China	Shanghai	2014	GI.2
KP325643.1	Norovirus Hu/GI.2/SH20140415-G5/Shanghai/2014/CHN	China	Shanghai	2014	GI.2
KP325642.1	Norovirus Hu/GI.2/SH20140414-G2/Shanghai/2014/CHN	China	Shanghai	2014	GI.2
KP718702.1	Norovirus GII.17 isolate Hu/15F95/ZQ/GD/CHN/2015	China	Guangdong	2015	GII.17
KP718701.1	Norovirus GII.17 isolate Hu/15F93/ZH/GD/CHN/2015	China	Guangdong	2015	GII.17
KP718700.1	Norovirus GII.17 isolate Hu/15F91/ZH/GD/CHN/2015	China	Guangdong	2015	GII.17
KP718699.1	Norovirus GII.17 isolate Hu/15F89/ZH/GD/CHN/2015	China	Guangdong	2015	GII.17
KP718698.1	Norovirus GII.17 isolate Hu/15F82/ZH/GD/CHN/2015	China	Guangdong	2015	GII.17
KP718697.1	Norovirus GII.17 isolate Hu/15F81/ZH/GD/CHN/2015	China	Guangdong	2015	GII.17
KP718696.1	Norovirus GII.17 isolate Hu/15F80/ZH/GD/CHN/2015	China	Guangdong	2015	GII.17
KP718695.1	Norovirus GII.17 isolate Hu/15F79/ZH/GD/CHN/2015	China	Guangdong	2015	GII.17
KP718694.1	Norovirus GII.17 isolate Hu/15F77/ZH/GD/CHN/2015	China	Guangdong	2015	GII.17
KP718693.1	Norovirus GII.17 isolate Hu/15F74/ZH/GD/CHN/2015	China	Guangdong	2015	GII.17
KP718692.1	Norovirus GII.17 isolate Hu/15F73/ZH/GD/CHN/2015	China	Guangdong	2015	GII.17
KP718691.1	Norovirus GII.17 isolate Hu/15F72/ZH/GD/CHN/2015	China	Guangdong	2015	GII.17
KP718690.1	Norovirus GII.17 isolate Hu/15F67/ZH/GD/CHN/2015	China	Guangdong	2015	GII.17
KP718689.1	Norovirus GII.17 isolate Hu/15F62/ZH/GD/CHN/2015	China	Guangdong	2015	GII.17
KP718688.1	Norovirus GII.17 isolate Hu/15F58/ZH/GD/CHN/2015	China	Guangdong	2015	GII.17
KP718687.1	Norovirus GII.17 isolate Hu/15F57/ZH/GD/CHN/2015	China	Guangdong	2015	GII.17
KP718686.1	Norovirus GII.17 isolate Hu/15F56/ZH/GD/CHN/2015	China	Guangdong	2015	GII.17
KP718685.1	Norovirus GII.17 isolate Hu/15F55/ZH/GD/CHN/2015	China	Guangdong	2015	GII.17
KP718684.1	Norovirus GII.17 isolate Hu/15F54/ZH/GD/CHN/2015	China	Guangdong	2015	GII.17

Table S1. *Cont.*

KP718683.1	Norovirus GII.17 isolate Hu/15F48/ZH/GD/CHN/2015	China	Guangdong	2015	GII.17
KP718682.1	Norovirus GII.17 isolate Hu/15F42/ZH/GD/CHN/2015	China	Guangdong	2015	GII.17
KP718681.1	Norovirus GII.17 isolate Hu/15F33/ZH/GD/CHN/2015	China	Guangdong	2015	GII.17
KP718680.1	Norovirus GII.17 isolate Hu/15F32/ZH/GD/CHN/2015	China	Guangdong	2015	GII.17
KP718679.1	Norovirus GII.17 isolate Hu/15F23/HZ/GD/CHN/2015	China	Guangdong	2015	GII.17
KP718678.1	Norovirus GII.17 isolate Hu/15F22/HZ/GD/CHN/2015	China	Guangdong	2015	GII.17
KP718677.1	Norovirus GII.17 isolate Hu/15F19/HZ/GD/CHN/2015	China	Guangdong	2015	GII.17
KP718676.1	Norovirus GII.17 isolate Hu/15F18/HZ/GD/CHN/2015	China	Guangdong	2015	GII.17
KP718675.1	Norovirus GII.17 isolate Hu/15F17/HZ/GD/CHN/2015	China	Guangdong	2015	GII.17
KP718674.1	Norovirus GII.17 isolate Hu/15F16/HY/GD/CHN/2015	China	Guangdong	2015	GII.17
KP718673.1	Norovirus GII.17 isolate Hu/15F15/HY/GD/CHN/2015	China	Guangdong	2015	GII.17
KP718672.1	Norovirus GII.17 isolate Hu/15F14/HY/GD/CHN/2015	China	Guangdong	2015	GII.17
KP718671.1	Norovirus GII.17 isolate Hu/15F13/HY/GD/CHN/2015	China	Guangdong	2015	GII.17
KP718670.1	Norovirus GII.17 isolate Hu/15F10/HY/GD/CHN/2015	China	Guangdong	2015	GII.17
KP994317.1	Norovirus GII.17 isolate Hu/NoV/GD15/2015/Guangdong/ China	China	Guangdong	2015	GII.17
KT253245.1	Norovirus Hu/GII.17/CHN/2015/ZHITHC-12	China	Guangdong	2015	GII.17
KT970371.1	Norovirus GII isolate Hu/Guangzhou/GZ2015-L324/CHN/2015	China	Guangdong	2015	GII.17
KR869086.1	Norovirus Hu/Guangzhou/GZ2015-L362/CHN/2015	China	Guangdong	2015	GII.17
KR869085.1	Norovirus Hu/Guangzhou/GZ2015-L343/CHN/2015	China	Guangdong	2015	GII.17
KR869084.1	Norovirus Hu/Guangzhou/GZ2015-L340/CHN/2015	China	Guangdong	2015	GII.17

Table S1. *Cont*.

KR869083.1	Norovirus Hu/Guangzhou/GZ2015-L339/CHN/2015	China	Guangdong	2015	GII.17
KR869082.1	Norovirus Hu/Guangzhou/GZ2015-L337/CHN/2015	China	Guangdong	2015	GII.17
KR869081.1	Norovirus Hu/Guangzhou/GZ2015-L336/CHN/2015	China	Guangdong	2015	GII.17
KR869079.1	Norovirus Hu/Guangzhou/GZ2015-L325/CHN/2015	China	Guangdong	2015	GII.17
KR869078.1	Norovirus Hu/Guangzhou/GZ2015-L324/CHN/2015	China	Guangdong	2015	GII.17
KR869080.1	Norovirus Hu/Guangzhou/GZ2015-L335/CHN/2015	China	Guangdong	2015	GII.17

© 2016 by the authors; licensee MDPI, Basel, Switzerland. This article is an open access article distributed under the terms and conditions of the Creative Commons by Attribution (CC-BY) license (http://creativecommons.org/licenses/by/4.0/).
